# Catalytic Amine Oxidation under Ambient Aerobic Conditions: Mimicry of Monoamine Oxidase B**

**DOI:** 10.1002/anie.201503654

**Published:** 2015-06-18

**Authors:** Alexander T Murray, Myles J H Dowley, Fabienne Pradaux-Caggiano, Amgalanbaatar Baldansuren, Alistair J Fielding, Floriana Tuna, Christopher H Hendon, Aron Walsh, Guy C Lloyd-Jones, Matthew P John, David R Carbery

**Affiliations:** Department of Chemistry, University of Bath Claverton Down, Bath (UK) E-mail: d.carbery@bath.ac.uk; EPSRC National EPR Facility, Photon Science Institute, School of Chemistry, University of Manchester Oxford Road, Manchester (UK); School of Chemistry, Joseph Black Building West Mains Road, Edinburgh EH9 3 JJ (UK); GlaxoSmithKline Research and Development Gunnels Wood Road, Stevenage (UK)

**Keywords:** amines, enzymes, EPR spectroscopy, oxidation, reaction mechanisms

## Abstract

The flavoenzyme monoamine oxidase (MAO) regulates mammalian behavioral patterns by modulating neurotransmitters such as adrenaline and serotonin. The mechanistic basis which underpins this enzyme is far from agreed upon. Reported herein is that the combination of a synthetic flavin and alloxan generates a catalyst system which facilitates biomimetic amine oxidation. Mechanistic and electron paramagnetic (EPR) spectroscopic data supports the conclusion that the reaction proceeds through a radical manifold. This data provides the first example of a biorelevant synthetic model for monoamine oxidase B activity.

Monoamine oxidase (MAO) is a mitochondrial flavin-dependent oxidoreductase enzyme which oxidizes a range of important amines to imines, for example, the neurotransmitters serotonin, histamine, and noradrenaline.[1] With such an integral role in the neurochemical network, MAO function has been pinpointed as an underlying rationale for a range of behavioral, evolutionary, and physiological observations. For example, variations in the MAO A gene can lead to increased aggression, known as the “warrior gene”, ultimately impacting human evolution.[2] Inhibition of MAO has been an important area for medicinal chemistry with MAO inhibitors (MAOIs) acting as potent antidepressants and having potential applications as neuroprotective agents.[3] Mechanistic studies have also helped in understanding the role of lysine-specific demethylase 1 (LSD1), a key epigenetic modulator, with MAOIs impacting a number of key biological processes.[4]

It is remarkable that no consensus has been reached with respect to a mechanism of action, despite over 45 years of investigation.[5] There are two isozymes of MAO: MAO-A and MAO-B. While the flavin active sites are identical, each form displays a different substrate and inhibitor profile, and the mechanistic basis of this selectivity unknown.[6]

^2^H primary kinetic isotope (KIE) effects have been observed for the C–H bond cleavage step(s) with both MAO A and B. In principle, rate-contributing cleavage may be envisaged as proceeding by either H^+^-, H^−^-, or H^.^-transfer mechanisms (Scheme 1). These options have been widely discussed,[5] with rate-contributing C–H cleavage by H^+^ transfer being the most prevalent mechanistic description. Two mechanistic postulates have been developed to account for the requisite increase in acidity of the relevant α-amino C–H bond: the formation of a covalent flavin–amine conjugate,[7] and the formation of an aminium radical cation[8] after single-electron transfer from amine to flavin. As both mechanisms require discrete steps prior to the rate-contributing C–H cleavage, it is notable that no intermediates accumulate to observable populations. C–H cleavage in the context of a direct hydride transfer has also been suggested.[9] However, such a synchronous event would not be consistent with the ^15^N KIE measured for amine oxidation by MAO B, thus pointing to an absence of synchronicity between C–H cleavage and sp^2^→sp^3^ nitrogen atom re-hybridization.[10] Finally, H^.^ transfer from the substrate to the flavin has been suggested.[11] This possibility was discarded on the grounds that no hydrogen-atom abstracting moiety, which was reactive enough to overcome relevant α-amino C–H bond dissociation energies, could be identified in the enzyme active site.[12]

**Scheme 1:**
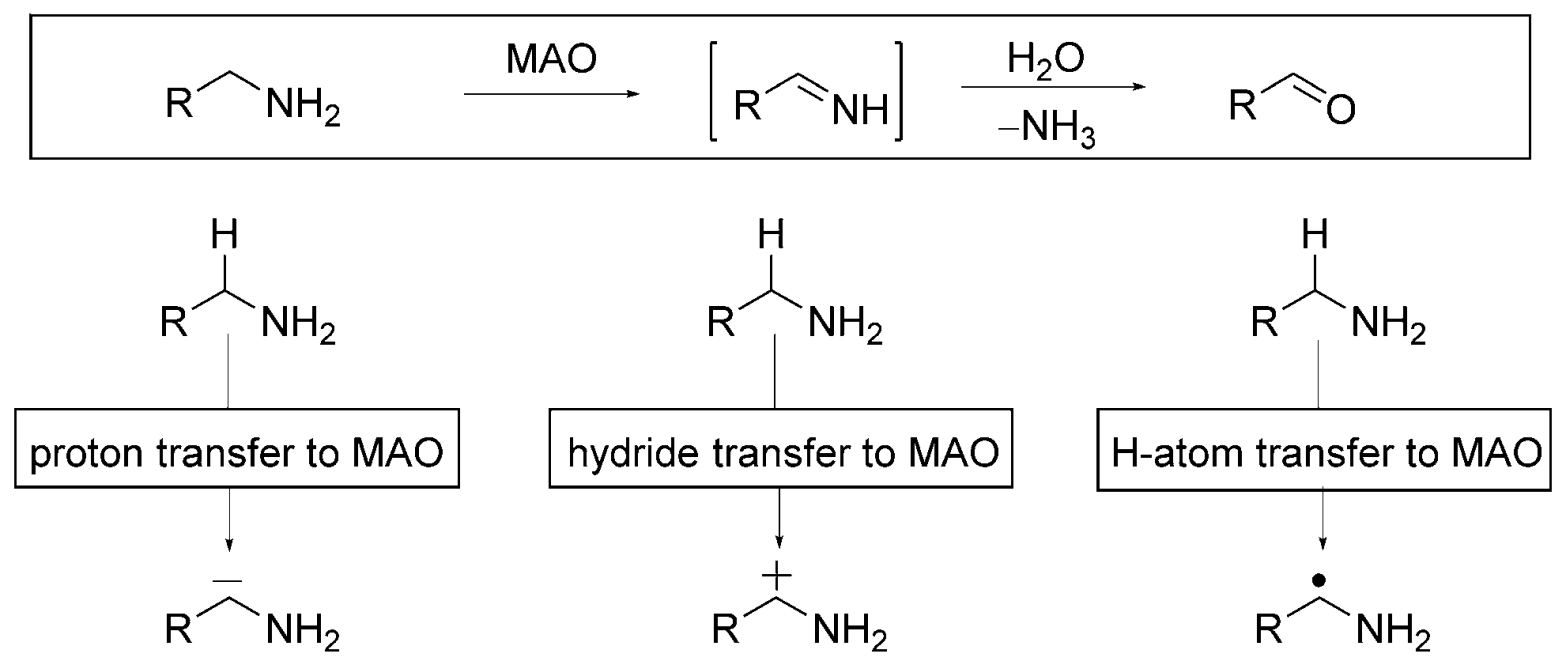
MAO-catalyzed oxidation of amines and qualitative overview of possible modes of C–H bond cleavage.

Studies using synthetic flavins have played a crucial role in elucidating flavoenzyme mechanisms.[13] Accordingly, insight gained from studying model cofactors is a valid strategy to unlocking mechanistic problems in flavoenzymology. Pioneering work on primary amines by various groups supported the polar, proton-transfer mechanism, but the low turnover, tendency of catalysts to decompose, and requirement of heating in an enriched O_2_ atmosphere for several days meant that they are perhaps of limited relevance to biological processes.[14] We,[15] and others,[16] have previously applied cationic flavin catalysts in biomimetic monooxygenase contexts, as well as donor–acceptor chemistry,[17] and now report the oxidation of biologically pertinent amines as a vehicle to understanding MAO mechanism.

Initial exploratory studies demonstrated catalytic aerobic oxidation of benzylamine, with formation of the imine **4 a** being consistent with oxidase rather than monooxygenase-like reactivity (Table 1).[18] Excellent yields of **4 a** were obtained if a thioether additive (Me_2_S) and a cocatalyst, alloxan (**3 a**), were used (Table 1). Initially **3 a** was present as an undetected by-product from the synthesis of **2 a**, however, was found to be crucial for this transformation. *N,N*-dimethylalloxan (**3 b**) was found to be inactive (entry 3) despite possessing structural similarity to **3 a**. Additionally, cobalamin synthase, BluB, has been implicated in the cannibalization of flavin mononucleotide to form alloxan, which acts as a crucial multifunctional redox catalyst in the biosynthesis of vitamin B12.^[19]^ A series of substituted benzylamines, typical substrates for MAO-B, have been examined. Generally, high yields of imine products are attainable, although substrates with a strongly electron-withdrawing *para*-substituent group (entry 9) are less reactive, thus mirroring MAO B reactivity trends.

**Table 1:** Flavin-organocatalyzed amine oxidation^[a]^ 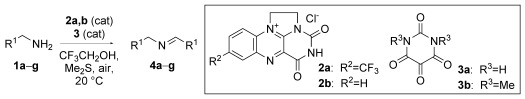

Entry	Substrate	R^1^	Product	Yield [%]
1	**1 a**	Ph	**4 a**	94
2^[b]^	**1 a**	Ph	**4 a**	49
3^[c]^	**1 a**	Ph	**–**	0
4	**1 b**	4*-*MeC_6_H_4_	**4 b**	99
5	**1 c**	4*-*MeOC_6_H_4_	**4 c**	95
6	**1 d**	4*-t*BuC_6_H_4_	**4 d**	92
7^[d]^	**1 e**	4-FC_6_H_4_	**4 e**	98
8^[d]^	**1 f**	4*-*ClC_6_H_4_	**4 f**	77
9^[d,e]^	**1 g**	4-CF_3_C_6_H_4_	**4 g**	37
10	**1 h**	3-MeC_6_H_4_	**4 h**	91
11	**1 i**	3-OMeC_6_H_4_	**4 i**	72
12	**1 j**	2-MeC_6_H_4_	**4 j**	96
13	**1 k**	2-MeOC_6_H_4_	**4 k**	68
14^[d]^	**1 l**	2-ClC_6_H_4_	**4 l**	66
15^[d]^	**1 m**	2-furyl	**4 m**	70
16	**1 n**	2-thiophenyl	**4 n**	87
17	**1 o**	1-naphthyl	**4 o**	72

[a] Reaction conditions: **2 a** (2 mol %), **3 a** (2 mol %), Me_2_S (10 equiv), 5 h. [b] **2 b** used. [c] **3 b** used. [d] 18 h. [e] **2 a** (4 mol %), **3 a** (4 mol %) used.

Upon attempted in situ ^1^H NMR analysis, the inability to locate the lock signal suggested paramagnetic behavior. Accordingly, EPR studies at the X-band were initiated. Mixing **2 a** and Me_2_S generated the flavin radical cation **2 a′** (Figure 1). The structure was further confirmed by pulsed EPR studies. In particular, the protonation state of **2 a′** was assessed by electron spin echo envelope modulation (ESEEM), and is a rare example of an aerobically generated flavin semiquinone, having demonstrable relevance to catalysis, observed by EPR spectroscopy.[20] The use of a strong hydrogen-bonding solvent, trifluoroethanol, may aid the stabilization of the semiquinone formation, as discussed by Massey and co-workers, for flavins with amino acids.[21] Upon sequential addition of alloxan and amine, a new EPR spectrum was observed and characterized as the radical **4 a**′, and is consistent with charge-transfer-initiated hydrogen-atom abstraction from **4 a**. Hybrid-DFT and post-Hartree Fock calculations were performed on **2 a′** and **4 a**′ and the spin density isosurfaces are shown in Figure 1.[22] Importantly, the theoretical calculations quantify the local spin density distribution, thus further corroborating the simulations of the continuous-wave EPR spectra.[23]

**Figure 1:**
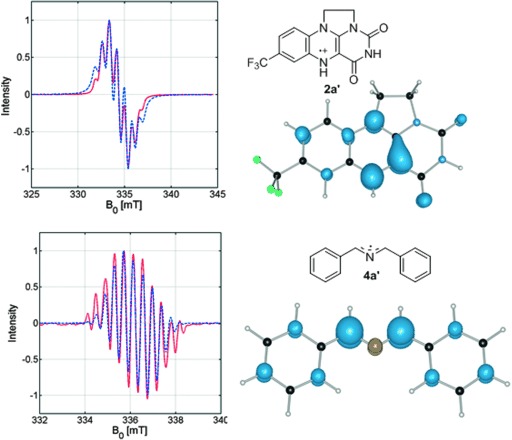
EPR spectra of 2 a′ and 4 a′, and DFT-calculated spin densities measured from solutions of 2 a + Me_2_S (top) and 2 a + Me_2_S + 3 a + 1 a (bottom).

Kinetic studies provided additional important mechanistic information with the transformation being first order in benzylamine[24] and showing a KIE of *k*_H_/*k*_D_=1.9 when using PhCD_2_NH_2_ (**7**), thus supporting rate-contributing C–H bond cleavage (Figure 2). A range of studied *para*-substituted benzylamines provided a negative Hammett correlation (*ρ*=−2).

**Figure 2:**
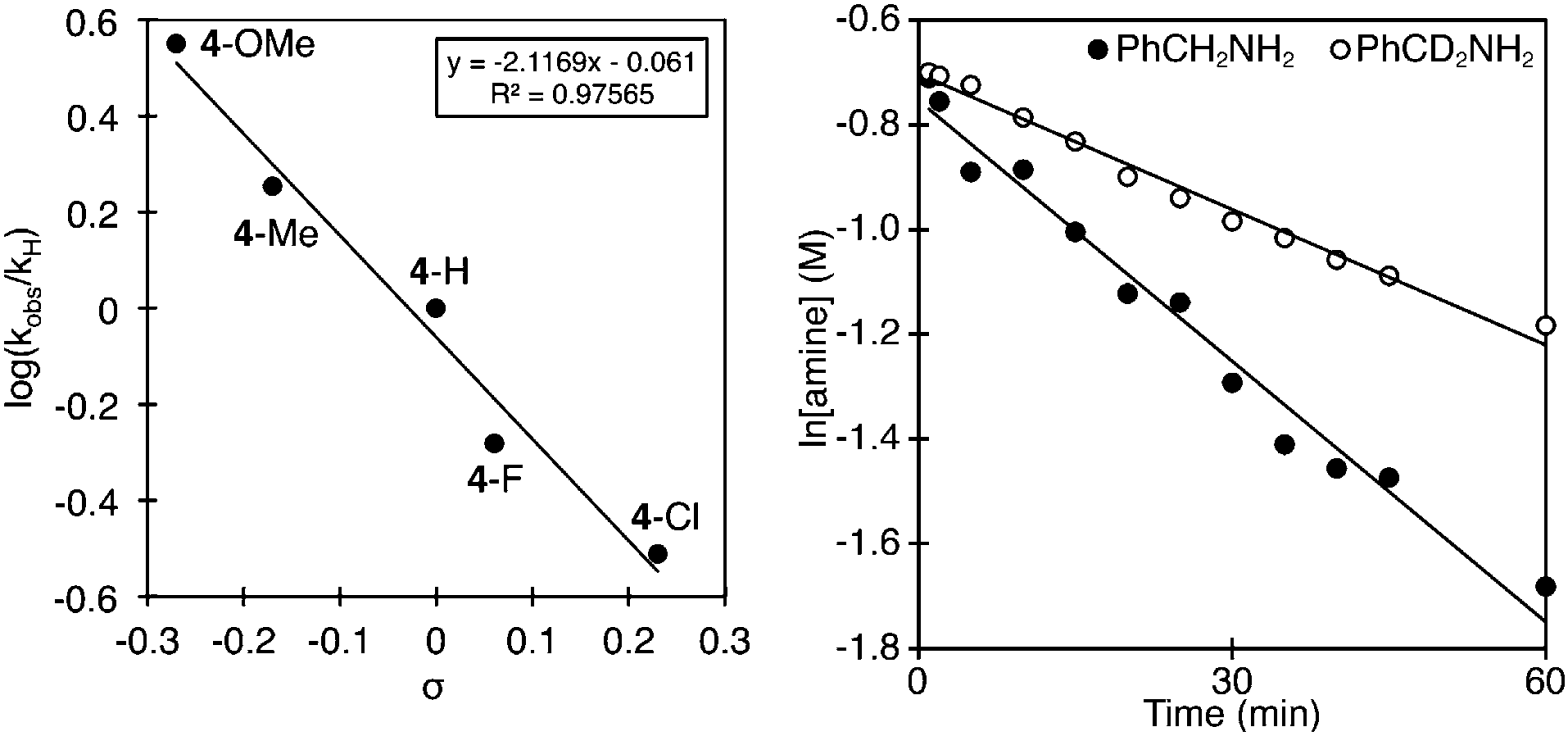
Hammett and kinetic isotope effect study carried out by HPLC analysis of imine formation from amines against an internal standard of naphthalene.

The observed rates of reaction were found to be independent of the Me_2_S concentration. Kinetic analysis for **3 a** did not demonstrate a simple reaction order, with saturation behavior observed over the concentrations examined (see the Supporting Information). The kinetic order in **2 a** was probed by means of a ln(*k*_obs_) versus ln([flavin]) plot, which was linear with a slope of 0.25 and consistent with de-aggregation of a higher order resting state, but with a monomeric semiquinone being catalytically active. Significantly, the less oxidizing flavin **2 b** also mediates this reaction (*k*_obs**2a**_/*k*_obs**2b**_=2.96) with an electrochemical reduction potential of +66 mV vs. SHE, which parallels MAO-B at +40 mV (Table 1, entry 2)[25] Therefore, the flavin catalysts **2 a**,**b** offer themselves as realistic mimics of MAO through the neutral N(5)-H semiquinone.[26]

A mechanism that accounts for EPR and kinetic data is underpinned by the realization that rate-determining C–H cleavage is mediated by **2 a**′ (Scheme 2). The radical cation **2 a′** is formed by a proton-coupled electron transfer from Me_2_S, as observed by EPR. BnNH_2_ promotes the formation of **2 a′** by mediating the de-aggregation and deprotonation of **2 a**′, thus generating the neutral semiquinone **2 a′′**, with subsequent H^•^ transfer, initiated by a charge-transfer event, from **1 a** to **2 a′′**. An α-amino radical is formed (**1 a′**), and it acts as a potent reductant,[27] thus reducing alloxan and forming **1 a′′**. Electron transfer from α-amino radicals to vicinal dicarbonyl compounds is regarded as one of the fastest reactions between a radical and a neutral closed-shell organic molecule.[28] Alloxan (**3 a**) reacts as an amide tautomer, thus allowing stabilization of a developing oxyanion character, a feature which is impossible for the inactive **3 b** (Table 1, entry 3). This captodative-stabilized radical[29] subsequently reacts with O_2_, thus generating **5**. The peroxyl radical **5** oxidizes **2 a′′′** to **2 a′**, thus forming the hydroperoxide **5′** and completing the catalytic cycle. Formation of stoichiometric DMSO is observed. Therefore Me_2_S mediates the reduction of **5′** to alloxan. Additionally, a purple by-product, consistent with the dye murexide (**6**; UV/vis *λ*_max_=521 nm; lit=520 nm),[30] is observed to accumulate from **3 a′′**, **3 a**, and ammonia This observation is consistent with a two-electron over-reduction of **3 a**, thus leading to catalyst deactivation and suggesting that **3 a′′** is not a catalytically active species.

**Scheme 2:**
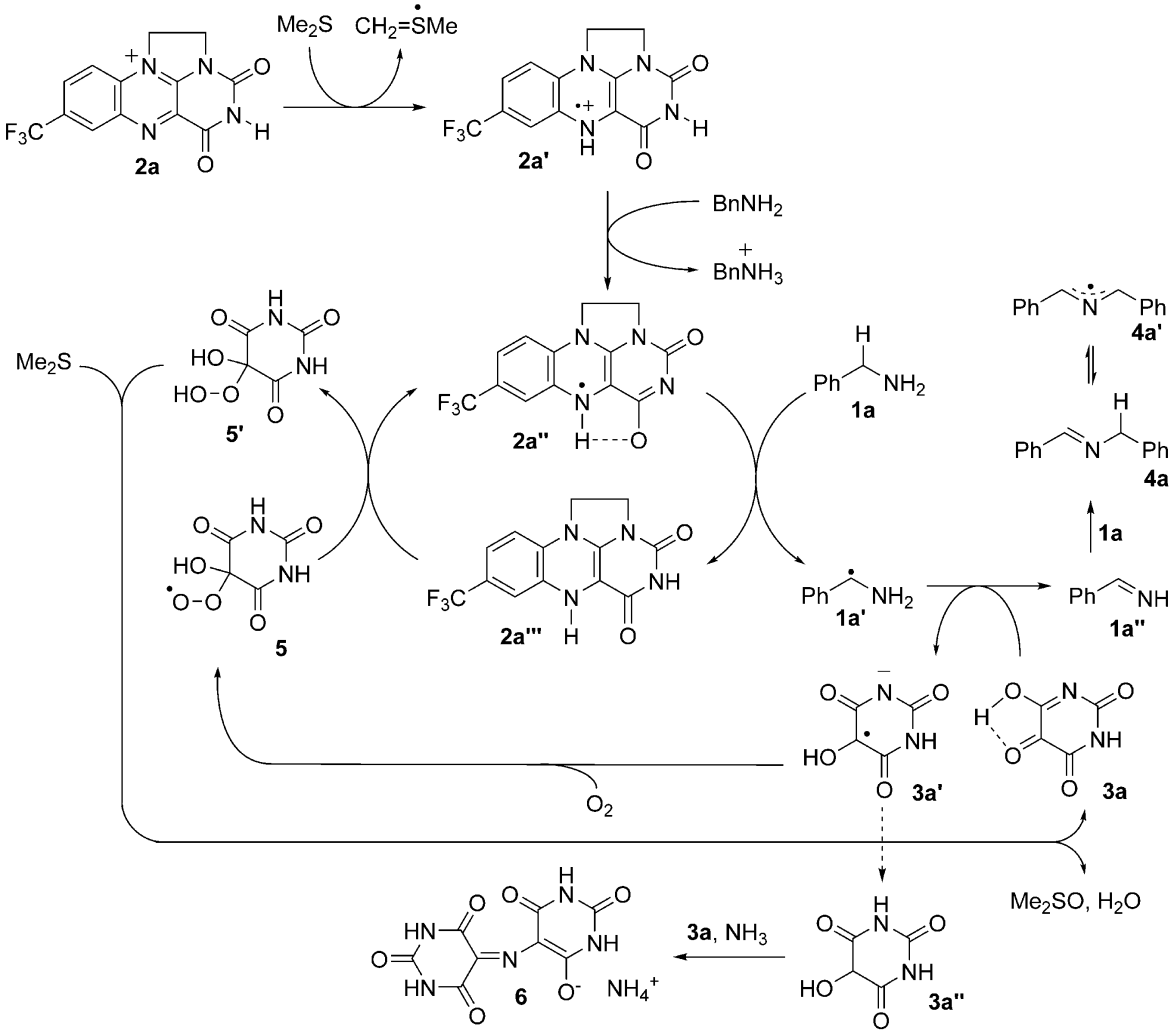
Proposed reaction mechanism showing amine oxidation mediated by the key flavin semiquinone 2 a′′.

This model study supports a homolytic C–H bond cleavage mediated by a flavin semiquinone, and with a substrate preference for benzylamines, it has prompted us to ask whether any reasonable insight into the enzymatic mechanism of MAO B can be achieved through consideration of this currently presented model system. A linear correlation exists between the substrate p*K*_a_ value and steady-state *k*_cat_ for MAO B (Figure 3),[31] and is consistent with a neutral amine substrate. It is significant that Hammett electronic correlations for MAO B are only apparent at pH 9.0. The similarity of the model′s KIE and Hammett profiles to the equivalent B isozyme data, when the enzyme kinetics are measured at pH 9.0, which is similar to this unbuffered system, is notable, (MAO B: *k*_H_/*k*_D_=2.25, *ρ*=−0.9 at pH 9.0).[32]

**Figure 3:**
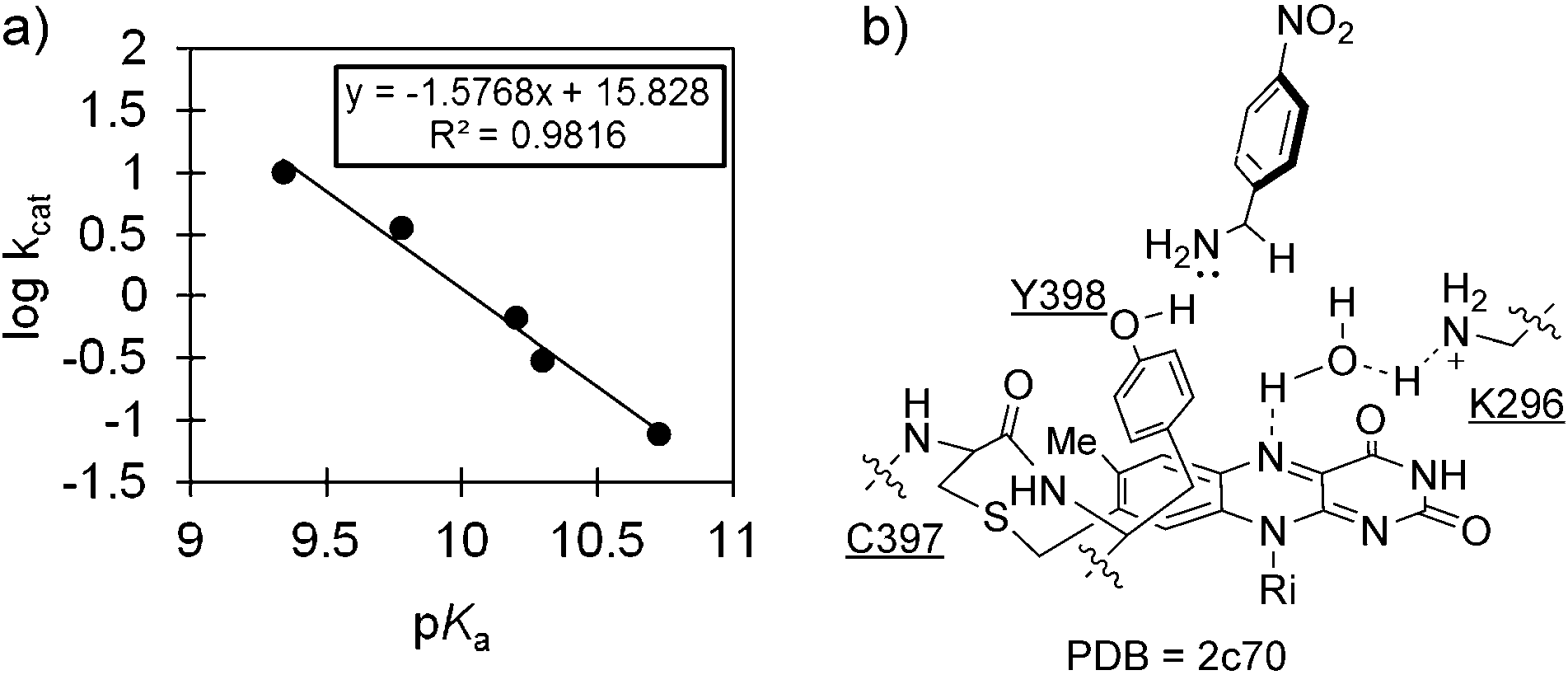
a) MAO B steady-state *k*_cat_ relationship to substrate p*K*_a_; relevant *k*_cat_ values from Ref. [31]. b) Crystal structure of MAO B with 4-nitrobenzylamine inhibitor.[33]

Our proposal for the MAO B mechanism is informed by the presented data, the substrate reactivity trends, and the pH sensitivity of MAO B.[34] This mechanistic suggestion centers upon a charge-transfer event promoted by the free-base substrate interacting with an electron-rich phenol of Y398 near the flavin acceptor, as demonstrated by Scrutton and co-workers.[35] This acceptor is itself activated by the H_2_O–K296 hydrogen-bonding motif. The neutral semiquinone thus formed can mediate hydrogen-atom transfer from the substrate, with the tyrosinyl radical cation now able to accept the second substrate electron, in direct analogy to the role played by alloxan in the currently discussed model. Indeed, both components can be viewed as redox-active hydroxylated units.

In summary, an aerobic, catalytic oxidation of benzylamines which mimics MAO B activity proceeding through charge-transfer-initiated substrate H^.^ abstraction has been developed. EPR spectroscopy has revealed the operation of an aerobically generated flavin semiquinone. KIE and Hammett studies have demonstrated a pH-dependent kinetic parallel to MAO B activity. This model system has opened up an additional mechanistic model of MAO B activity, that is, a charge-transfer event is harnessed to access a reactive neutral flavin semiquinone as the C–H abstracting species in MAO B.

## Supporting Information

As a service to our authors and readers, this journal provides supporting information supplied by the authors. Such materials are peer reviewed and may be re-organized for online delivery, but are not copy-edited or typeset. Technical support issues arising from supporting information (other than missing files) should be addressed to the authors.

miscellaneous_information
