# Supplementary material for: Catalytic Amine Oxidation under Ambient Aerobic Conditions: Mimicry of Monoamine Oxidase B
Source: Angew Chem Int Ed Engl. 2015 Jun 18;54(31):8997–9000. doi: 10.1002/anie.201503654 (PMC4524416; doi:10.1002/anie.201503654)
Supplement: Supplementary file 1 — miscellaneous_information [file anie0054-8997-sd1.pdf]

Angewandte Chemie
Eine Zeitschrift der Gesellschaft Deutscher Chemiker

Supporting Information

Catalytic Amine Oxidation under Ambient Aerobic Conditions: Mimicry of Monoamine Oxidase B**

*Alexander T. Murray, Myles J. H. Dowley, Fabienne Pradaux-Caggiano, Amgalanbaatar Baldansuren, Alistair J. Fielding, Floriana Tuna, Christopher H. Hendon, Aron Walsh, Guy C. Lloyd-Jones, Matthew P. John, and David R. Carbery**

anie_201503654_sm_miscellaneous_information.pdf

CONTENTS

**I**	**GENERAL INFORMATION**	**S2**
**II**	**REACTION OPTIMISATION**	**S3**
**III**	**SYNTHESIS OF COMPOUNDS**	**S4**
**IV**	**HPLC REACTION MONITORING**	**S15**
**V**	**ELECTRON PARAMAGNETIC RESONANCE SPECTROSCOPY**	**S24**
**VI**	**COMPUTATIONAL AND THEORETICAL DETAILS**	**S30**
**IV**	**NMR SPECTRA**	**S31**
**V**	**REFERENCES**	**S63**

I GENERAL INFORMATION

All reagents were purchased from commercial suppliers: Acros Organics, Alfa Aesar, Sigma Aldrich or Fluorochem and used without further purification. Flash chromatography was performed on chromatography grade, silica, 60 Å particle size 35-70 micron from Sigma Aldrich using the solvent system as stated. ^{1}H and ^{13}C NMR was performed on Brüker Avance 250 (^{1}H 250 MHz) Brüker Avance 300 (^{1}H 300 MHz and ^{13}C 75 MHz), Brüker Avance 400 (^{1}H 400 MHz and ^{13}C 100 MHz), Brüker Avance 500 (^{1}H 500 MHz and ^{13}C 125 MHz) or Agilent ProPulse 500 (^{1}H 500 MHz and ^{13}C 125 MHz) as stated. Chemical shifts are reported in parts per million (ppm) relative to tetramethylsilane (TMS) (δ = 0.00). Coupling constants are reported in Hertz (Hz) and signal multiplicity is denoted as singlet (s), doublet (d), triplet (t), quartet (q), quintet (quin.), sextet (sex.), septet (sept.), multiplet (m), and broad (br). High resolution mass spectrometry electrospray (ESI) was performed on a Brüker μTOF using electrospray ionisation (ESI) in either positive or negative ionisation. Infra-red spectroscopy was carried out using a Perkin Elmer Spectrum RX FT-IR system. HPLC data was recorded on an Agilent 1260 Infinity system using an Agilent Eclipse XDB-CN 5 µm, 4.6 x 150 mm cyano column. EPR spectroscopy was performed on a Brüker EMX Micro X-band with 1.0 T electromagnet or for pulse X-band experiments a Brüker Elexsys E580 with 1.8 T electromagnet.

Flavins were synthesised by the previously published procedures[1], with the modification that for 7-CF_3 substituted flavin **2a** recrystallization was performed with hot filtration from CF_3CH_2OH instead of HCO_2H in order to ensure complete removal of alloxan monohydrate (**3a**).

II REACTION OPTIMISATION

Table 1. Optimisation of the reaction conditions

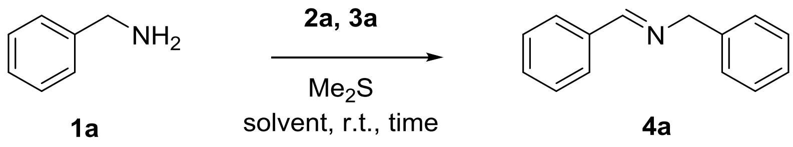

Entry	3 (mol%)	6 (mol%)[a]	Me_2S (equiv)	Solvent	Time (h)	Conv %[b]
1	9	1	1.5	H_2O	3	<5
2	9	1	1.5	CH_2Cl_2	3	<5
3	9	1	1.5	PhMe	3	<5
4	9	1	1.5	MeOH	3	24
5	9	1	1.5	MeCN	3	22
6	9	1	1.5	HFIP	3	46
7	9	1	1.5	TFE	3	56
8	9	1	1.5	TFE	3	<5[c]
9	9	1	1.5	TFE	3	49[d]
10	9	1	0	TFE	3	26
11	7	0.7	6	TFE	18	60
12	7	1	10	TFE	1	100
13	7	0	10	TFE	1	<5
14	0	1	10	TFE	1	<5
15	2[e]	2[e]	10	TFE	5	94[f]

[a]Relative to benzylamine. [b]Assayed against relevant ^1H NMR signals.[c]Under N_2. [d]Dark (wrapped in foil) [e]Added as two portions separated by 2 h. [f]Isolated yield.

III Synthesis of compounds

General Procedure for flavinium/alloxan-catalysed oxidative dimerisation of aromatic amines (General Procedure A)

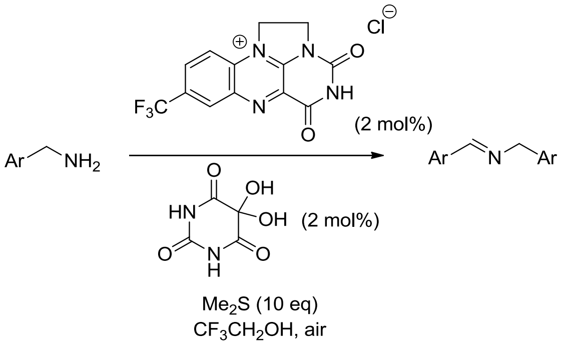

Dimethyl sulfide (0.37 mL, 5 mmol) was added to a mixture of flavin catalyst **2a** (1.7 mg, 5 μmol, 1 mol%) and alloxan monohydrate **3a** (0.8 mg, 5 μmol, 1 mol%) in 2,2,2-trifluoroethanol (1 mL). The solution was stirred under air for 10 minutes, then amine (0.5 mmol) was added dropwise by syringe and stirred under air for 2 h, after which time a further 1 mol% **x** and 1 mol% **y** were added. After reaction was complete, the solvent was removed *in vacuo*, and the crude imine was purified by washing through a small pad of base-washed silica (1:2 petrol:EtOAc + 2% Et_3N).

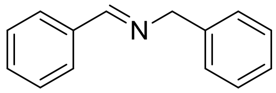

N-benzylidene-1-phenylmethanamine (4a)

Following General Procedure A using benzylamine **1a** (55 µL) for 5 h gave N-benzylidene-1-phenylmethanamine **4a** as an oil (46 mg, 94%).

^{1}H NMR (300 MHz, $CDCl_3$) δ_H 8.41 (s, 1H), 7.84 – 7.76 (m, 2H), 7.49 – 7.21 (m, 8H), 4.84 (s, 2H).

^{13}C NMR (75 MHz, $CDCl_3$) δ_C 162.1, 139.4, 136.3, 130.9, 128.7, 128.6, 128.4, 128.1, 127.1, 65.2. Data in accordance with that previously published.[2]

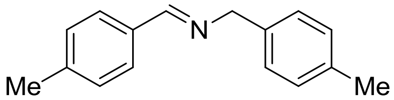

N-(4-methylbenzylidene)-1-(p-tolyl)methanamine (4b)

Following General Procedure A using 4-methylbenzylamine **1b** (64 µL) for 5 h gave N-(4-methylbenzylidene)-1-(p-tolyl)methanamine **4b** as a white solid (55 mg, 99%).

^{1}H NMR (300 MHz, $CDCl_3$) δ_H 8.24 (s, 1H), 7.57 (d, 2H, J=8.1), 7.16-7.01 (m, 6H), 4.67 (s, 2H), 2.28 (s, 3H), 2.24 (s, 3H)

^{13}C NMR (75 MHz, $CDCl_3$) δ_C 161.8, 141.1, 136.6, 136.4, 133.7, 129.4, 129.2, 128.3, 128.0, 64.9, 21.6, 21.2.

Data in accordance with that previously published.[2]

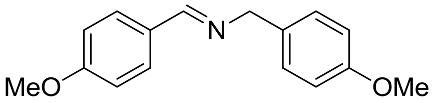

N-(4-methoxybenzylidene)-1-(4-methoxyphenyl)methanamine (4c)

Following General Procedure A using 4-methoxybenzylamine **1c** (65 µL) for 5 h gave N-(4-methoxybenzylidene)-1-(4-methoxyphenyl)methanamine **4c** as an oil (61 mg, 95%).

^{1}H NMR (300 MHz, $CDCl_3$) δ_H 8.20 (s, 1H), 7.62 (d, 2H, J=8.6), 7.16 (d, 2H, J=8.7), 6.86-6.75 (m, 4H), 4.64 (s, 2H), 3.74 (s, 3H), 3.70 (s, 3H).

^{13}C NMR (75 MHz, $CDCl_3$) δ_C 161.7, 161.0, 158.7, 131.7, 129.9, 129.2, 129.2, 114.0, 113.9, 64.5, 55.4, 55.3.

Data in accordance with that previously published.[2]

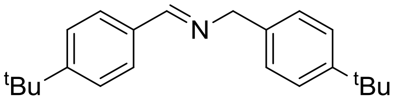

N-(4-(tert-butyl)benzylidene)-1-(4-(tert-butyl)phenyl)methanamine (4d)

Following General Procedure A using (4-(tert-butyl)phenyl)methanamine **1d** (88 µL) for 5 h gave N-(4-(tert-butyl)benzylidene)-1-(4-(tert-butyl)phenyl)methanamine **4d** as an oil (71 mg, 92%).

^{1}H NMR (300 MHz, $CDCl_3$) δ_H 8.29(s, 1H), 7.64 (d, 2H, J=8.7), 7.38 – 7.15 (m, 6H), 4.70 (s, 2H), 1.25 (s, 9H), 1.23 (s, 9H).

^{13}C NMR (75 MHz, $CDCl_3$) δ_C 161.8, 154.2, 149.9, 136.6, 133.7, 128.2, 127.8, 125.7, 125.5, 65.0, 35.0, 34.6, 31.5, 31.4.

Data in accordance with that previously published.[2]

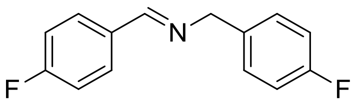

N-(4-fluorobenzylidene)-1-(4-fluorophenyl)methanamine (4e)

Following General Procedure A using 4-fluorobenzylamine **1e** (57 µL) for 18 h gave N-(4-chlorobenzylidene)-1-(4-chlorophenyl)methanamine **4e** as an oil (57 mg, 98%).

^{1}H NMR (300 MHz, $CDCl_3$) δ_H 8.26 (s, 1H), 7.73 - 7.64 (m, 2H), 7.26 - 7.15 (m, 2H), 7.07 - 6.88 (m, 4H), 4.68 (s, 2H).

^{13}C NMR (75 MHz, $CDCl_3$) δ_C 166.2, 163.7, 162.8, 160.7, 160.5, 135.0 (d, $J_{C,F}$ = 3.2 Hz), 132.4 (d, $J_{C,F}$ = 3.2 Hz), 130.3 (d, $J_{C,F}$ = 8.8 Hz), 129.6 (d, $J_{C,F}$ = 8.8 Hz), 115.8 (d, $J_{C,F}$ = 22.2 Hz), 115.4 (d, $J_{C,F}$ = 22.2 Hz), 64.3.

Data in accordance with that previously published.[2]

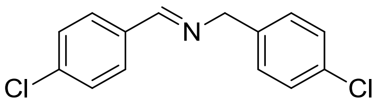

N-(4-chlorobenzylidene)-1-(4-chlorophenyl)methanamine (4f)

Following General Procedure A using 4-chlorobenzylamine **1f** (61 µL) for 18 h gave N-(4-chlorobenzylidene)-1-(4-chlorophenyl)methanamine **4f** as a white powder (51 mg, 77%).

^{1}H NMR (300 MHz, $CDCl_3$) δ_H 8.27 (s, 1H), 7.64 (d, 2H, J=8.6), 7.31 (d, 2H, J=8.5), 7.27 - 7.16 (m, 4H), 4.69 (s, 2H).

^{13}C NMR (75 MHz, $CDCl_3$) δ_C 161.0, 137.7, 137.0, 134.5, 132.9, 129.6, 129.4, 129.1, 128.8, 64.3.

Data in accordance with that previously published.[2]

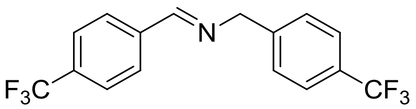

N-(4-(trifluoromethyl)benzylidene)-1-(4-(trifluoromethyl)phenyl)methanamine (4g)

Following General Procedure A using 4-(trifluoromethyl)benzylamine **1g** (71 µL) for 18 h with 4% alloxan and 4% flavin gave N-(4-(trifluoromethyl)benzylidene)-1-(4-(trifluoromethyl)phenyl)methanamine **4g** as an oil (31 mg, 37%).

^{1}H NMR (300 MHz, $CDCl_3$) δ_H 8.47 (s, 1H), 7.91 (d, 2H, J=8.1 Hz), 7.74 – 7.55 (m, 4h), 7.47 (d, 2H, J=8.1 Hz) 4.90 (s, 2H).

^{13}C NMR (75 MHz, $CDCl_3$) δ_C 161.3, 143.0, 139.0, 132.9, 132.5, 128.7, 128.6, 125.8 (q, JC,F = 3.9 Hz), 125.6 (q, JC,F = 3.9 Hz), 122.5, 64.6.

Data in accordance with that previously published.[2]

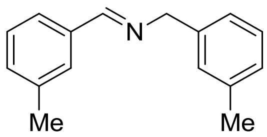

N-(3-methylbenzylidene)-1-(m-tolyl)methanamine (4h)

Following General Procedure A using 3-methylbenzylamine **1h** (63 µL) for 5 h gave N-(3-methylbenzylidene)-1-(m-tolyl)methanamine **4h** as an oil (51 mg, 91%).

^{1}H NMR (300 MHz, $CDCl_3$) δ_H 8.26 (s, 1H), 7.56 (s, 1H), 7.50 – 7.40 (m, 1H), 7.26 – 7.08 (m, 3H), 7.08 – 6.94 (m, 3H), 4.69 (s, 2H), 2.28 (s, 3H), 2.25 (s, 3H),

^{13}C NMR (75 MHz, $CDCl_3$) δ_C 162.2, 139.2, 138.4, 138.2, 136.2, 131.7, 128.8, 128.5, 128.5, 127.8, 126.0, 125.2, 65.2.

Data in accordance with that previously published.[3]

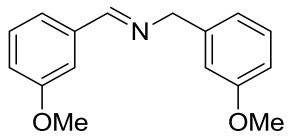

N-(3-methoxybenzylidene)-1-(3-methoxyphenyl)methanamine (4h)

Following General Procedure A using 3-methoxybenzylamine **1h** (64 µL) for 5 h gave N-(3-methoxybenzylidene)-1-(3-methoxyphenyl)methanamine **4h** as an oil (46 mg, 72%).

^{1}H NMR (400 MHz, $CDCl_3$) δ_H 8.4 (s, 1H), 7.49 – 7.43 (m, 1H), 7.42 – 7.28 (m, 3H), 7.10 – 6.83 (m, 4H), 4.86 (s, 2H), 3.90 (s, 3H), 3.87 (s, 3H).

^{13}C NMR (100 MHz, $CDCl_3$) δ_C 162.2, 160.0, 159.9, 140.9, 137.7, 129.7, 129.6, 121.8, 120.4, 117.7, 113.8, 112.6, 111.8, 65.0, 55.5, 55.3.

Data in accordance with that previously published.[3]

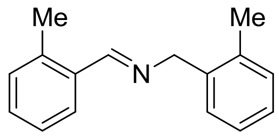

N-(2-methylbenzylidene)-1-(o-tolyl)methanamine (4j)

Following General Procedure A using 2-methylbenzylamine **1j** (62 µL) for 5 h gave N-(3-methoxybenzylidene)-1-(3-methoxyphenyl)methanamine **4j** as an oil (53 mg, 96%).

^{1}H NMR (300 MHz, $CDCl_3$) δ_H 8.58 (s, 1H), 7.88 – 7.81 (m, 1H), 7.26 – 7.06 (m, 7H), 4.74 (s, 2H), 2.42 (s, 3H), 2.31 (s, 3H).

^{13}C NMR (75 MHz, $CDCl_3$) δ_C 160.7, 137.8, 137.7, 136.2, 134.3, 130.9, 130.4, 130.2, 128.4, 127.8, 127.1, 126.3, 126.2, 63.4, 19.5, 19.4.

Data in accordance with that previously published.[2]

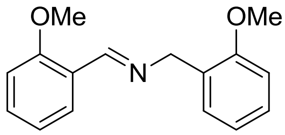

N-(2-methoxybenzylidene)-1-(2-methoxyphenyl)methanamine (4k)

Following General Procedure A using 2-methoxybenzylamine **1k** (65 µL) for 5 h gave N-(2-methoxybenzylidene)-1-(2-methoxyphenyl)methanamine **4k** as an oil (43 mg, 68%).

^{1}H NMR (300 MHz, $CDCl_3$) δ_H 8.76 (s, 1H), 7.95 (dd, J = 7.7, 1.8 Hz, 1H), 7.36 – 7.07 (m, 3H), 6.99 – 6.72 (m, 4H), 4.75 (s, 2H), 3.77 (s, 3H), 3.75 (s, 3H).

^{13}C NMR (75 MHz, $CDCl_3$) δ_C 158.9, 158.4, 157.1, 131.9, 129.2, 128.2, 128.0, 127.6, 124.9, 120.8, 120.6, 111.1, 110.2, 59.8, 55.6, 55.4.

Data in accordance with that previously published.[3]

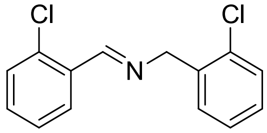

N-(2-chlorobenzylidene)-1-(2-chlorophenyl)methanamine (4l)

Following General Procedure A using 2-chlorobenzylamine **1l** (60 µL) for 18 h gave N-(2-chlorobenzylidene)-1-(2-chlorophenyl)methanamine **4l** as a white powder (44 mg, 66%).

^{1}H NMR (300 MHz, $CDCl_3$) δ_H 8.79 (s, 1H), 8.06-8.00 (m, 2H) 7.37-7.08 (m, 6H), 4.86 (s, 2H).

^{13}C NMR (75 MHz, $CDCl_3$) δ_c 159.9, 136.9, 135.3 133.5, 133.2, 131.8, 129.9, 129.8, 129.4, 128.6, 128.4, 127.1, 127.0, 62.3.

Data in accordance with that previously published.[3]

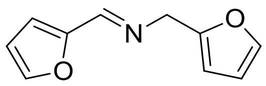

1-(furan-2-yl)-N-(furan-2-ylmethylene)methanamine (4m)

Following General Procedure A using furfurylamine **1m** (44 µL) for 18 h gave N-(2-chlorobenzylidene)-1-(2-chlorophenyl)methanamine **4m** as an oil (31 mg, 70%).

^{1}H NMR (300 MHz, $CDCl_3$) δ_H 8.12 (s, 1H), 7.52 (d, 1H, J = 1.6), 7.38 (q, 1H, J = 1.0), 6.78 (d, 1H, J = 3.4), 6.47 (q, 1H, J = 1.7), 6.34 (q, 1H, J = 1.3), 6.28 (dd, 1H, J_1 = 0.6, J_2 = 3.2), 4.75 (s, 2H);

^{13}C NMR (75 MHz, $CDCl_3$) δ_C 151.8, 151.34, 151.2, 145.0, 142.3, 114.6, 111.7, 110.4, 107.9, 56.9.

Data in accordance with that previously published.[2]

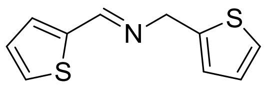

1-(thiophen-2-yl)-N-(thiophen-2-ylmethylene)methanamine (4n)

Following General Procedure A using 2-thiophenemethylamine **1n** (51 µL) for 5 h gave 1-(thiophen-2-yl)-N-(thiophen-2-ylmethylene)methanamine **4n** as an oil (45 mg, 87%).

^{1}H NMR (300 MHz, $CDCl_3$) δ_H 8.42 (s, 1H), 7.42 (dt, *J* = 4.8, 1.0 Hz, 1H), 7.33 (dd, *J* = 3.6, 1.0 Hz, 1H), 4.8, 1.6 Hz, 1H), 7.07 (dd, *J* = 5.0, 3.6 Hz, 1H), 7.03 – 6.95 (m, 2H), 4.95 (s, 1H).

^{13}C NMR (75 Hz, $CDCl_3$) δ_C 155.5, 142.2, 141.6, 131.1, 129.5, 127.5, 127.7, 125.4, 125.0, 58.6.

Data in accordance with that previously published.[4]

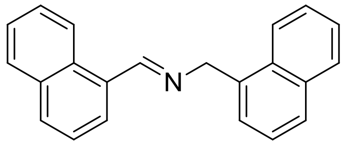

1-(naphthalen-1-yl)-N-(naphthalen-1-ylmethylene)methanamine (4o)

Following General Procedure A using 1-napthylmethylamine **1o** (73 µL) for 5 h gave 1-(naphthalen-1-yl)-N-(naphthalen-1-ylmethylene)methanamine **4o** as an oil (53 mg, 72%).

^{1}H NMR (300 MHz, $CDCl_3$) δ_H 9.09 (s, 1H), 8.96 (d, *J* = 8.1 Hz, 1H), 8.26 (d, *J* = 8.1 Hz, 1H), 8.00 – 7.77 (m, 5H), 7.63 – 7.44 (m, 7H), 5.42 (s, 2H).

^{13}C NMR (75 MHz, $CDCl_3$) δ_C 162.1, 135.6, 134.0, 133.9, 131.8, 1331.7, 131.4, 131.3, 129.3, 128.8, 128.7, 127.9, 127., 126.3, 126.2, 126.0, 125.8, 125.8, 125.4, 124.5, 124.1, 63.4.

Data in accordance with that previously published.[2]

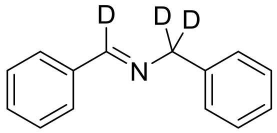

Benzenemethan-*d*$_2$-amine, N-(phenylmethylene-*d*) (8)

Following General Procedure A using 2,2-dideuterobenzylamine **7** (56 µL) for 5 h gave Benzenemethan-*d*$_2$-amine, N-(phenylmethylene-*d*) **8** as an oil (25 mg, 50%).

^{1}H NMR (500 MHz, $CDCl_3$) δ_H 7.86 – 7.77 (m, 2H), 7.49 – 7.24 (m, 8H).

^{13}C NMR (125 MHz, $CDCl_3$) δ_C 161.6 (t, $J_{C,D}$ = 23 Hz), 139.3, 126.3, 130.8, 128.7, 128.6, 128.3, 128.1, 127.1, 64.4 (p, $J_{C,D}$ = 20 Hz).

IR ν_{max} (neat) 3059, 3026, 2137, 1627, 1448 cm^{-1}.

HRMS (ESI, +ve) *m/z* calcd. for $C_{14}H_{10}D_3N$ 199.1315, found: 199.1341 $(M+H)^{+}$.

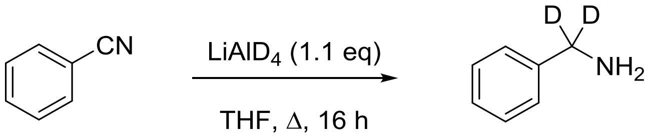

2,2-dideuterobenzylamine (7)

According to the published procedure,[5] 1M THF solution of lithium aluminium deuteride (9.4 mL, 9.4 mmol) was diluted with dry THF (5 mL) and cooled to 0 ˚C . Benzonitrile (0.88 mL, 8.5 mmol) was added dropwise as a solution in dry THF (5 mL). The solution was warmed to r.t. then refluxed for 16 h. The reaction was cooled in ice, and quenched sequentially with Et_2O (5 mL), H_2O (0.5 mL) and 10% NaOH (0.7 mL). The mixture was filtered through celite and water (5 mL) was added and the mixture was extracted with ether (3 x 10 mL). The solution was dried with $MgSO_4$ and solvent was removed *in vacuo*. The oil was purified by Kugelrohr distillation (2 mmHg, 37 ˚C) to give 2,2-dideuterobenzylamine **7** as a clear oil (480 mg, 52%)

^{1}H NMR (300 MHz, $CDCl_3$) δ_H 7.45 – 7.2 (m, 5H), 1.88 – 1.56 (bs, 2H).

^{13}C NMR (75 MHz, $CDCl_3$) δ_c 143.3, 128.6, 127.2, 126.9, 45.9.

IR (neat) ν_{max} 2924, 2854, 2796, 1643, 1467, 1117 cm^{-1}.

HRMS (ESI, +ve) *m/z* calcd. for $C_7H_5D_2$ 93.0668, found: 93.0677 $(M-NH_3)^+$.

Data in accordance with that previously published.[6]

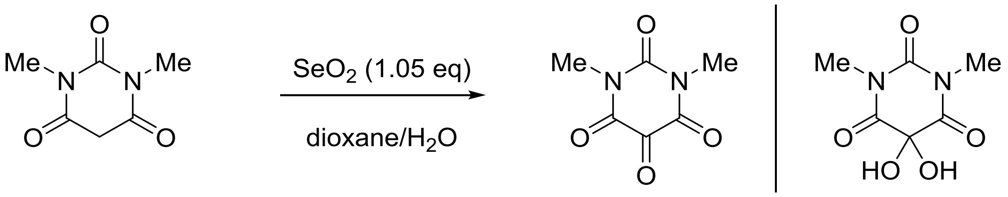

***N*,*N*-dimethylalloxan (3b)**

In a modification of the published procedure,[7] to a solution of selenium dioxide (0.75 g, 6.8 mmol) in dioxane (3 mL) and water (180 µL) was added *N,N*-dimethylbarbituric acid (1.06 g, 6.4 mmol) as a suspension in dioxane (5 mL) using an addition funnel. The mixture was refluxed for 72 h, then cooled and filtered through celite, solvent was removed *in vacuo*, and the crude product was recrystallized from benzene/hexane to give N,N-dimethylalloxan **3b** as a white powder (226 mg, 21%), and was observed in DMSO as a 2:1 ratio of its monohydrate to the free carbonyl.

^{1}H NMR (300 MHz, d$_6$-DMSO) δ_H hydrate: 7.76 (s, 2H), 3.13 (s, 6H), carbonyl: 3.21 (s, 3H).
^{13}C NMR (75 MHz, d$_6$-DMSO) δ_C 168.1, 164.5, 156.3, 150.8, 150.6, 85.5, 28.4.
IR ν_{max} (neat) 3364, 1679, 1445, 1368, 1106 cm^{-1}.
HRMS (ESI, +ve) *m/z* calcd. for $C_6H_8N_2O_5Na$ 211.0314, found: 211.0331 (M+Na)$^+$
M.P. = 250-255 °C (lit = 254-5 °C).
Data in accordance with that previously published.[8]

IV HPLC Reaction Monitoring

General Procedure for kinetic monitoring of imine production (General Procedure B)

Dimethyl sulfide was added to a mixture of flavin catalyst **2a**, alloxan monohydrate **3a** and naphthalene (5.1 mg, 40 μmol) in 2,2,2-trifluoroethanol (1 mL). The solution was stirred under air for 10 minutes, then amine was added dropwise by syringe and stirred under air with aliquot samples of ca. 5 μL taken at specified intervals, diluted into ca. 20 μL MeOH in an HPLC vial which was then filled to 1.5 mL with MeCN and analysed by HPLC (UV detection) with naphthalene acting as an internal standard. Standard conditions unless stated to be deviated from were 0.5 M amine, 0.01 M **2a** and **3a**, and 5 M Me_2S.

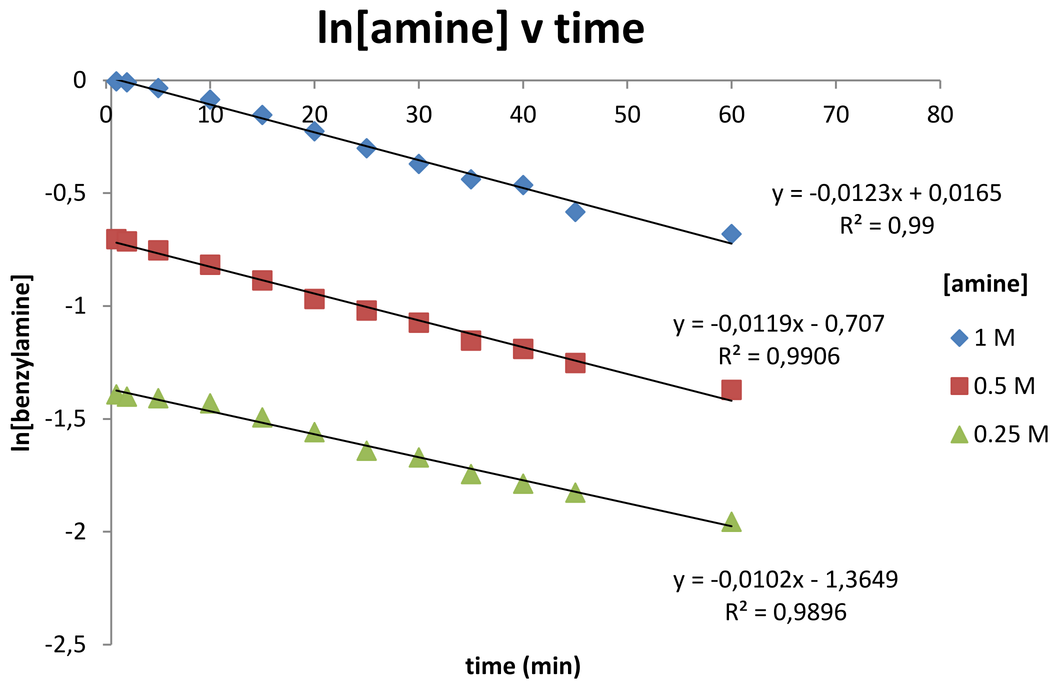

Figure S1. Linear plot of ln[**1a**] against time determined by HPLC; linearity suggests first order kinetics in amine.

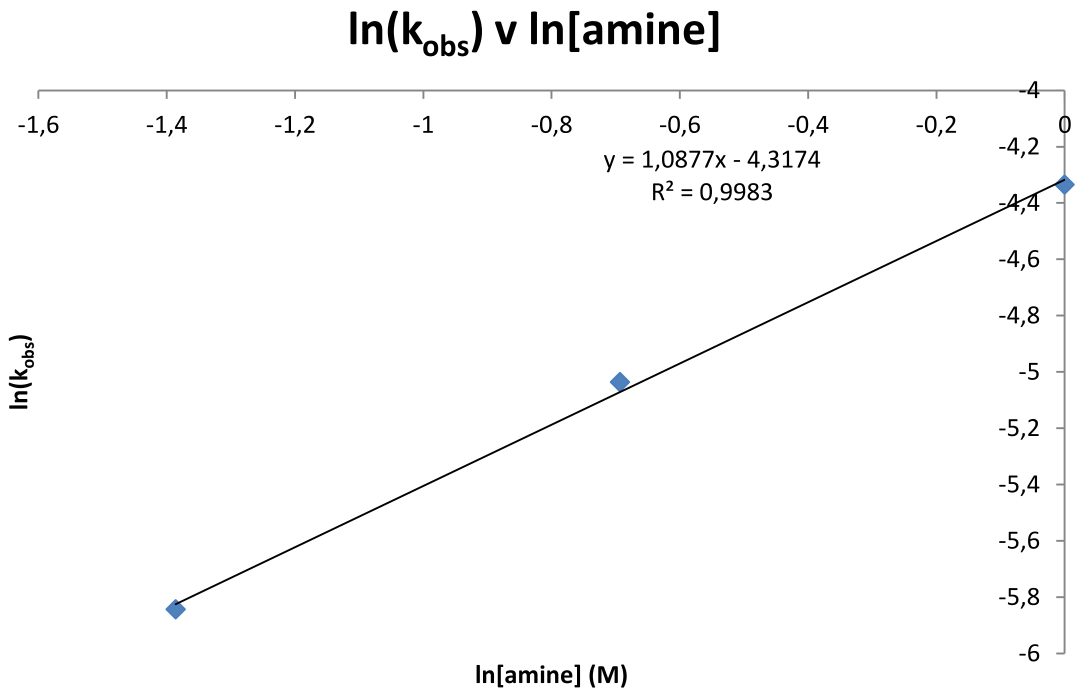

Figure S2. Log – log plot showing natural logarithm of observed rate (k_{obs}) vs. natural logarithm of amine concentration determined from data in figure **S1**. Slope close to m=1 is further evidence for first order kinetics.

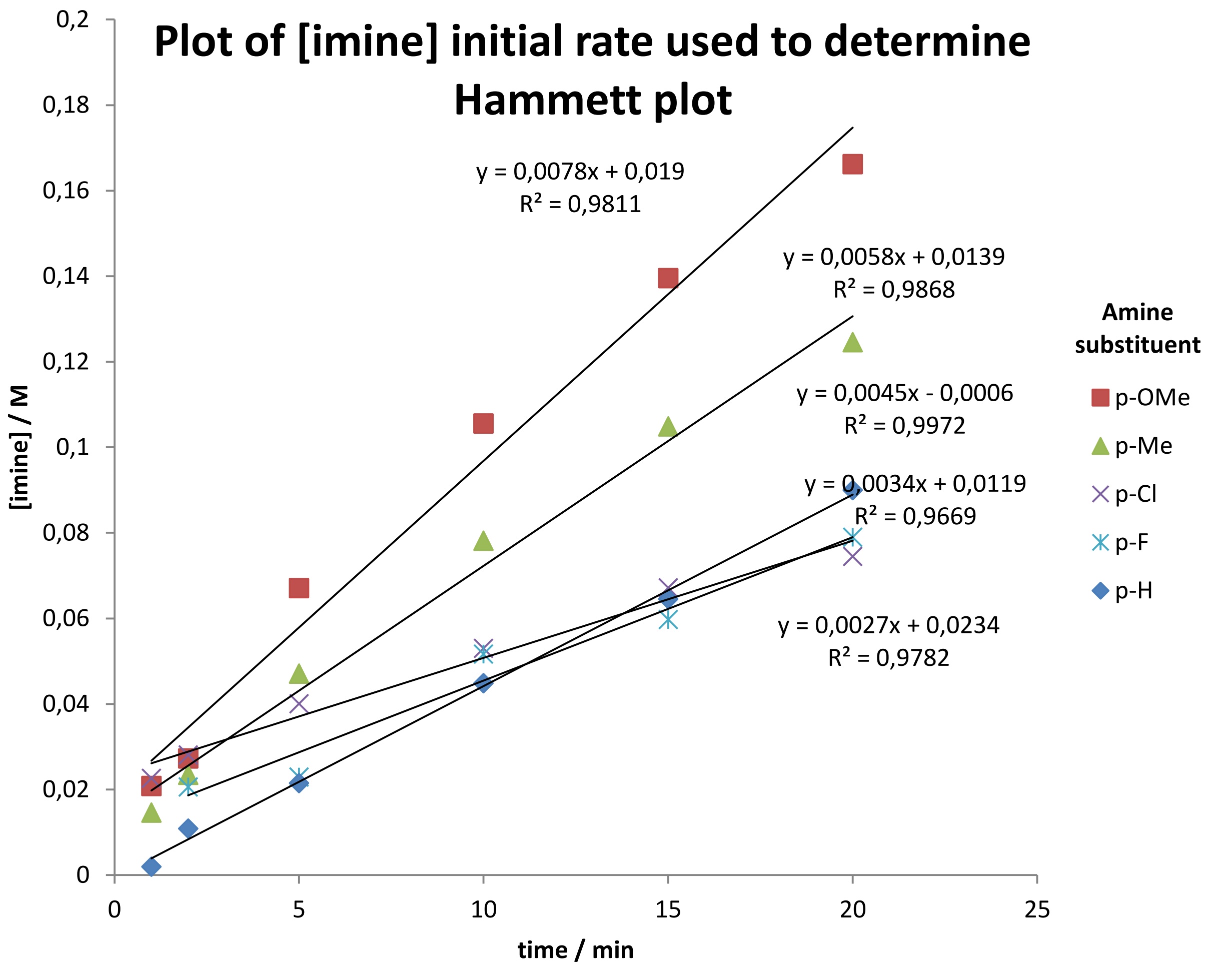

Figure S3. Plot of imine formation for various *p*-substituted amines, initial rate method used, determined by HPLC. See figure **2** in the manuscript for the Hammett correlations.

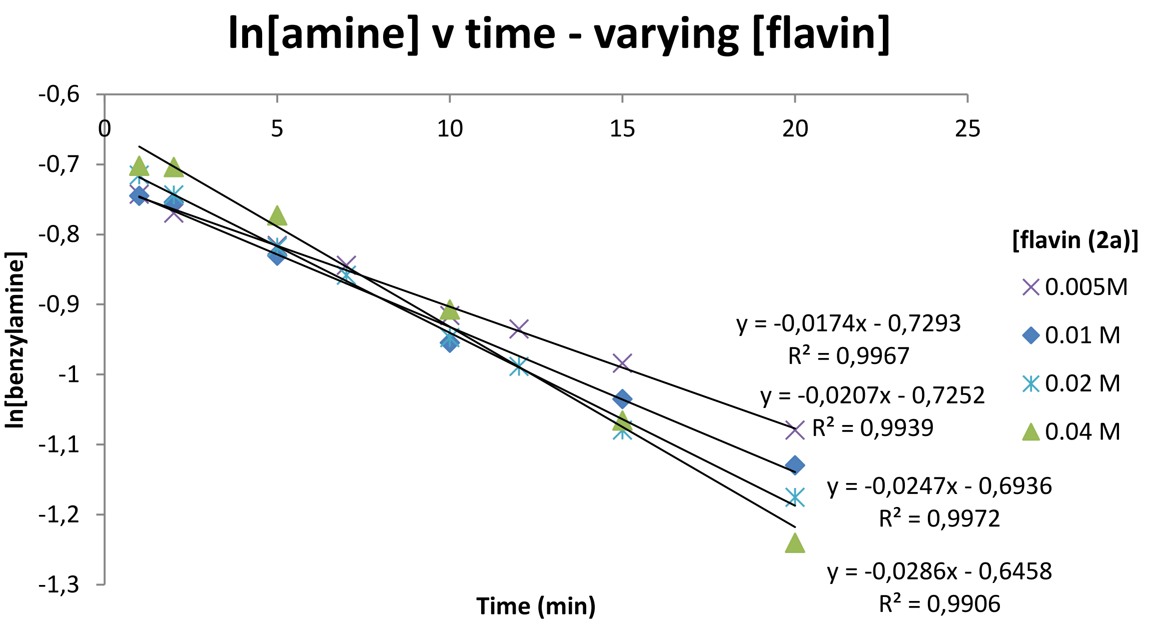

Figure S4. Plot of ln[**1a**]; varying concentration of flavin **2a**. See figure **S6** for details of results.

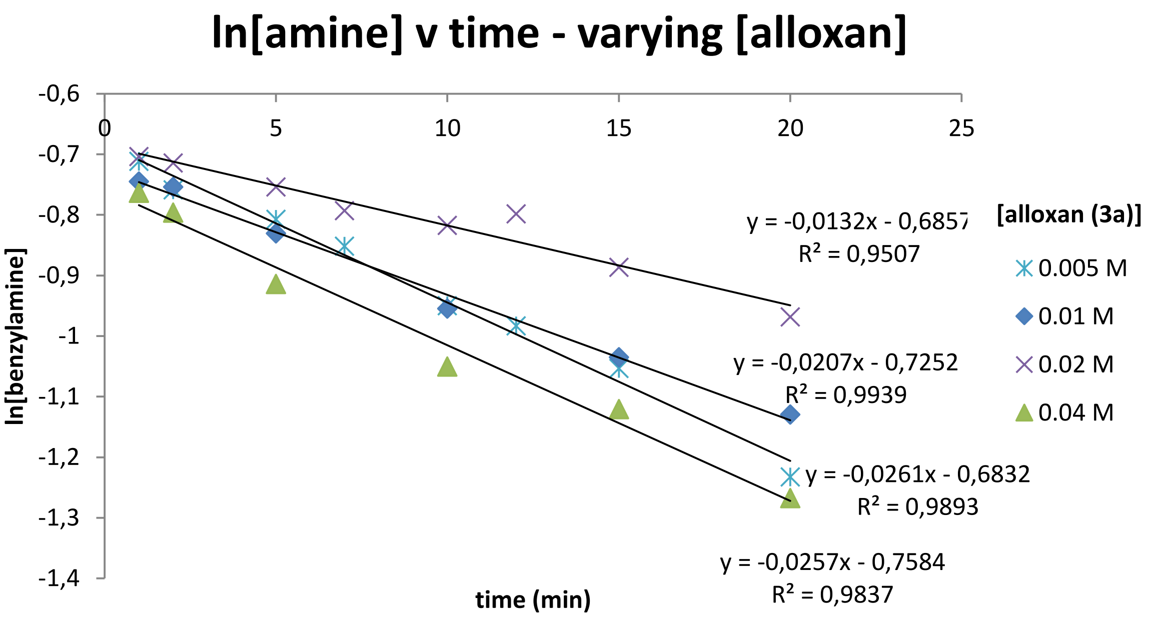

Figure S5. Plot of ln[**1a**]; varying concentration of alloxan **3a**. See figure **S6** for details of results.

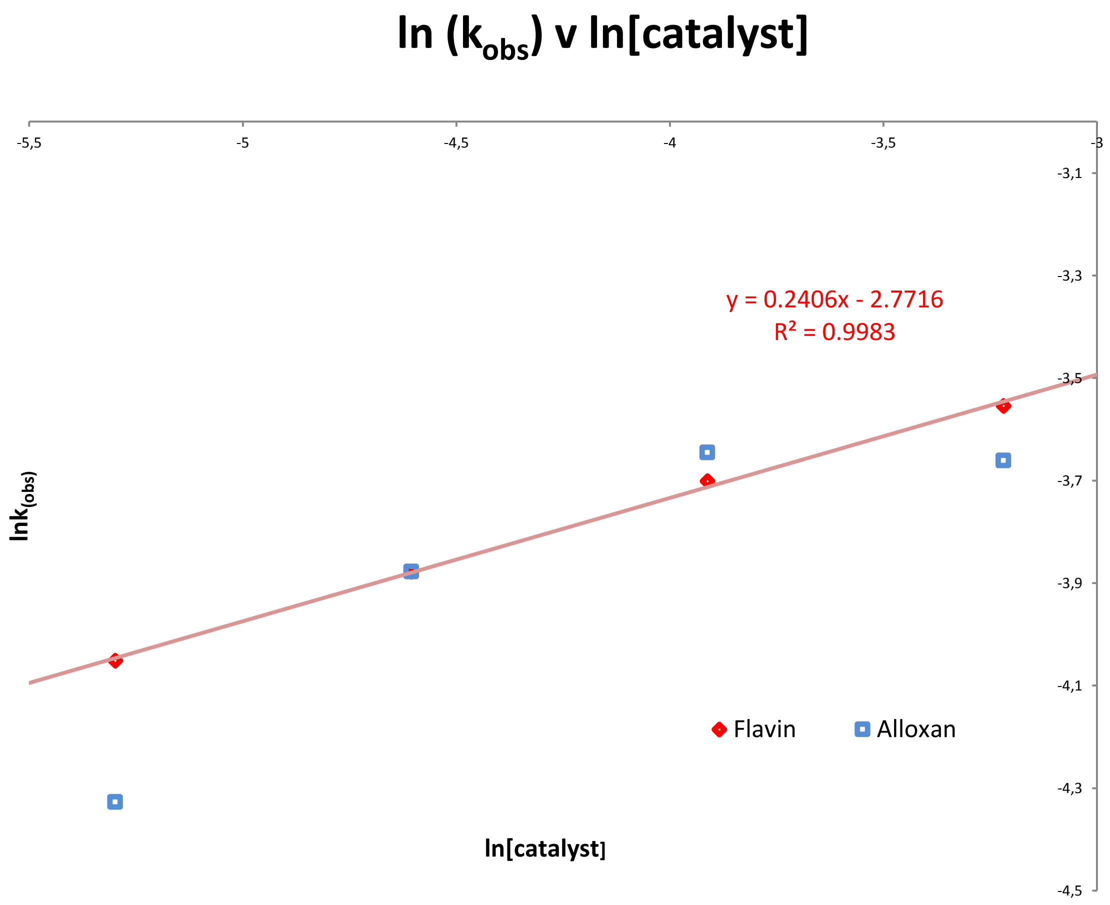

Figure S6. Plot of ln(k_{obs}) against ln[**2a**] and ln[**3a**], showing fractional order in **2a** and non-linear, saturating-like behaviour in **3a**.

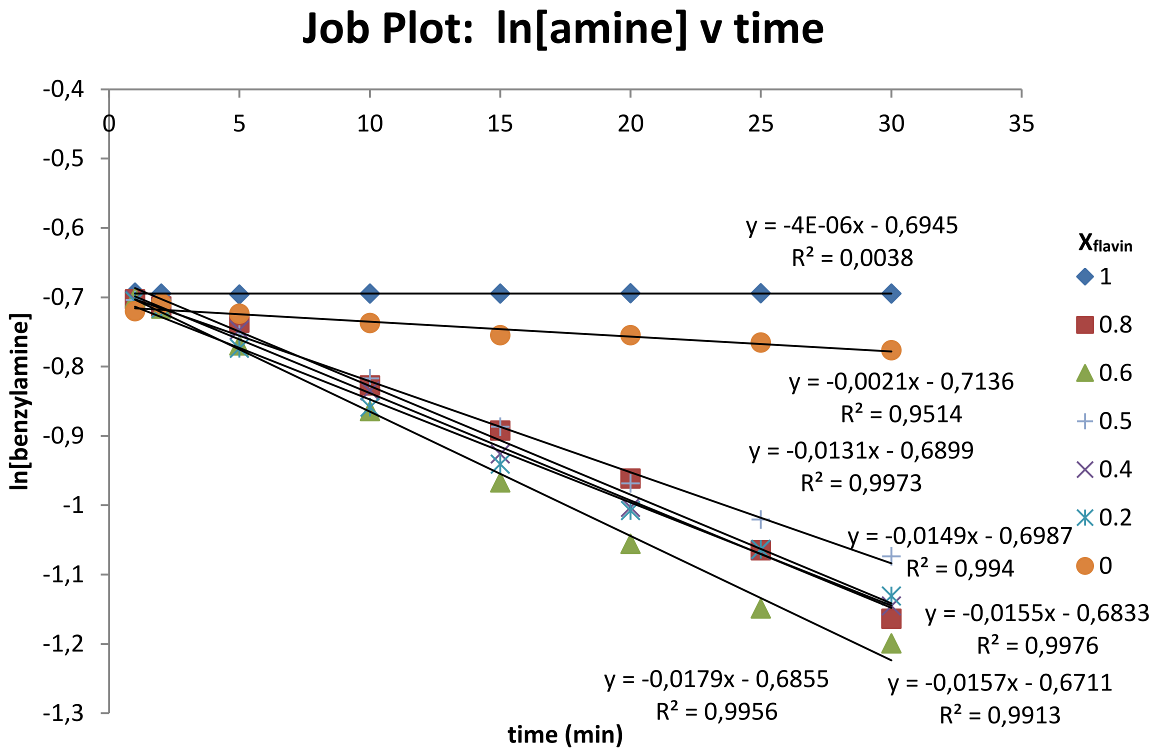

Figure S7. Job plot showing mole fraction (χ) flavin such that the total concentration [**2a**+**3a**] (flavin + alloxan) = 0.04 M. See figure **S8** for job plot treatment.

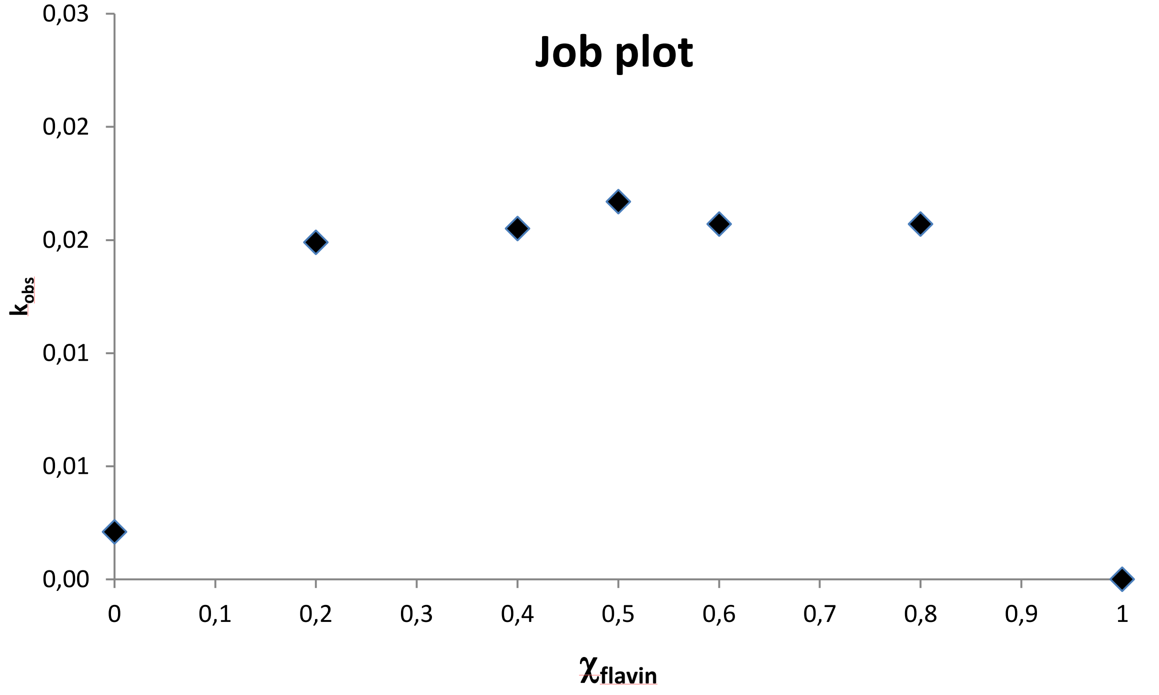

Figure S8. Job Plot showing results from **S7**. (k_{obs} vs. χ_{flavin})

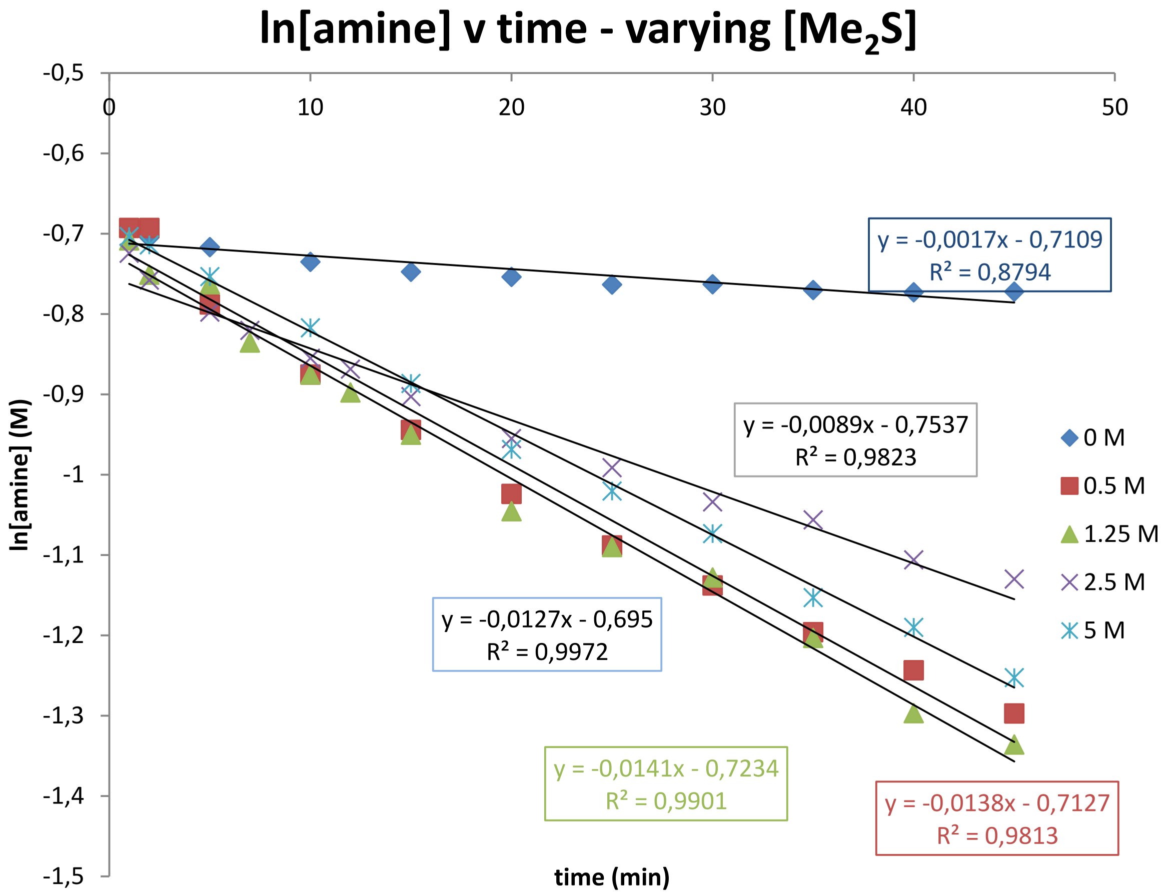

Figure S9. Plot of ln[**1a**]; varying concentration of Me_2S. See figure **S10** for details.

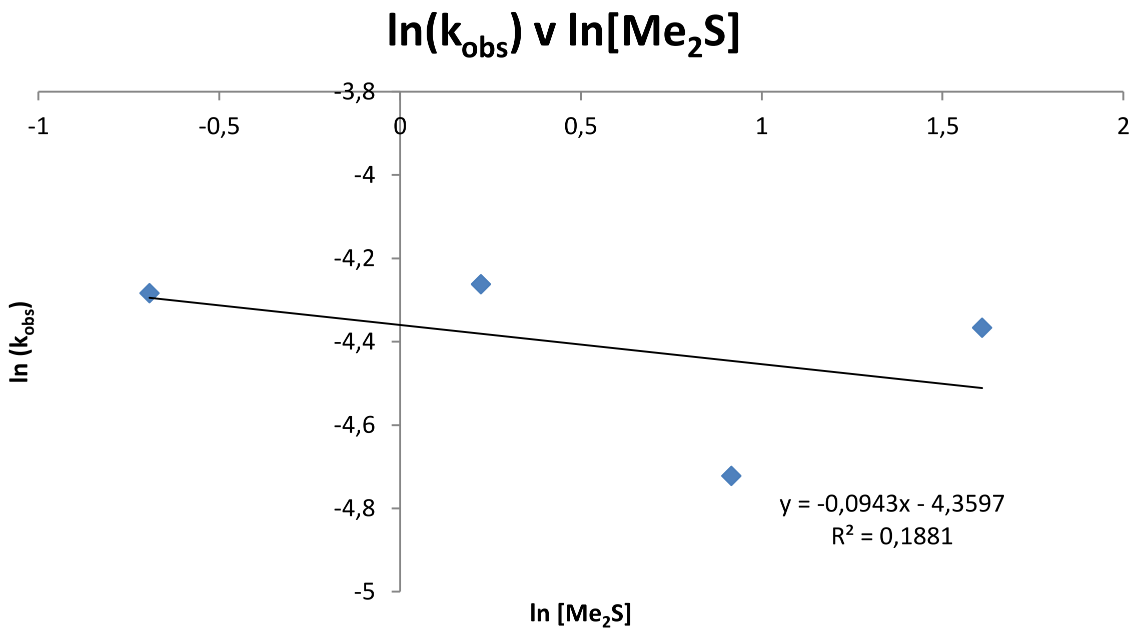

Figure S10. Plot of $\ln(k_{obs})$ against $\ln[Me_2S]$, showing essentially zero order dependence.

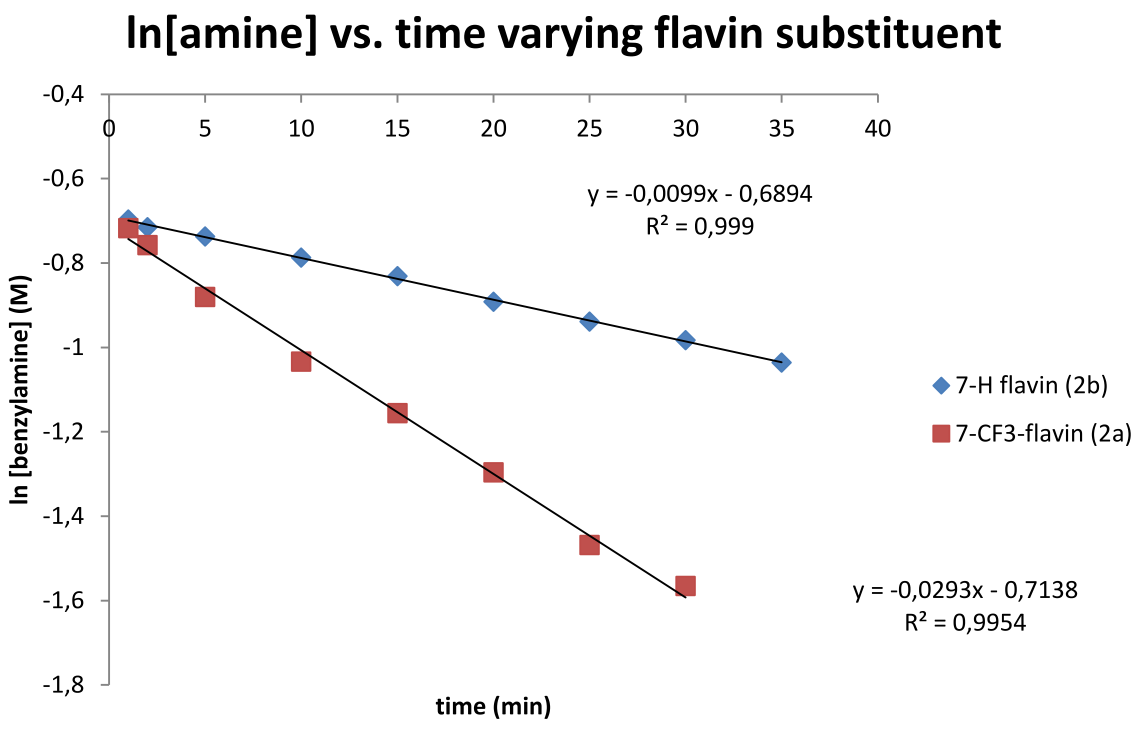

Figure S11. Plot of ln[**1a**], showing the effect of flavin electronic properties.

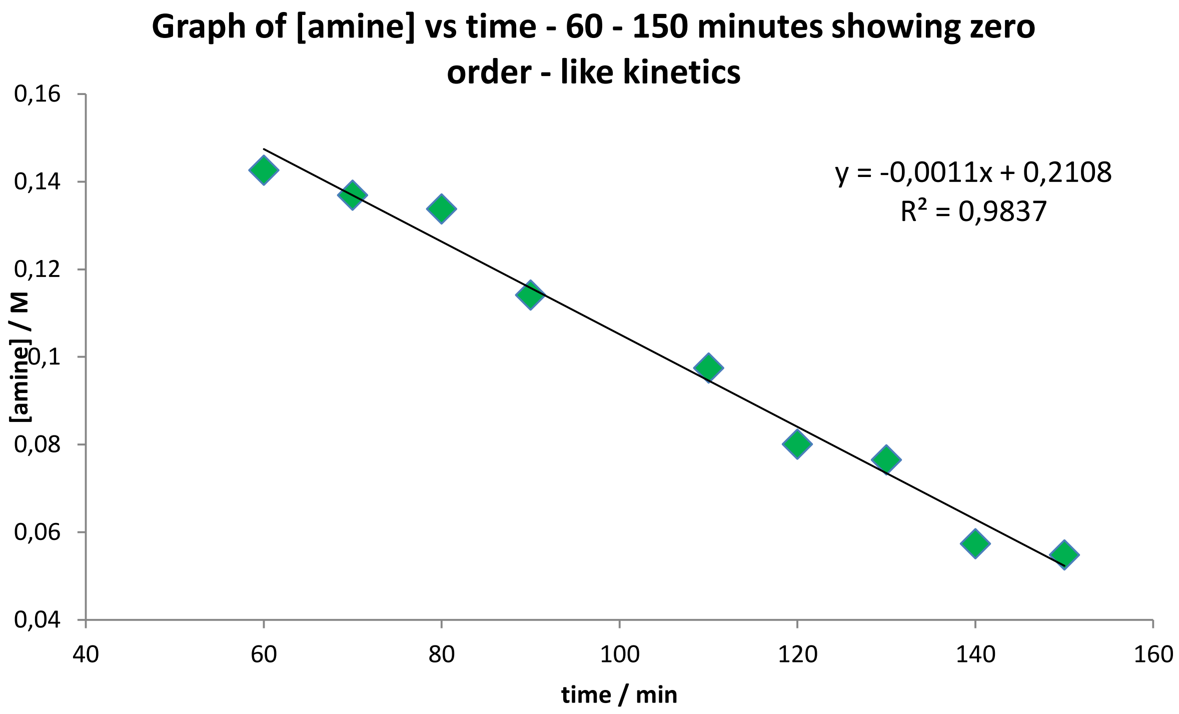

Figure S12. Demonstration of varying kinetic behaviour after 1 h of reaction – possible O_2 rate limitation?

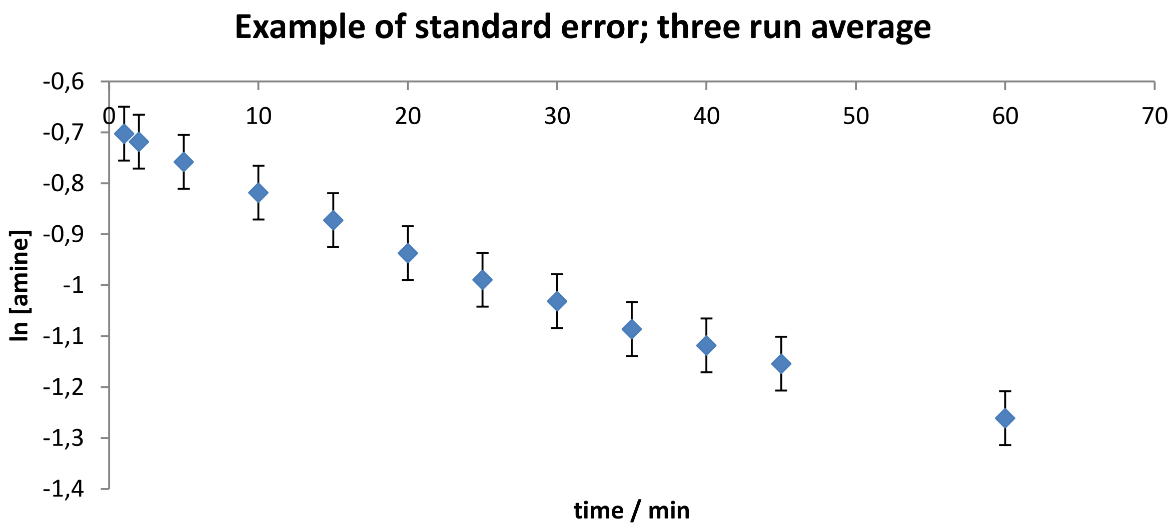

Figure S13. Example standard error over three runs using standard conditions

Preparation of aerobic flavin radical 2a’

To a solution of flavinium chloride **2a** (20 mg, 50 μmol) in 2,2,2-trifluoroethanol (1 mL) was added dimethyl sulfide (370 μL, 5 mmol), and the solution was swirled under air for 1 minute. The solution was then used for EPR analysis. Alternatively, for analysis in deuterated solvent, d_3-2,2,2-trifluoroethanol was instead used.

Preparation of anaerobic flavin radical 2a’

To a solution of flavinium chloride **2a** (20 mg, 50 μmol) in 2,2,2-trifluoroethanol (1 mL) was added dimethyl sulfide (370 μL, 5 mmol). The solution was purged with nitrogen for 10 minutes, then taken up into a 4 mm X-band EPR tube and opened inside a double-ended Schlenk tube. The solution was frozen and then subjected to four freeze-pump-thaw cycles, then sealed under vacuum and used for EPR analysis. Alternatively, for analysis in deuterated solvent, d_3-2,2,2-trifluoroethanol was instead used.

Preparation of aerobic aminyl radical 4a’

To a solution of flavinium chloride **2a** (20 mg, 50 μmol) and alloxan monohydrate (4 mg, 25 μmol) in 2,2,2-trifluoroethanol (1 mL) was added dimethyl sulfide (370 μL, 5 mmol), and the solution was swirled under air for 1 minute then left under air for a further 4 minutes. Benzylamine **1a** (55 μL, 0.5 mmol) was added, and the solution was then used for EPR analysis. Alternatively, for analysis in deuterated solvent, d_3-2,2,2-trifluoroethanol was instead used. For experiments with deuterated substrate, $PhCD_2NH_2$ **7** was used in place of benzylamine.

Preparation of anaerobic aminyl radical 4a'

To a solution of flavinium chloride **x** (20 mg, 50 µmol) and alloxan monohydrate (4 mg, 25 µmol) in 2,2,2-trifluoroethanol (1 mL) was added dimethyl sulfide (370 µL, 5 mmol), and the solution was purged with nitrogen for 10 minutes. Benzylamine (55 µL, 0.5 mmol) was added, and the solution was then taken up into a 4 mm X-band EPR tube and opened inside a double-ended Schlenk tube. The solution was frozen and then subjected to four freeze-pump-thaw cycles, then sealed under vacuum and used for EPR analysis. Alternatively, for analysis in deuterated solvent, d_3-2,2,2-trifluoroethanol was instead used. For experiments with deuterated substrate, $PhCD_2NH_2$ **7** was used in place of benzylamine.

Further EPR discussion

At X-band (~ 9.7 GHz), the continuous wave EPR experiments allow us to characterize the parameters defining the strongest interaction terms of the time-independent spin-Hamiltonian.[9] The fact that the *g* factor reflects the global electronic structure, while the hyperfine structure is determined by the local spin density distribution in the vicinity of the nuclei such as EPR-active ^{14}N, ^{1}H, ^{19}F and EPR-silent ^{12}C, ^{16}O of the flavin heterocyclic ring. The flavin semiquinone **2a'** sample is a liquid solution at 298 K, and the radicals have virtually a non-restricted molecular degree of freedom, e.g. free tumbling or rapid reorientation, and any anisotropy in the *g* factor and in the hyperfine splittings is completely averaged out. Analysis of the liquid EPR spectrum does not generally identify the interacting nuclei. To circumvent this limitation and to assign nuclear hyperfine splittings, $^{2}H/^{1}H$ substitution of solvent (CF_3CD_2OD/CF_3CH_2OH) was used and the splitting patterns of the hyperfine multiplet subsequently were not affected, except for a slight narrowing of the linewidth of the absorption first-derivative lines. As the radicals are tumbling or reorienting, the isotropic coupling to the three nearly equivalent ^{14}N nuclei gives rise to the well-resolved hyperfine structure. The isotropic hyperfine coupling accounts for ~ 22 MHz (0.8 mT) and consequently the unpaired electron has spin density distribution of ~ 20% in the 2*p* orbital of a ^{14}N atom. The isotropic hyperfine coupling is resolved when the unpaired electron resides in a higher *p* orbital, and spin density in the *s* orbital is induced by configuration interactions or usually through a spin polarization mechanism.[10] The protons lie in the nodal plane of the $\boldsymbol{\pi}$ orbital occupied by the unpaired electron of the semiquinone flavin ring. Even the largest proton coupling of ~ 21 MHz (0.75 mT) only amounts to a fractional density distribution of ~ 1.5% in the *s* orbital of a hydrogen atom where $|\boldsymbol{\Psi}_0(0)|^2 = 1$ and 1423 MHz (508 mT).[9] The hyperfine splitting from ^{19}F is usually indistinguishable from ring proton hyperfine splittings and are too small to be observed in the hyperfine structure, only having a negligible broadening effect on the linewidth across the hyperfine splitting set. It is obvious that spin density is mostly distributed on the *p* orbitals of the semiquinone flavin radical atoms, so the hyperfine structure of the first-derivative spectrum arises from the interaction of the unpaired electron spin with the nitrogen nuclei. In order to justify the experimental parameters defining the electron Zeeman and hyperfine interaction terms in the spin-Hamiltonian, the numerical simulations and fittings have been performed and consequently the agreements between the experimental and calculated spectra are quite satisfactory.

Other evidence was further required to demonstrate the interacting ^{14}N nuclei. For this purpose, the pulsed EPR experiment, namely hyperfine sublevel correlation spectroscopy (HYSCORE),[11] is used to obtain information about the surrounding magnetic nuclei around the radical unpaired electron spin. This 2D ESEEM method has the ability to resolve the weak hyperfine interactions (<5 MHz) of the remote nuclei with the unpaired electron spin, which are masked or suppressed by EPR broadening in the hyperfine structure. ESEEM appears only if the allowed and forbidden EPR transitions are simultaneously induced by the high power microwave pulses.[12] In the case of the ^{14}N nucleus with the spin I = 1, the time-independent spin-Hamiltonian includes an additional nuclear quadrupole interaction term.[12] The ESEEM spectrum expected from ^{14}N with a predominantly isotropic hyperfine coupling is governed by the ratio between the effective nuclear frequency in each electron spin m_S = ±1/2 manifold, $\nu_{ef\pm} = |^{14}\nu_N \pm {}^{14}A_N/2|$, and quadrupole coupling constant $K = e^2Qq/4h$.

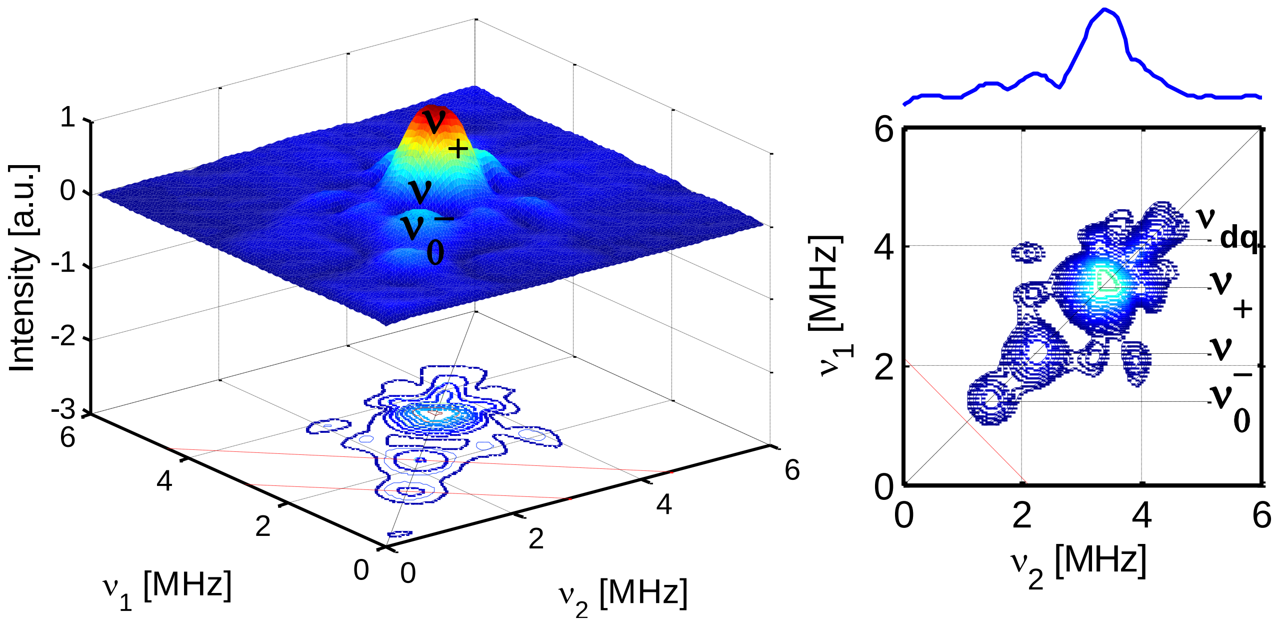

Figure S14. Left: contour presentation of the X-band natural abundance ^{14}N HYSCORE spectrum (**bottom**) of flavin semiquinone **2a'** recorded at 345 mT and 9.685 GHz. Time between the first and second pulses was τ = 200 ns. Relative intensities of the diagonal contour-peaks, marked for nuclear quadrupole resonance frequencies ν_0, ν_-, and ν_+, are shown as an absolute spectrum (**top**) in a horizontal projection. The dashed red line represents the nitrogen nuclear Zeeman frequency, $^{14}\nu_N$ ~ 1.06 MHz.

Right: a contour plot (**bottom**) and full 3D view (**top**) of the ^{14}N HYSCORE spectrum clearly shows differences in relative intensities, especially $\nu_+ \approx$ 3.4 MHz component dominating $\nu_0 \approx$ 1.4 MHz and $\nu_- \approx$ 2.2 MHz components marked by the two red dashed lines in the contour plot, and these zero-field quadrupole resonance frequencies correlate with the double-quantum frequency $\nu_{dq} \approx$ 4.2 MHz (left contour plot) from the opposite electron spin manifold. The normalized intensity is in arbitrary unit in 3D plot.

Accordingly, the X-band ^{14}N HYSCORE spectrum resolves the three contour-peaks from the three nuclear frequencies, nearly satisfying the "cancellation condition" $|^{14}\nu_N - {}^{14}A_N/2| \approx 0$ in one of the electron spin manifolds (**Figure S12**). These frequencies, with the property $\nu_+ = \nu_0 + \nu_-$, are slightly broadened here as $\nu_{ef\pm}/K$ deviates from 0. However, these frequencies can appear in the spectrum up to a ratio $\nu_{ef\pm}/K$ ~ 0.75-1.[13] In orientation disordered samples,

such as frozen solutions used in this work, not all transitions contribute equally to the spectrum due to different orientation.[14] In our case, the component ν_+ dominates the other two ν_0 and ν_- components. The intensity of contour-peaks is significantly influenced by the selection of the time τ between the first and second pulses. Because of this property, several measurements at different, fixed τ times were performed to detect all contour-peaks contributing to the HYSCORE spectrum. The three nuclear frequencies, assigned to the zero-field nuclear quadrupole resonance frequencies ν_0, ν_-, and ν_+, correlate with the double-quantum transition ν_{dq} frequency from the opposite spin manifold. The nuclear quadrupole and hyperfine couplings are of the order $K \approx 0.96$ MHz and $a \approx 1.46$ MHz, within experimental error, assigned to the $^{14}N(3)$ or $^{14}N(1)$. It became possible to determine the reduced magnitude of relatively larger hyperfine couplings from other nitrogen atoms following the $^2H/^1H$ substitution of solvent around the flavin ring. In this case, spin density distribution among the flavin ring atoms is slightly perturbed.

Upon addition of alloxan and amine, a completely new EPR spectrum was observed which appears to correspond to the dibenzylaminyl radical, consistent with an H-abstraction mechanism. This radical demonstrated long-term stability and therefore is consistent with an oxygen-starved resting state. These EPR experiments strongly suggested a reaction mechanism with inherent radical character. For this radical, several proton nuclei (I = 1/2) are nearly equally coupled, i.e. possessing almost the same isotropic couplings. The radical contains protons on its two rings that are grouped into the two nearly equivalent sets but with slightly different isotropic couplings of ~ 11 MHz and ~ 9 MHz, respectively. Again, the spin polarization mechanism is responsible for the resolved hyperfine splittings from these ring protons. The central nitrogen atom carries a significant spin density distribution to contribute to a slight asymmetry of the absorption derivative; as a result its neighbouring two protons also possess the same order of the isotropic coupling ~ 13 MHz.

COMPUTATIONAL AND THEORETICAL DETAILS

All quantum-chemical calculations were performed using the AIMS package.[15] Within this all-electron approach the electronic wavefunctions are constructed using numeric atom-centred basis functions. A converged `tight' basis set was employed, which includes *d*, *f* and *g* functions on the N atoms, and scalar relativistic effects were included.

Local structure optimisations were performed using the forces from density functional theory (DFT) using the PBE exchange-correlation potential.[16] Additional tests were performed using the hybrid PBE0 and B3LYP exchange-correlation functionals,[17] which were found not to change the localisation of the radical spin.

The inclusion of a polarisable continuum would not change the qualitative trends reported here. Indeed the current model has been shown to reproduce other similar experiments to within 3.5% of the measured IP.[18] Whilst solvation of radicals in aqueous media has been observed,[19] our model can be considered representative of certain complex biological systems. The internal chemistry of a protein, the site of electron hoping, is defined by motifs similar to what is studied here: organic molecules with ubiquitous heteroatoms, sparsely hydrated.

AM552

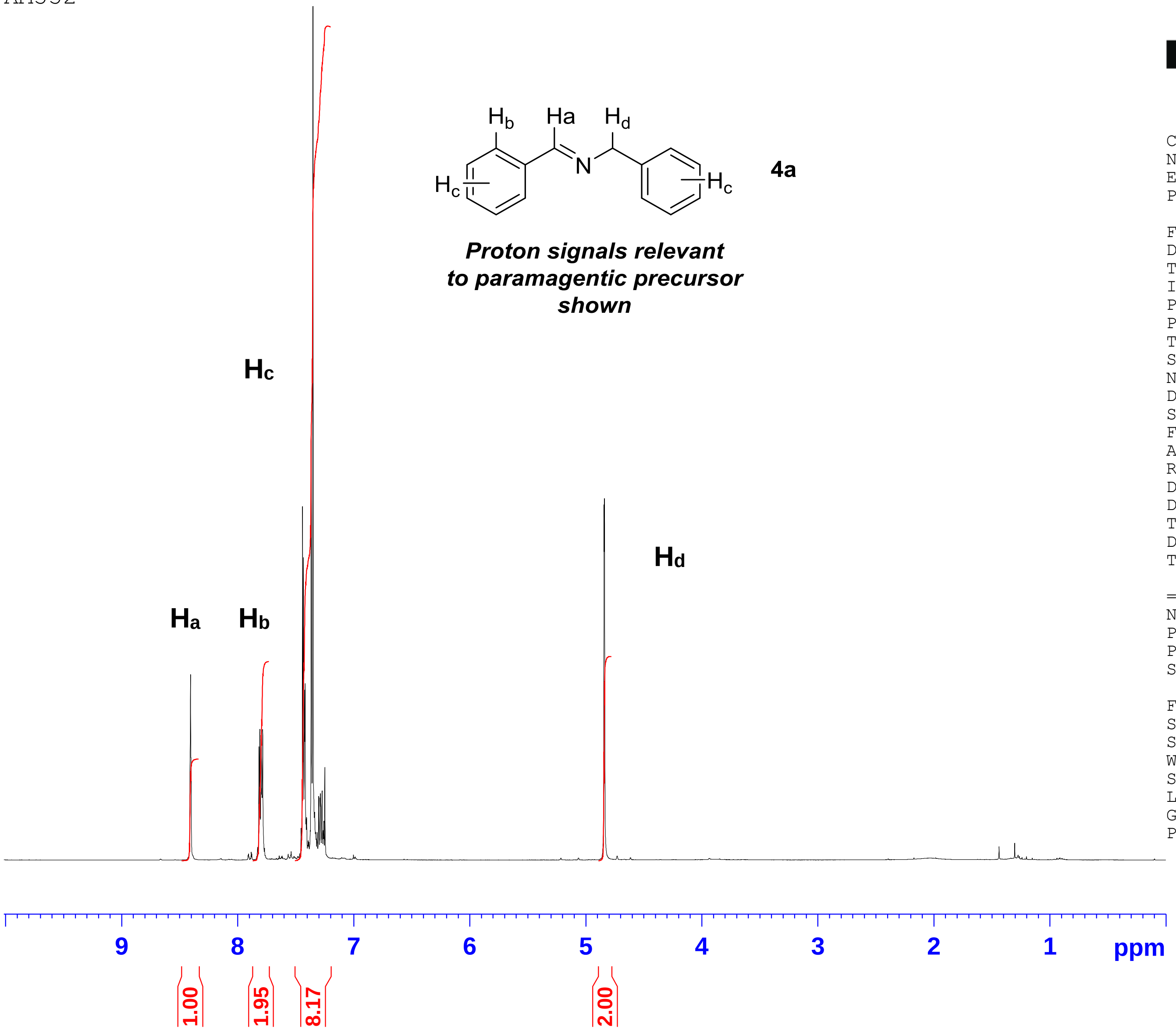


```
Current Data Parameters
NAME              Nov20-2012
EXPNO                     20
PROCNO                     1

F2 - Acquisition Parameters
Date_               20121121
Time                    5.32
INSTRUM                av300
PROBHD     5 mm BBO BB-1H
PULPROG                 zg30
TD                     32768
SOLVENT                CDCl3
NS                        32
DS                         0
SWH             6172.839 Hz
FIDRES          0.188380 Hz
AQ             2.6542079 sec
RG                       181
DW               81.000 usec
DE                 6.00 usec
TE                 673.2 K
D1            1.00000000 sec
TD0                        1

======== CHANNEL f1 ========
NUC1                      1H
P1                 7.10 usec
PL1                -1.50 dB
SFO1         300.2218540 MHz

F2 - Processing parameters
SI                     32768
SF           300.2200060 MHz
WDW                       EM
SSB        0
LB                  0.30 Hz
GB         0
PC                      1.00
```

AM552

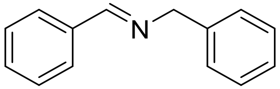

4a

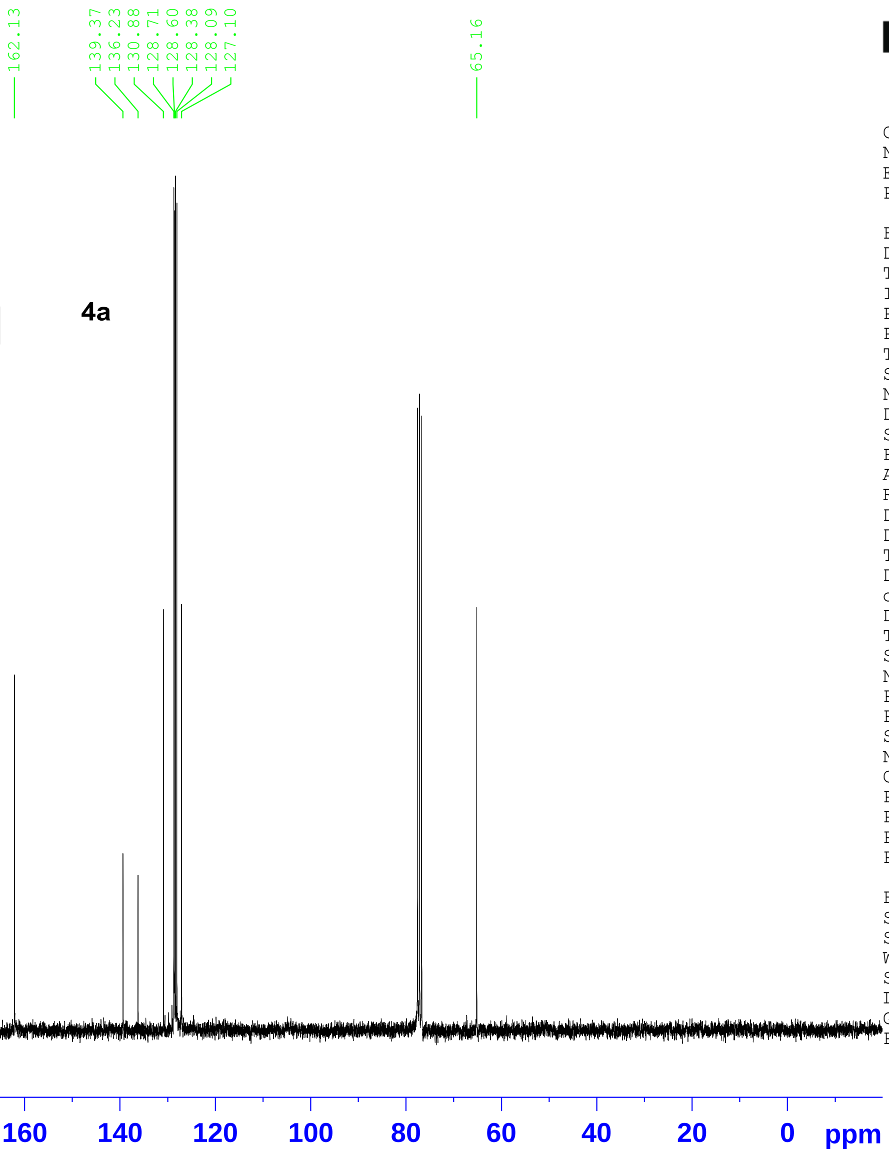

BRUKER

```
Current Data Parameters
NAME             Nov20-2012
EXPNO                    21
PROCNO                    1

F2 - Acquisition Parameters
Date_              20121121
Time                   5.49
INSTRUM               av300
PROBHD   5 mm BBO BB-1H
PULPROG             zgpg30
TD                    65536
SOLVENT               CDCl3
NS                      256
DS                        2
SWH              20325.203 Hz
FIDRES            0.310138 Hz
AQ               1.6121856 sec
RG                 18390.4
DW                  24.600 usec
DE                    6.00 usec
TE                   673.2 K
D1              2.00000000 sec
d11             0.03000000 sec
DELTA           1.89999998 sec
TD0                       1
SFO1            75.4990304 MHz
NUC1                    13C
P1                    7.50 usec
PLW1           -1.00000000 W
SFO2           300.2212009 MHz
NUC2                     1H
CPDPRG[2            waltz16
PCPD2                80.00 usec
PLW2           -1.00000000 W
PLW12          -1.00000000 W
PLW13          -1.00000000 W

F2 - Processing parameters
SI                    65536
SF              75.4903736 MHz
WDW                      EM
SSB       0
LB                     1.00 Hz
GB        0
PC                     1.40
```

MD041

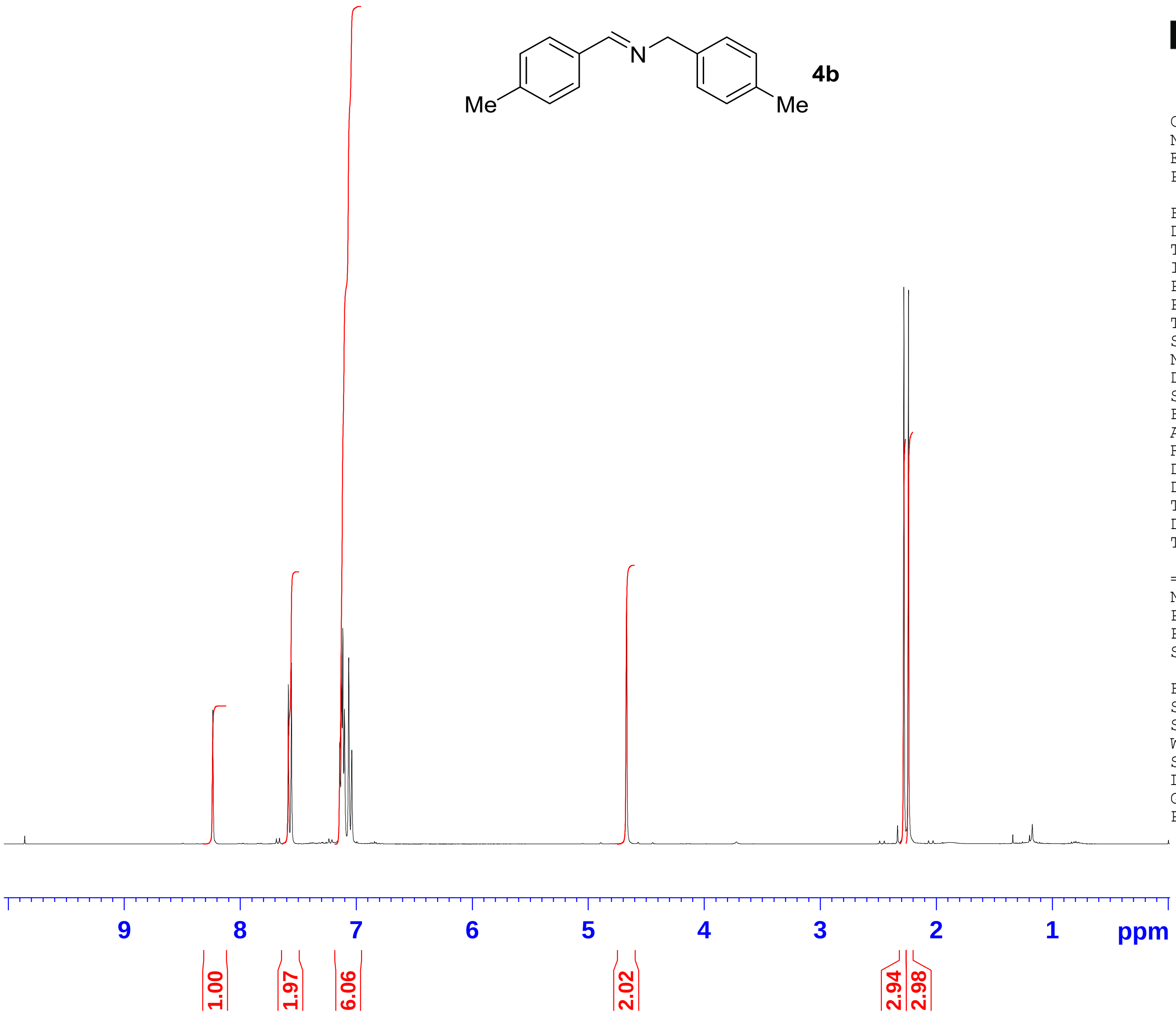


```
Current Data Parameters
NAME            Nov16-2012
EXPNO                   10
PROCNO                   1

F2 - Acquisition Parameters
Date_             20121116
Time                 22.37
INSTRUM              av300
PROBHD   5 mm BBO BB-1H
PULPROG               zg30
TD                   32768
SOLVENT              CDCl3
NS                      32
DS                       0
SWH               6172.839 Hz
FIDRES            0.188380 Hz
AQ               2.6542079 sec
RG                     114
DW                  81.000 usec
DE                    6.00 usec
TE                   673.2 K
D1              1.00000000 sec
TD0                      1

======== CHANNEL f1 ========
NUC1                    1H
P1                    7.10 usec
PL1                  -1.50 dB
SFO1           300.2218540 MHz

F2 - Processing parameters
SI                   32768
SF             300.2200420 MHz
WDW                     EM
SSB      0
LB                    0.30 Hz
GB       0
PC                    1.00
```

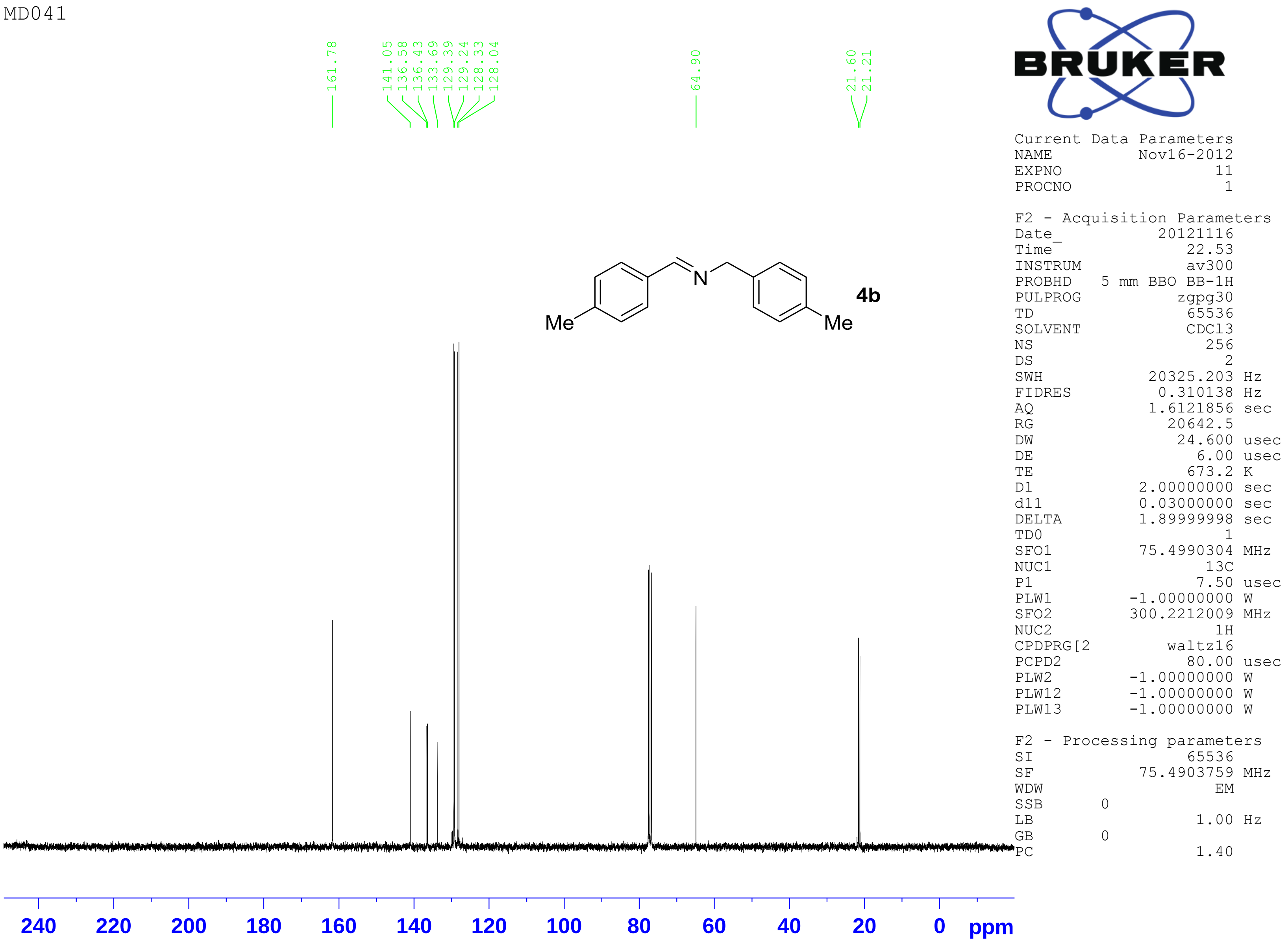
MD041
161.78
141.05
136.58
136.43
133.69
129.39
129.24
128.33
128.04
64.90
21.60
21.21
N
Me
Me
4b
240
220
200
180
160
140
120
100
80
60
40
20
0
ppm
BRUKER
Current Data Parameters
NAME Nov16-2012
EXPNO 11
PROCNO 1
F2 - Acquisition Parameters
Date_ 20121116
Time 22.53
INSTRUM av300
PROBHD 5 mm BBO BB-1H
PULPROG zgpg30
TD 65536
SOLVENT CDCl3
NS 256
DS 2
SWH 20325.203 Hz
FIDRES 0.310138 Hz
AQ 1.6121856 sec
RG 20642.5
DW 24.600 usec
DE 6.00 usec
TE 673.2 K
D1 2.00000000 sec
d11 0.03000000 sec
DELTA 1.89999998 sec
TD0 1
SFO1 75.4990304 MHz
NUC1 13C
P1 7.50 usec
PLW1 -1.00000000 W
SFO2 300.2212009 MHz
NUC2 1H
CPDPRG[2 waltz16
PCPD2 80.00 usec
PLW2 -1.00000000 W
PLW12 -1.00000000 W
PLW13 -1.00000000 W
F2 - Processing parameters
SI 65536
SF 75.4903759 MHz
WDW EM
SSB 0
LB 1.00 Hz
GB 0
PC 1.40

AM510

4c

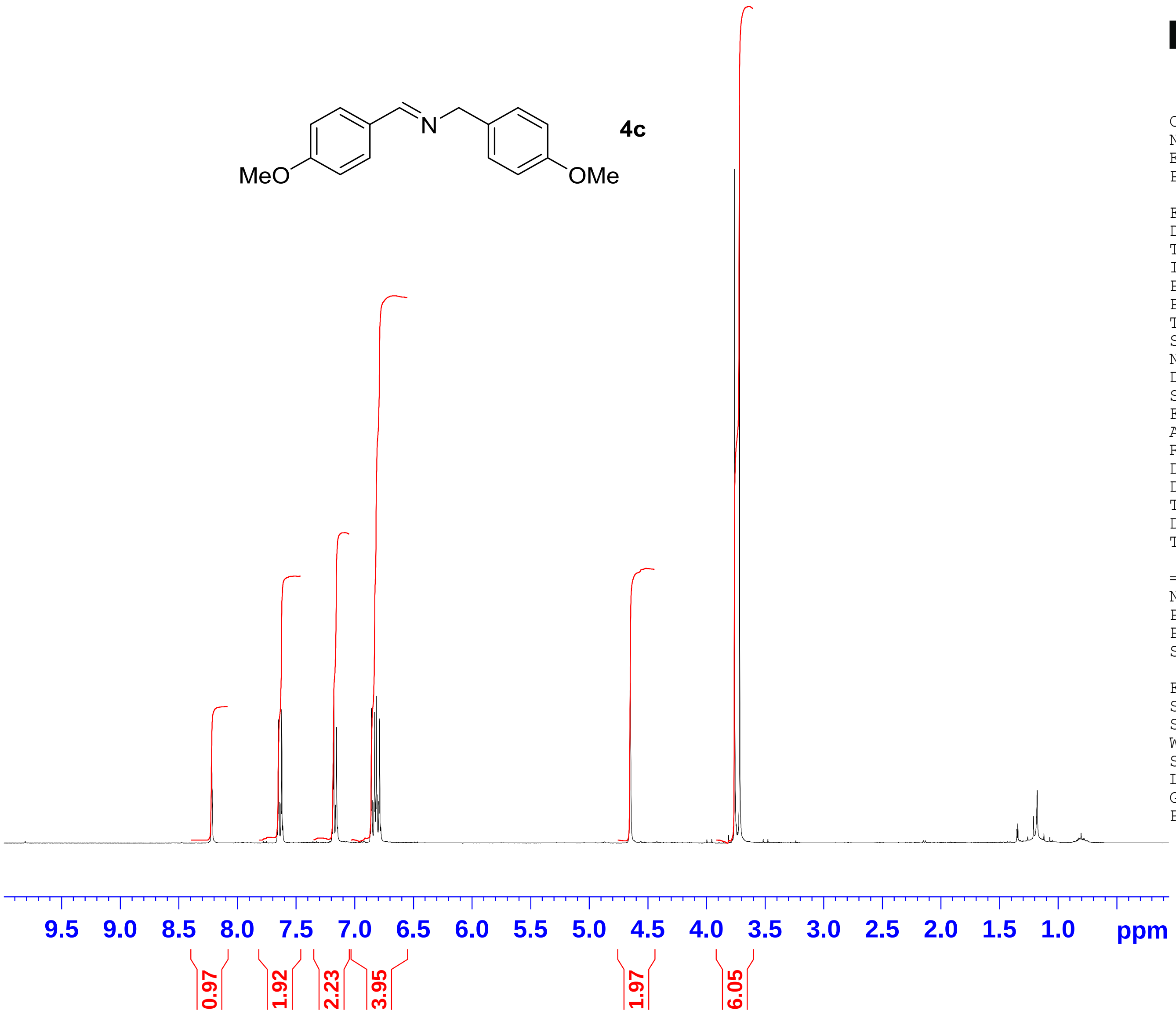

Current Data Parameters		
NAME	26102012-atm	
EXPNO	10	
PROCNO	1	
F2 - Acquisition Parameters		
Date_	20121026	
Time	13.09	
INSTRUM	av300	
PROBHD	5 mm BBO BB-1H	
PULPROG	zg30	
TD	32768	
SOLVENT	CDCl3	
NS	32	
DS	0	
SWH	6172.839	Hz
FIDRES	0.188380	Hz
AQ	2.6542079	sec
RG	181	
DW	81.000	usec
DE	6.00	usec
TE	292.4	K
D1	1.00000000	sec
TD0	1	
======== CHANNEL f1 ========		
NUC1	1H	
P1	7.10	usec
PL1	-1.50	dB
SFO1	300.2218540	MHz
F2 - Processing parameters		
SI	32768	
SF	300.2200272	MHz
WDW	EM	
SSB	0	
LB	0.30	Hz
GB	0	
PC	1.00	

AM510

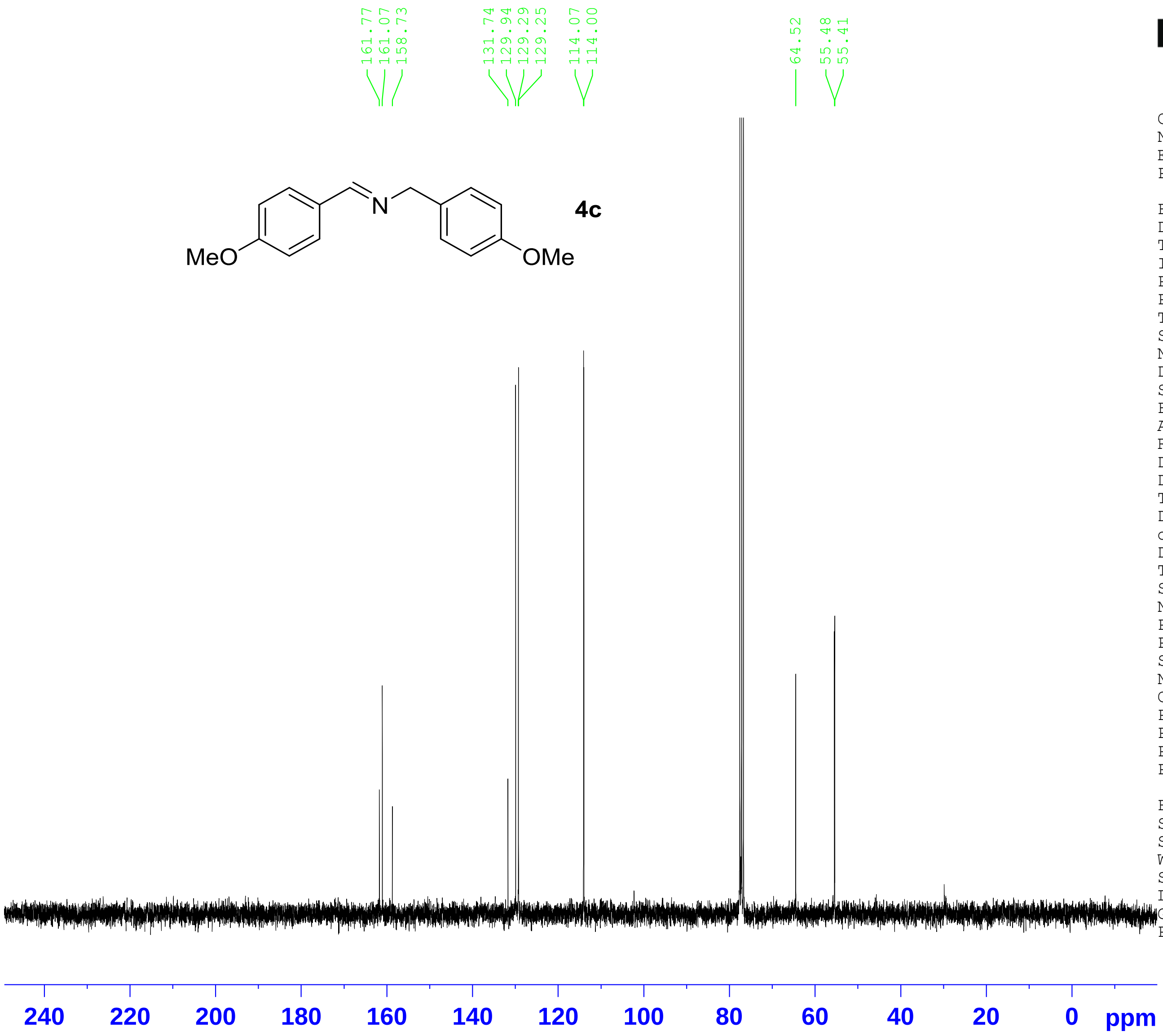

BRUKER

```
Current Data Parameters
NAME          26102012-atm
EXPNO                   11
PROCNO                   1

F2 - Acquisition Parameters
Date_             20121026
Time                 13.27
INSTRUM              av300
PROBHD    5 mm BBO BB-1H
PULPROG            zgpg30
TD                   65536
SOLVENT              CDCl3
NS                     256
DS                       2
SWH              20325.203 Hz
FIDRES            0.310138 Hz
AQ               1.6121856 sec
RG                 11585.2
DW                  24.600 usec
DE                    6.00 usec
TE                   293.0 K
D1              2.00000000 sec
d11             0.03000000 sec
DELTA           1.89999998 sec
TD0                      1
SFO1            75.4990304 MHz
NUC1                   13C
P1                    7.50 usec
PLW1           -1.00000000 W
SFO2           300.2212009 MHz
NUC2                    1H
CPDPRG[2           waltz16
PCPD2                80.00 usec
PLW2           -1.00000000 W
PLW12          -1.00000000 W
PLW13          -1.00000000 W

F2 - Processing parameters
SI                   65536
SF              75.4903707 MHz
WDW                     EM
SSB       0
LB                    1.00 Hz
GB        0
PC                    1.40
```

AM549

4d

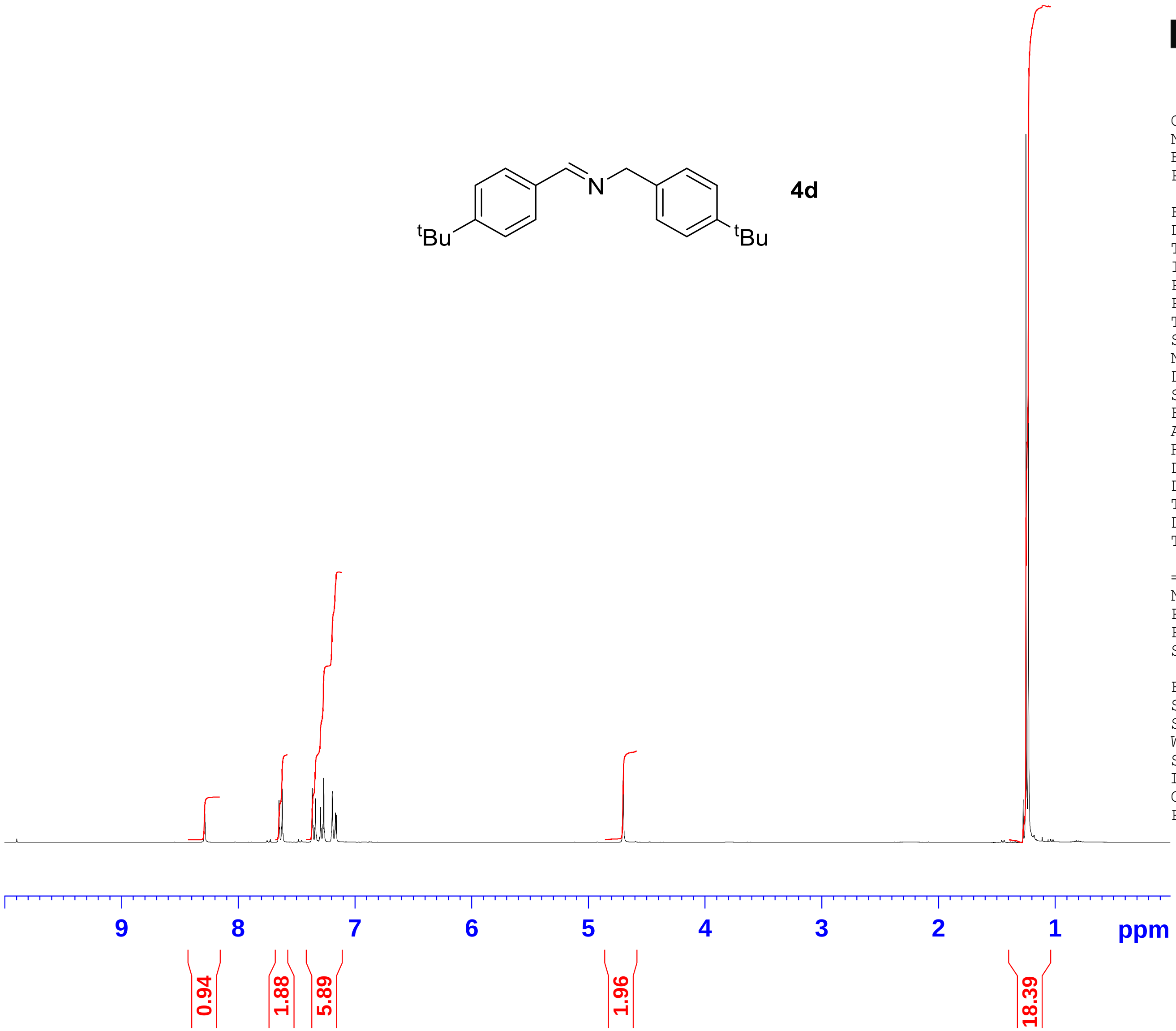


```
Current Data Parameters
NAME            Nov16-2012
EXPNO                   30
PROCNO                   1

F2 - Acquisition Parameters
Date_             20121117
Time                  2.34
INSTRUM              av300
PROBHD     5 mm BBO BB-1H
PULPROG               zg30
TD                   32768
SOLVENT              CDCl3
NS                      32
DS                       0
SWH               6172.839 Hz
FIDRES            0.188380 Hz
AQ               2.6542079 sec
RG                     181
DW                  81.000 usec
DE                    6.00 usec
TE                   673.2 K
D1              1.00000000 sec
TD0                      1

======== CHANNEL f1 ========
NUC1                    1H
P1                    7.10 usec
PL1                  -1.50 dB
SFO1           300.2218540 MHz

F2 - Processing parameters
SI                   32768
SF             300.2200333 MHz
WDW                     EM
SSB      0
LB                    0.30 Hz
GB       0
PC                    1.00
```

AM549

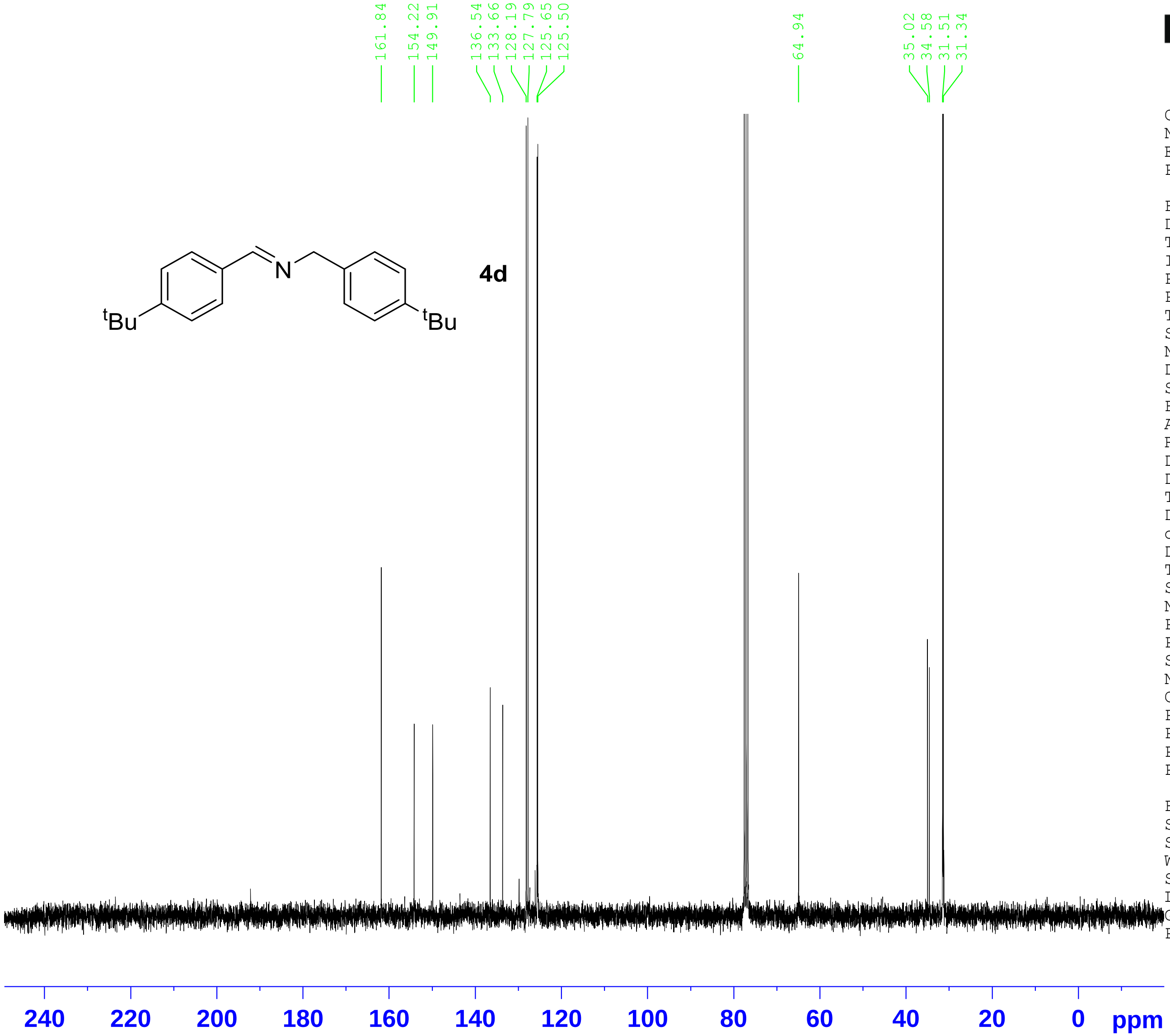

4d

BRUKER

Current Data Parameters
NAME Nov16-2012
EXPNO 31
PROCNO 1

F2 - Acquisition Parameters

Date_	20121117	
Time	2.51	
INSTRUM	av300	
PROBHD	5 mm BBO BB-1H	
PULPROG	zgpg30	
TD	65536	
SOLVENT	CDCl3	
NS	256	
DS	2	
SWH	20325.203	Hz
FIDRES	0.310138	Hz
AQ	1.6121856	sec
RG	9195.2	
DW	24.600	usec
DE	6.00	usec
TE	673.2	K
D1	2.00000000	sec
d11	0.03000000	sec
DELTA	1.89999998	sec
TD0	1	
SFO1	75.4990304	MHz
NUC1	13C	
P1	7.50	usec
PLW1	-1.00000000	W
SFO2	300.2212009	MHz
NUC2	1H	
CPDPRG[2	waltz16	
PCPD2	80.00	usec
PLW2	-1.00000000	W
PLW12	-1.00000000	W
PLW13	-1.00000000	W

F2 - Processing parameters

SI	65536	
SF	75.4903720	MHz
WDW	EM	
SSB	0	
LB	1.00	Hz
GB	0	
PC	1.40	

MD056

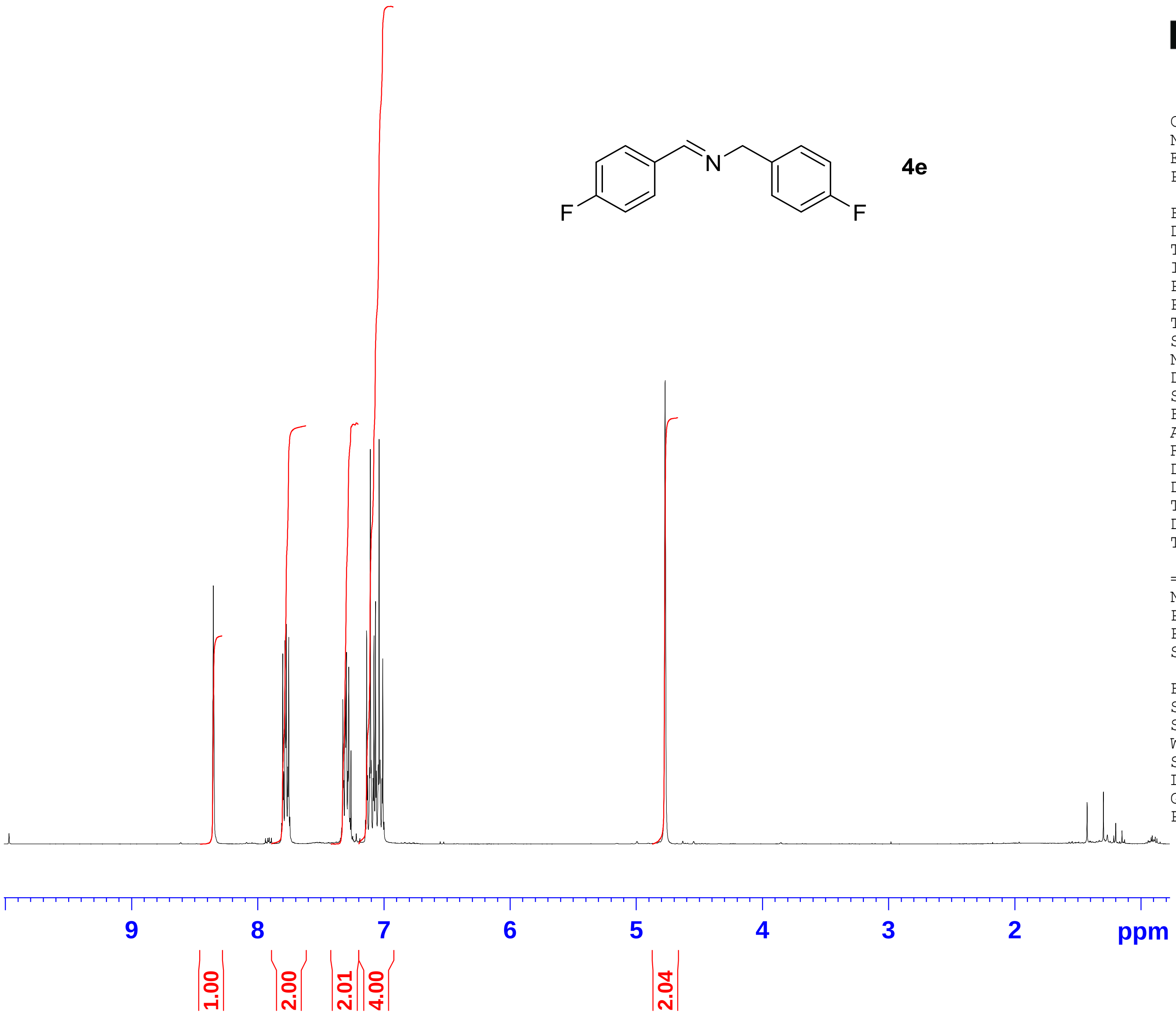


```
Current Data Parameters
NAME       Feb11-2013-md1
EXPNO                  10
PROCNO                  1

F2 - Acquisition Parameters
Date_            20130211
Time                11.08
INSTRUM             av300
PROBHD     5 mm BBO BB-1H
PULPROG              zg30
TD                  32768
SOLVENT             CDCl3
NS                     32
DS                      0
SWH              6172.839 Hz
FIDRES           0.188380 Hz
AQ              2.6542079 sec
RG                    181
DW                 81.000 usec
DE                   6.00 usec
TE                  673.2 K
D1             1.00000000 sec
TD0                     1

======== CHANNEL f1 ========
NUC1                   1H
P1                   7.10 usec
PL1                 -1.50 dB
SFO1          300.2218540 MHz

F2 - Processing parameters
SI                  32768
SF            300.2200023 MHz
WDW                    EM
SSB       0
LB                   0.30 Hz
GB        0
PC                   1.00
```

MD056

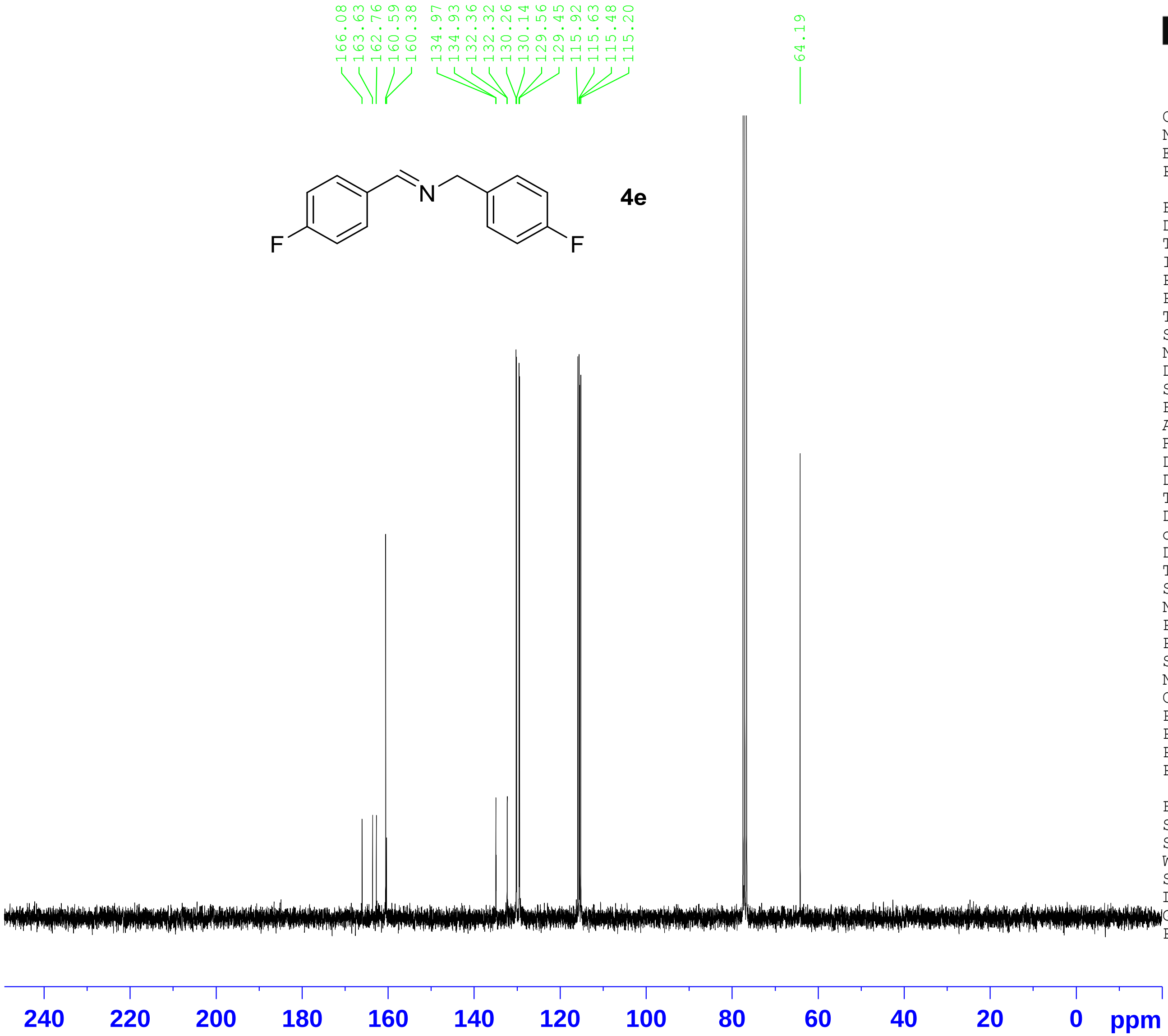

4e

Current Data Parameters
NAME Feb11-2013-md1
EXPNO 11
PROCNO 1

F2 - Acquisition Parameters

Date_	20130211	
Time	11.25	
INSTRUM	av300	
PROBHD	5 mm BBO BB-1H	
PULPROG	zgpg30	
TD	65536	
SOLVENT	CDCl3	
NS	256	
DS	2	
SWH	20325.203	Hz
FIDRES	0.310138	Hz
AQ	1.6121856	sec
RG	16384	
DW	24.600	usec
DE	6.00	usec
TE	673.2	K
D1	2.00000000	sec
d11	0.03000000	sec
DELTA	1.89999998	sec
TD0	1	
SFO1	75.4990304	MHz
NUC1	13C	
P1	7.50	usec
PLW1	-1.00000000	W
SFO2	300.2212009	MHz
NUC2	1H	
CPDPRG[2	waltz16	
PCPD2	80.00	usec
PLW2	-1.00000000	W
PLW12	-1.00000000	W
PLW13	-1.00000000	W

F2 - Processing parameters

SI	65536	
SF	75.4903777	MHz
WDW	EM	
SSB	0	
LB	1.00	Hz
GB	0	
PC	1.40	

MD045B

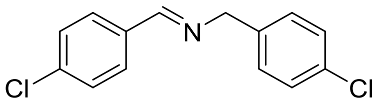

4f

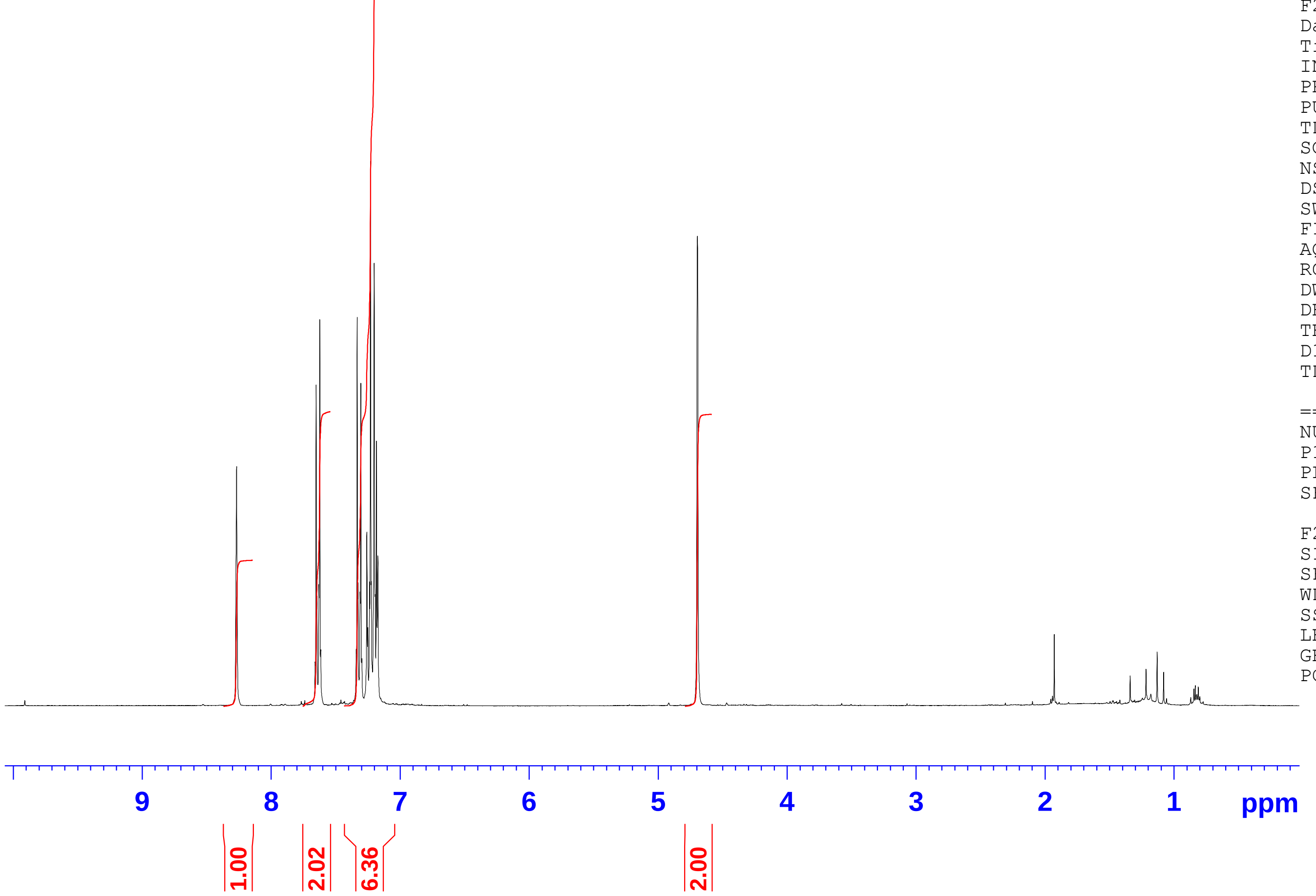


```
Current Data Parameters
NAME       Nov21-2012-md1
EXPNO                  10
PROCNO                  1

F2 - Acquisition Parameters
Date_            20121121
Time                14.12
INSTRUM             av300
PROBHD     5 mm BBO BB-1H
PULPROG              zg30
TD                  32768
SOLVENT             CDCl3
NS                     32
DS                      0
SWH              6172.839 Hz
FIDRES           0.188380 Hz
AQ              2.6542079 sec
RG                  322.5
DW                 81.000 usec
DE                   6.00 usec
TE                  673.2 K
D1             1.00000000 sec
TD0                     1

======== CHANNEL f1 ========
NUC1                   1H
P1                   7.10 usec
PL1                 -1.50 dB
SFO1          300.2218540 MHz

F2 - Processing parameters
SI                  32768
SF            300.2200259 MHz
WDW                    EM
SSB        0
LB                   0.30 Hz
GB         0
PC                   1.00
```

MD045B

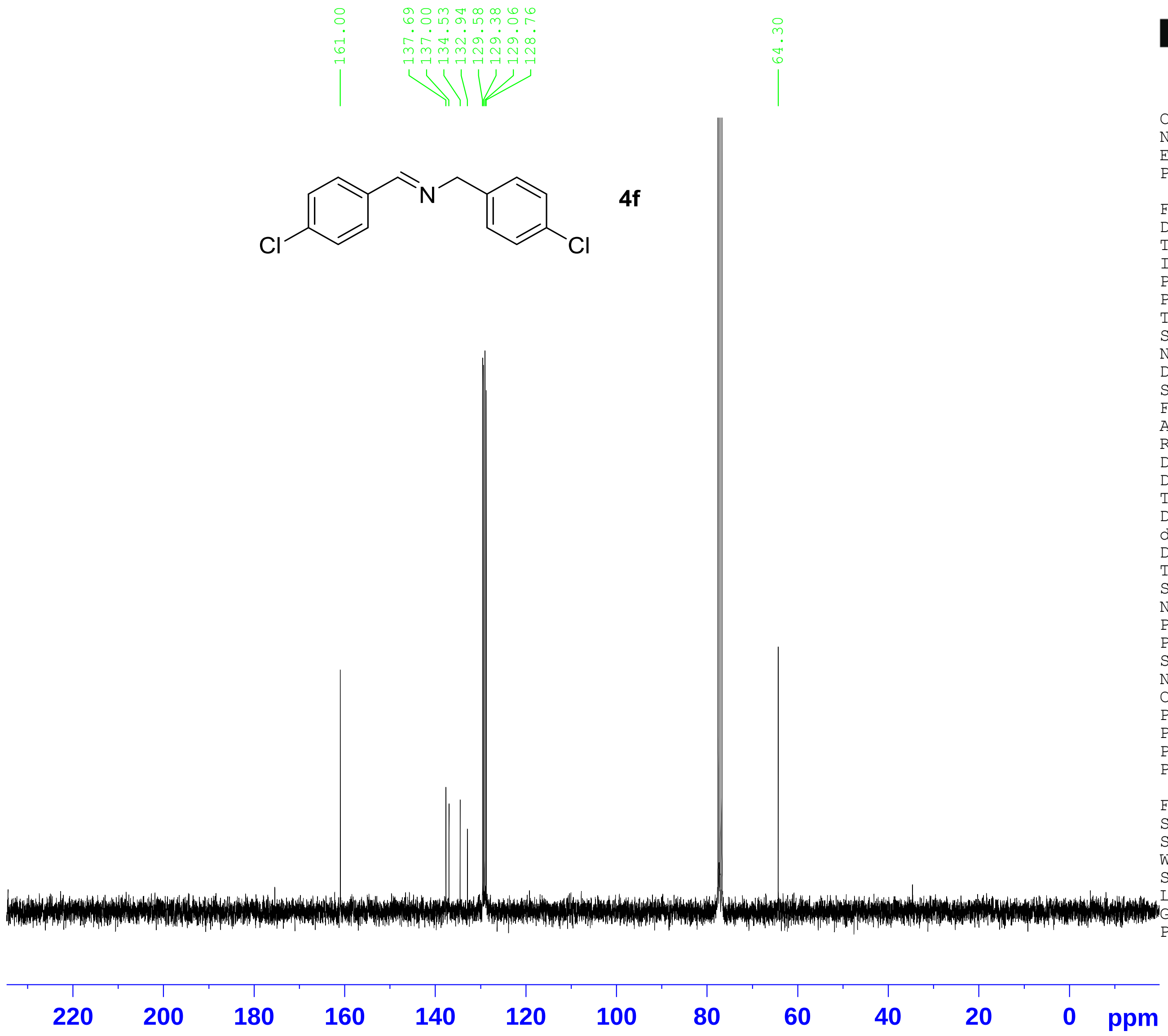

Current Data Parameters
NAME Nov21-2012-md1
EXPNO 11
PROCNO 1

F2 - Acquisition Parameters
Date_ 20121121
Time 14.29
INSTRUM av300
PROBHD 5 mm BBO BB-1H
PULPROG zgpg30
TD 65536
SOLVENT CDCl3
NS 256
DS 2
SWH 20325.203 Hz
FIDRES 0.310138 Hz
AQ 1.6121856 sec
RG 18390.4
DW 24.600 usec
DE 6.00 usec
TE 673.2 K
D1 2.00000000 sec
d11 0.03000000 sec
DELTA 1.89999998 sec
TD0 1
SFO1 75.4990304 MHz
NUC1 13C
P1 7.50 usec
PLW1 -1.00000000 W
SFO2 300.2212009 MHz
NUC2 1H
CPDPRG[2 waltz16
PCPD2 80.00 usec
PLW2 -1.00000000 W
PLW12 -1.00000000 W
PLW13 -1.00000000 W

F2 - Processing parameters
SI 65536
SF 75.4903709 MHz
WDW EM
SSB 0
LB 1.00 Hz
GB 0
PC 1.40

MD064

4g

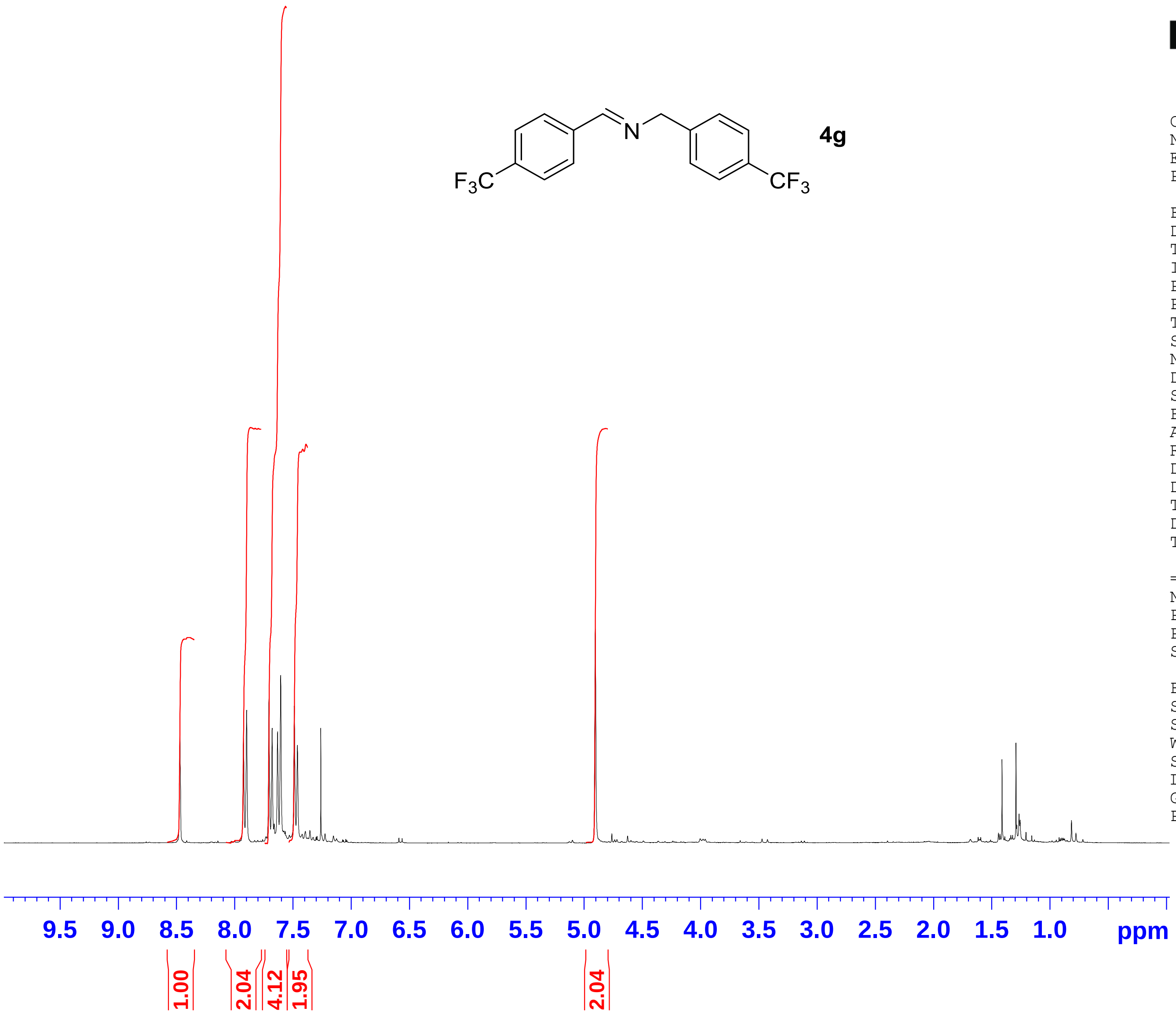


```
Current Data Parameters
NAME      Feb27-2013-md1
EXPNO                 10
PROCNO                 1

F2 - Acquisition Parameters
Date_           20130227
Time               15.16
INSTRUM            av300
PROBHD    5 mm BBO BB-1H
PULPROG             zg30
TD                 32768
SOLVENT            CDCl3
NS                    32
DS                     0
SWH             6172.839 Hz
FIDRES          0.188380 Hz
AQ             2.6542079 sec
RG                 287.4
DW                81.000 usec
DE                  6.00 usec
TE                 673.2 K
D1            1.00000000 sec
TD0                    1

======== CHANNEL f1 ========
NUC1                  1H
P1                  7.10 usec
PL1                -1.50 dB
SFO1         300.2218540 MHz

F2 - Processing parameters
SI                 32768
SF           300.2200029 MHz
WDW                   EM
SSB       0
LB                  0.30 Hz
GB        0
PC                  1.00
```

MD064

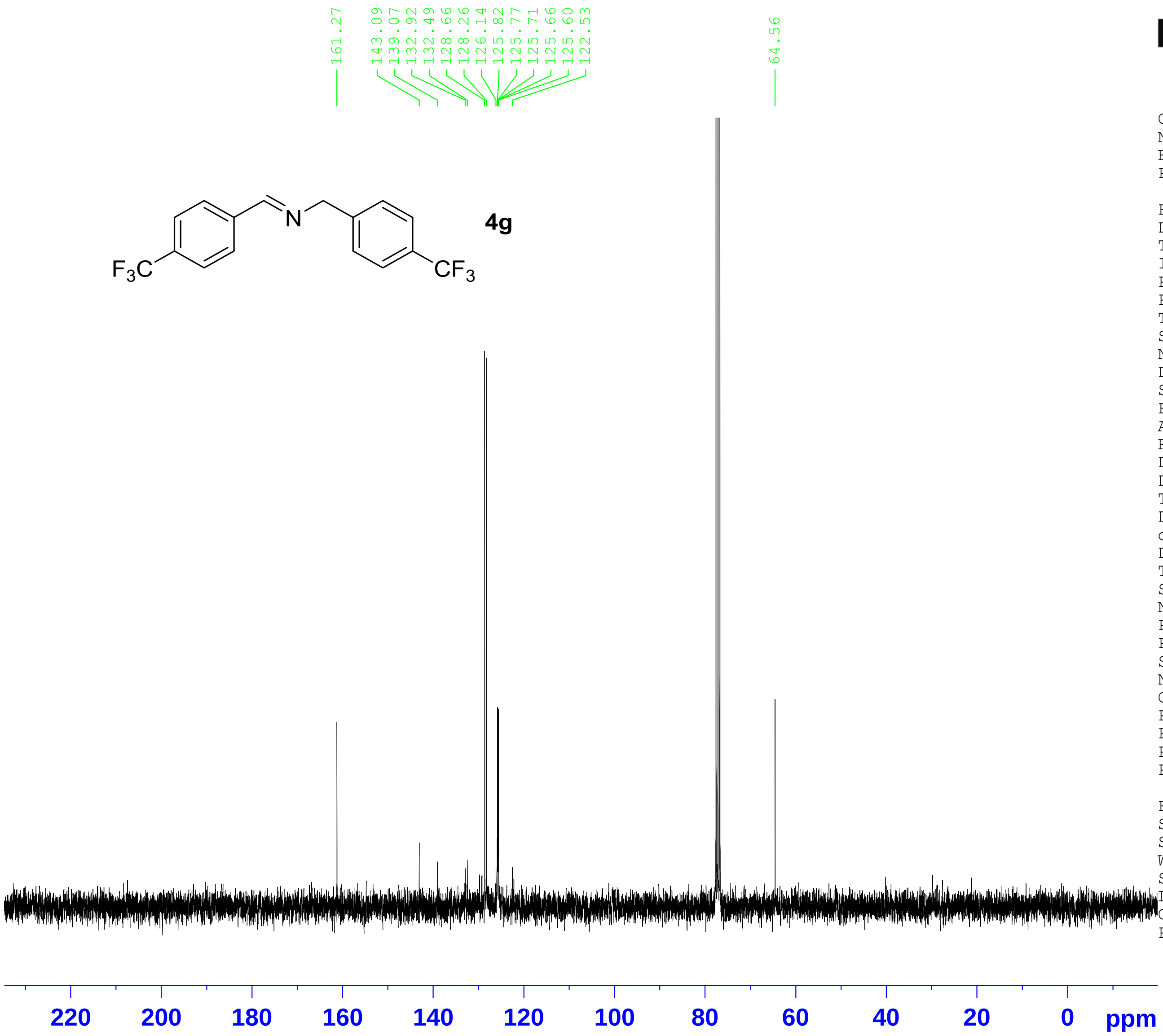

BRUKER

Current Data Parameters

NAME	Feb27-2013-md1
EXPNO	11
PROCNO	1

F2 - Acquisition Parameters

Date_	20130227
Time	15.33
INSTRUM	av300
PROBHD	5 mm BBO BB-1H
PULPROG	zgpg30
TD	65536
SOLVENT	CDCl3
NS	256
DS	2
SWH	20325.203 Hz
FIDRES	0.310138 Hz
AQ	1.6121856 sec
RG	16384
DW	24.600 usec
DE	6.00 usec
TE	673.2 K
D1	2.00000000 sec
d11	0.03000000 sec
DELTA	1.89999998 sec
TD0	1
SFO1	75.4990304 MHz
NUC1	13C
P1	7.50 usec
PLW1	-1.00000000 W
SFO2	300.2212009 MHz
NUC2	1H
CPDPRG[2	waltz16
PCPD2	80.00 usec
PLW2	-1.00000000 W
PLW12	-1.00000000 W
PLW13	-1.00000000 W

F2 - Processing parameters

SI	65536
SF	75.4903687 MHz
WDW	EM
SSB	0
LB	1.00 Hz
GB	0
PC	1.40

AM655

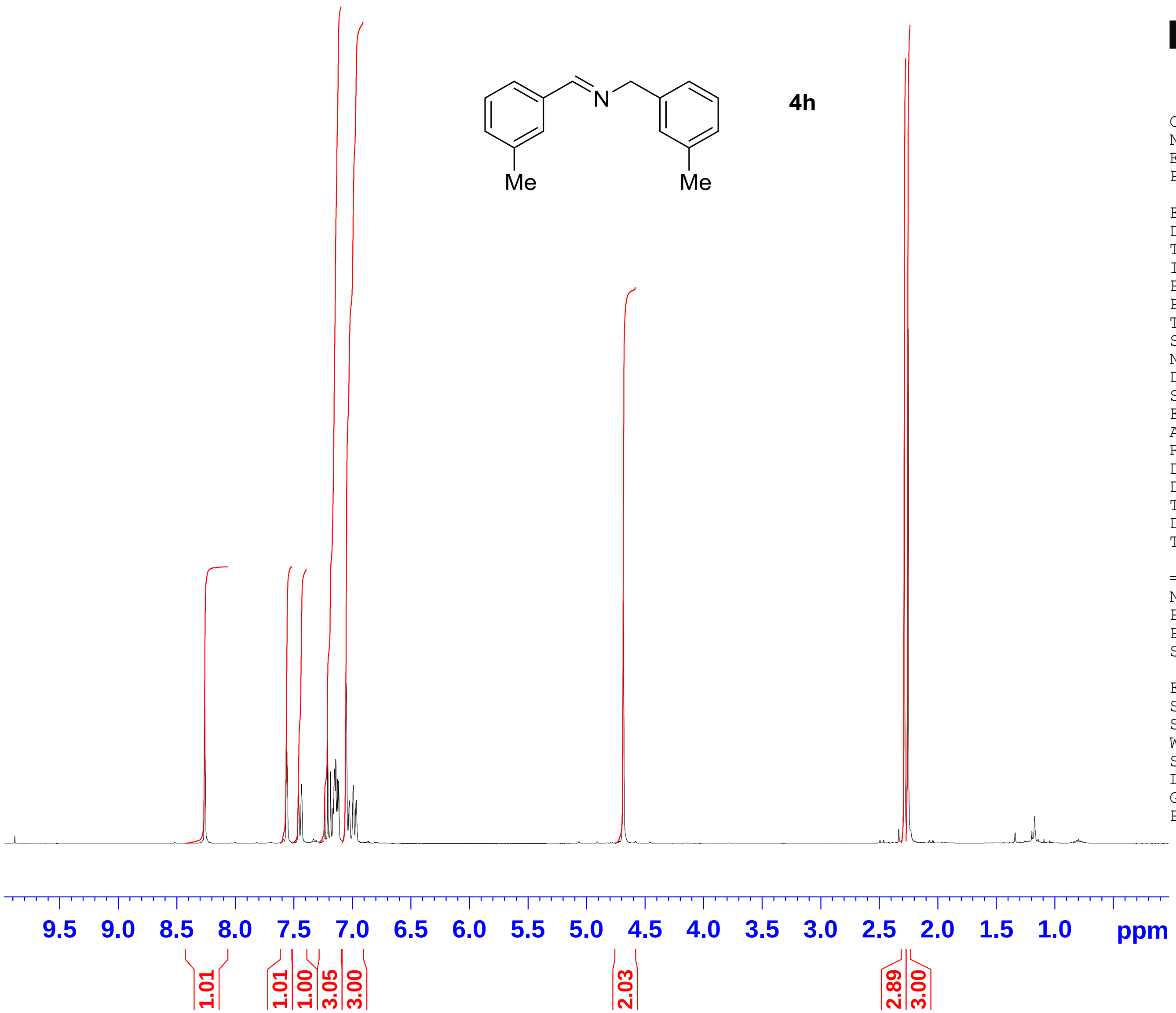

Current Data Parameters
NAME Jan24-2013
EXPNO 10
PROCNO 1

F2 - Acquisition Parameters
Date_ 20130124
Time 12.17
INSTRUM av300
PROBHD 5 mm BBO BB-1H
PULPROG zg30
TD 32768
SOLVENT CDCl3
NS 32
DS 0
SWH 6172.839 Hz
FIDRES 0.188380 Hz
AQ 2.6542079 sec
RG 114
DW 81.000 usec
DE 6.00 usec
TE 293.3 K
D1 1.00000000 sec
TD0 1

======== CHANNEL f1 ========
NUC1 1H
P1 7.10 usec
PL1 -1.50 dB
SFO1 300.2218540 MHz

F2 - Processing parameters
SI 32768
SF 300.2200419 MHz
WDW EM
SSB 0
LB 0.30 Hz
GB 0
PC 1.00

AM655

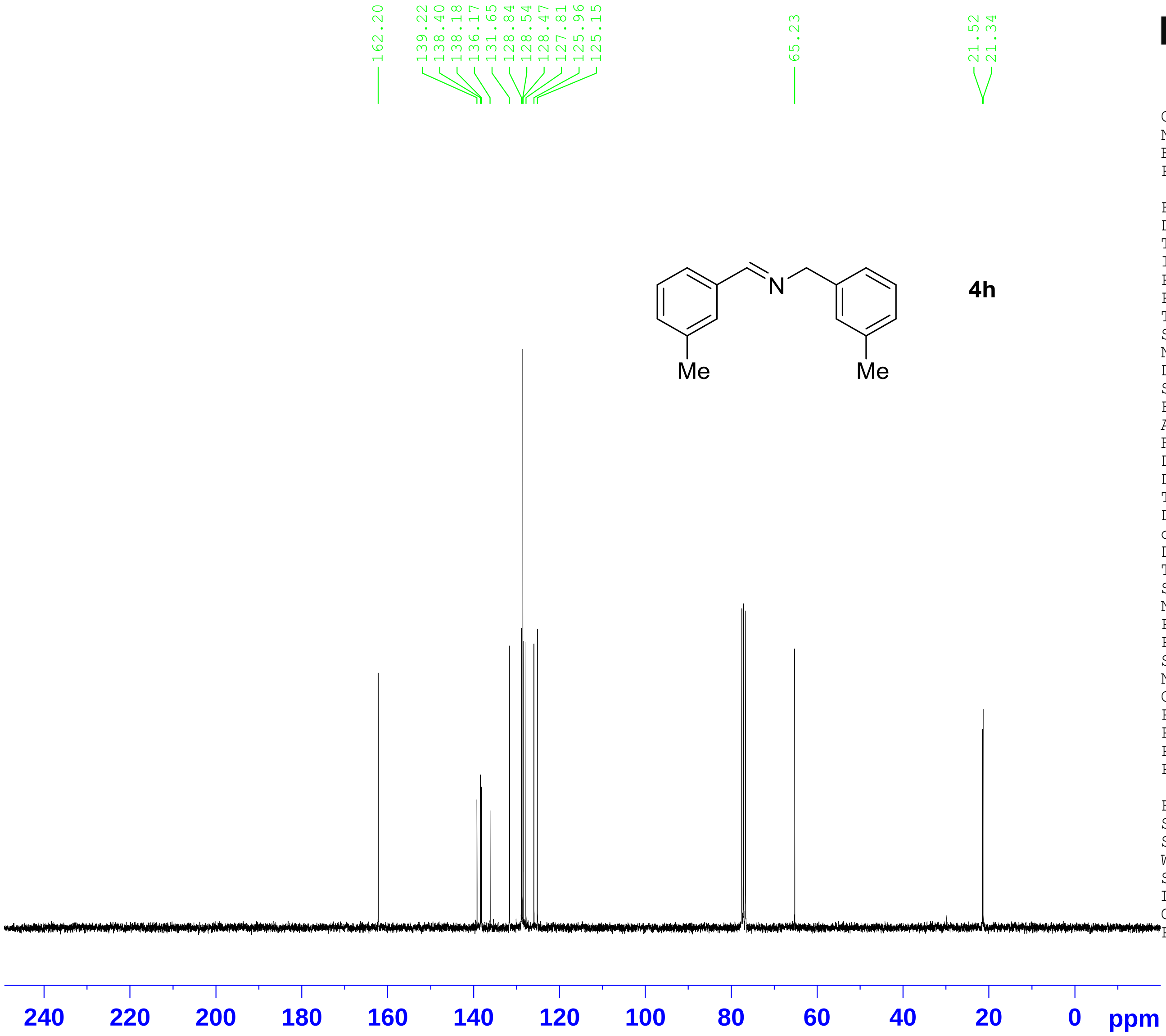

4h

Current Data Parameters
NAME Jan24-2013
EXPNO 11
PROCNO 1

F2 - Acquisition Parameters
Date_ 20130124
Time 12.35
INSTRUM av300
PROBHD 5 mm BBO BB-1H
PULPROG zgpg30
TD 65536
SOLVENT CDCl3
NS 256
DS 2
SWH 20325.203 Hz
FIDRES 0.310138 Hz
AQ 1.6121856 sec
RG 16384
DW 24.600 usec
DE 6.00 usec
TE 293.0 K
D1 2.00000000 sec
d11 0.03000000 sec
DELTA 1.89999998 sec
TD0 1
SFO1 75.4990304 MHz
NUC1 13C
P1 7.50 usec
PLW1 -1.00000000 W
SFO2 300.2212009 MHz
NUC2 1H
CPDPRG[2 waltz16
PCPD2 80.00 usec
PLW2 -1.00000000 W
PLW12 -1.00000000 W
PLW13 -1.00000000 W

F2 - Processing parameters
SI 65536
SF 75.4903769 MHz
WDW EM
SSB 0
LB 1.00 Hz
GB 0
PC 1.40

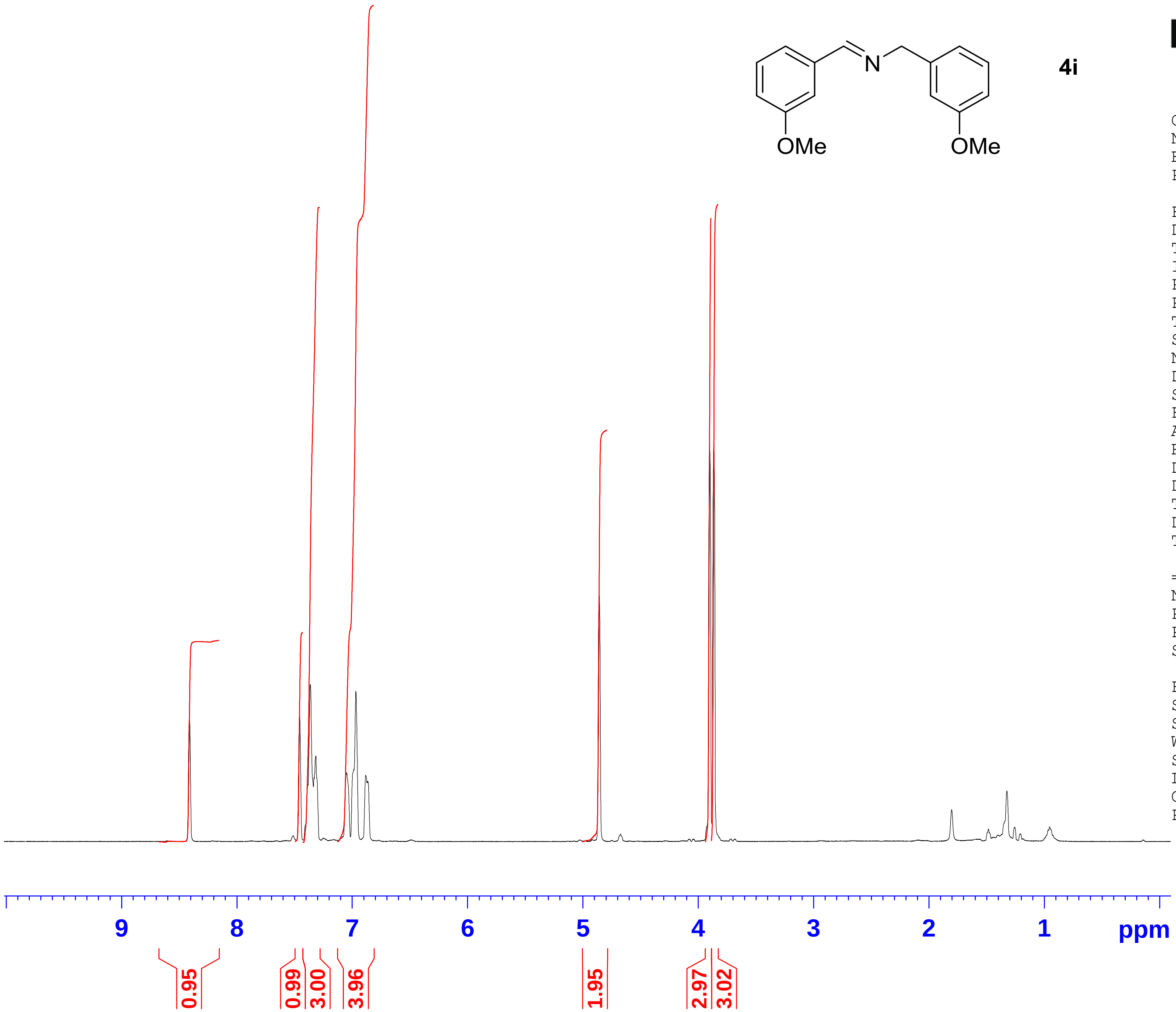

4i

Current Data Parameters
NAME AM617
EXPNO 1
PROCNO 1

F2 - Acquisition Parameters
Date_ 20130103
Time 17.15
INSTRUM spect
PROBHD 5 mm PABBO BB-
PULPROG zg30
TD 65536
SOLVENT CDCl3
NS 16
DS 0
SWH 8278.146 Hz
FIDRES 0.126314 Hz
AQ 3.9583745 sec
RG 128
DW 60.400 usec
DE 6.00 usec
TE 298.0 K
D1 1.00000000 sec
TD0 1

======== CHANNEL f1 ========
NUC1 1H
P1 10.75 usec
PL1 0 dB
SFO1 400.1324710 MHz

F2 - Processing parameters
SI 32768
SF 400.1300000 MHz
WDW no
SSB 0
LB 0 Hz
GB 0
PC 1.00

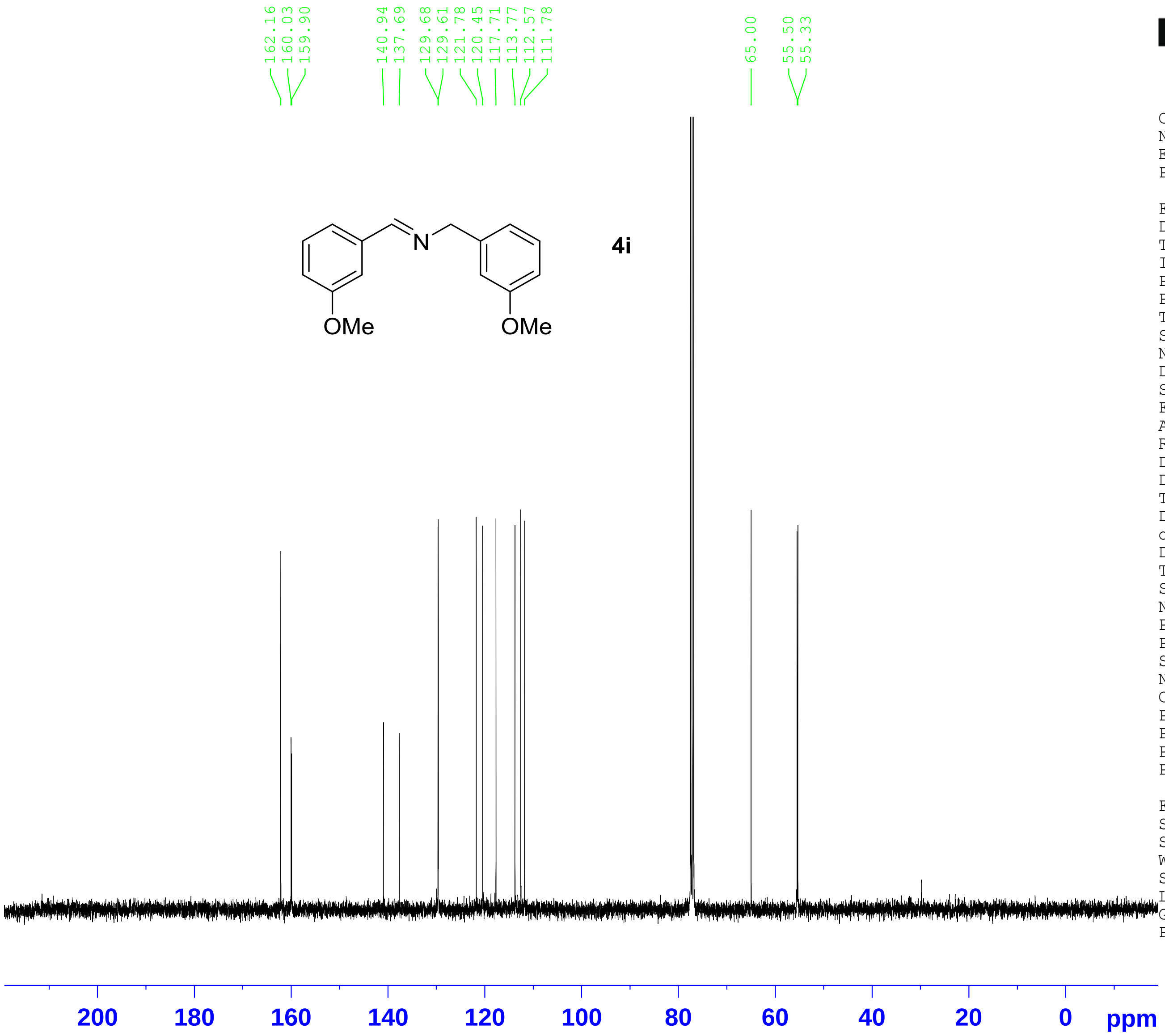


```
Current Data Parameters
NAME                AM617
EXPNO                   2
PROCNO                  1

F2 - Acquisition Parameters
Date_            20130103
Time                17.22
INSTRUM             spect
PROBHD    5 mm PABBO BB-
PULPROG           zgpg30
TD                 65536
SOLVENT            CDCl3
NS                   396
DS                     4
SWH            23980.814 Hz
FIDRES          0.365918 Hz
AQ             1.3664256 sec
RG                   512
DW                20.850 usec
DE                  6.00 usec
TE                 298.0 K
D1            2.00000000 sec
d11           0.03000000 sec
DELTA         1.89999998 sec
TD0                    1
SFO1         100.6228298 MHz
NUC1                 13C
P1                  7.30 usec
PLW1         -1.00000000 W
SFO2         400.1316005 MHz
NUC2                  1H
CPDPRG[2         waltz16
PCPD2              80.00 usec
PLW2         -1.00000000 W
PLW12        -1.00000000 W
PLW13        -1.00000000 W

F2 - Processing parameters
SI                 32768
SF           100.6127616 MHz
WDW                   EM
SSB       0
LB                  1.00 Hz
GB        0
PC                  1.40
```

AM550p

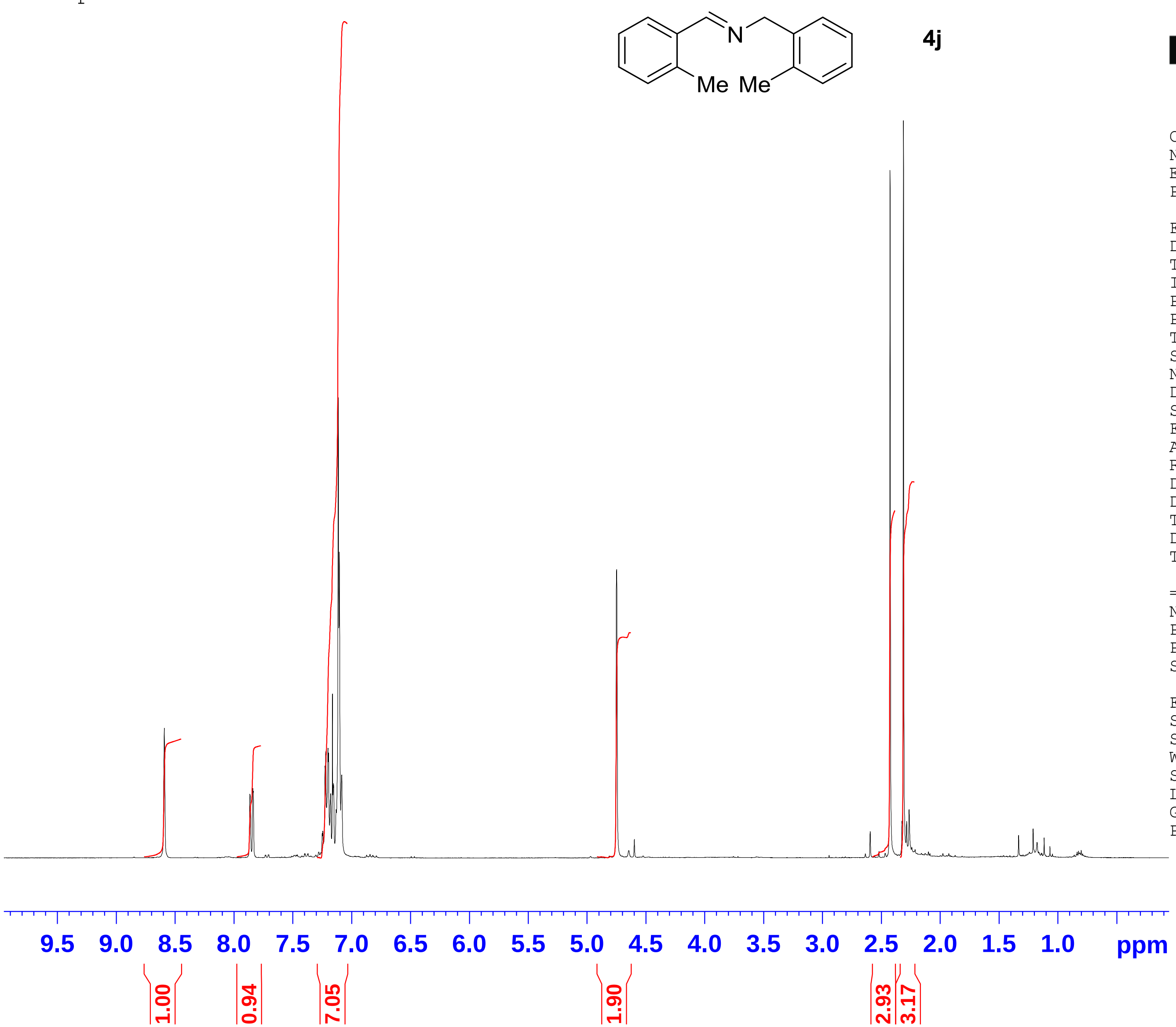

4j

```
Current Data Parameters
NAME              Nov20-2012
EXPNO                     10
PROCNO                     1

F2 - Acquisition Parameters
Date_               20121120
Time                   15.01
INSTRUM                av300
PROBHD     5 mm BBO BB-1H
PULPROG                 zg30
TD                     32768
SOLVENT                CDCl3
NS                        32
DS                         0
SWH             6172.839 Hz
FIDRES          0.188380 Hz
AQ             2.6542079 sec
RG                       181
DW               81.000 usec
DE                 6.00 usec
TE                 673.2 K
D1            1.00000000 sec
TD0                        1

======== CHANNEL f1 ========
NUC1                      1H
P1                 7.10 usec
PL1                 -1.50 dB
SFO1         300.2218540 MHz

F2 - Processing parameters
SI                     32768
SF           300.2200317 MHz
WDW                       EM
SSB          0
LB                   0.30 Hz
GB           0
PC                      1.00
```

AM550p

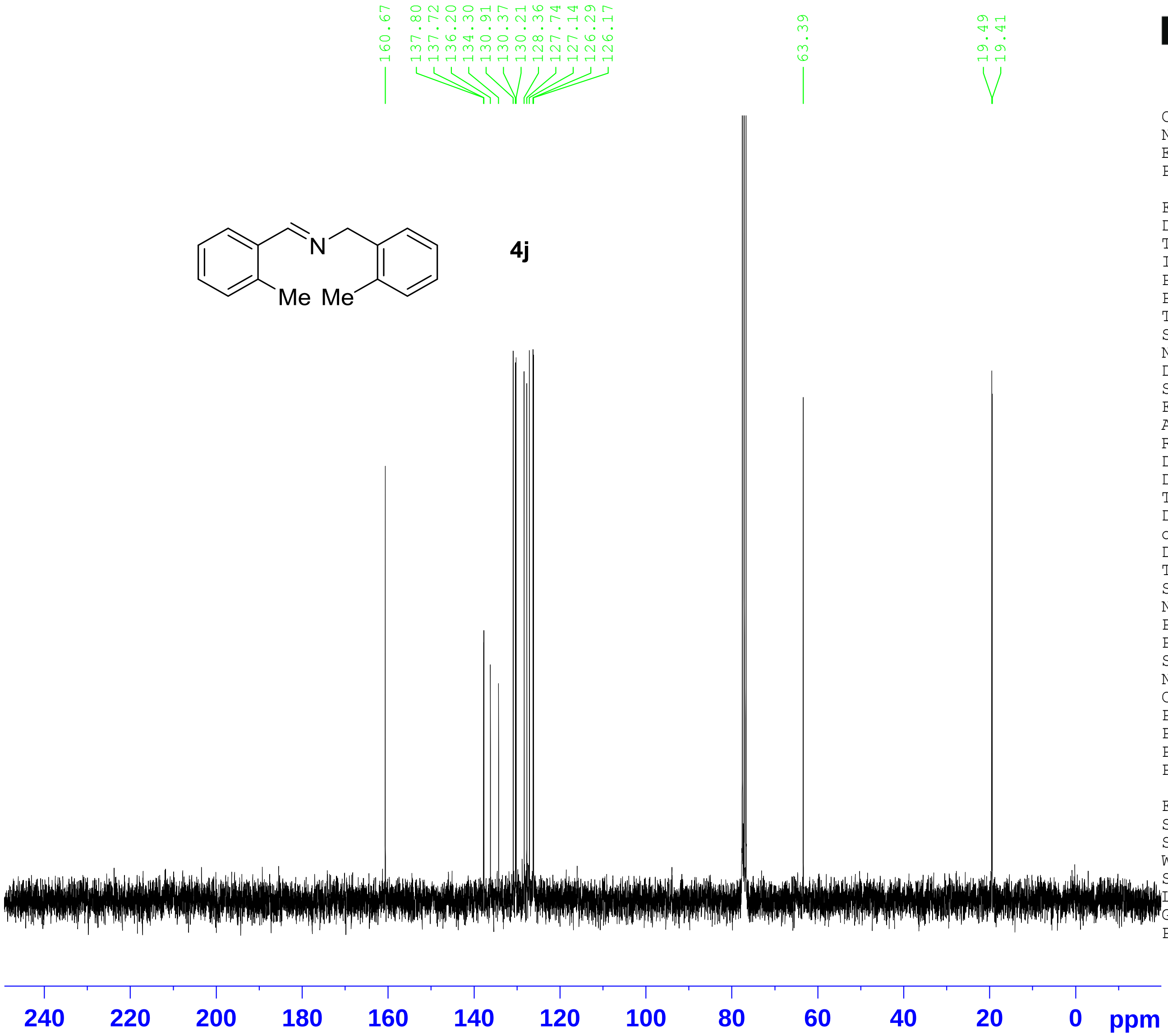

4j

Current Data Parameters
NAME Nov20-2012
EXPNO 11
PROCNO 1

F2 - Acquisition Parameters
Date_ 20121120
Time 15.19
INSTRUM av300
PROBHD 5 mm BBO BB-1H
PULPROG zgpg30
TD 65536
SOLVENT CDCl3
NS 256
DS 2
SWH 20325.203 Hz
FIDRES 0.310138 Hz
AQ 1.6121856 sec
RG 13004
DW 24.600 usec
DE 6.00 usec
TE 673.2 K
D1 2.00000000 sec
d11 0.03000000 sec
DELTA 1.89999998 sec
TD0 1
SFO1 75.4990304 MHz
NUC1 13C
P1 7.50 usec
PLW1 -1.00000000 W
SFO2 300.2212009 MHz
NUC2 1H
CPDPRG[2 waltz16
PCPD2 80.00 usec
PLW2 -1.00000000 W
PLW12 -1.00000000 W
PLW13 -1.00000000 W

F2 - Processing parameters
SI 65536
SF 75.4903730 MHz
WDW EM
SSB 0
LB 1.00 Hz
GB 0
PC 1.40

MD054

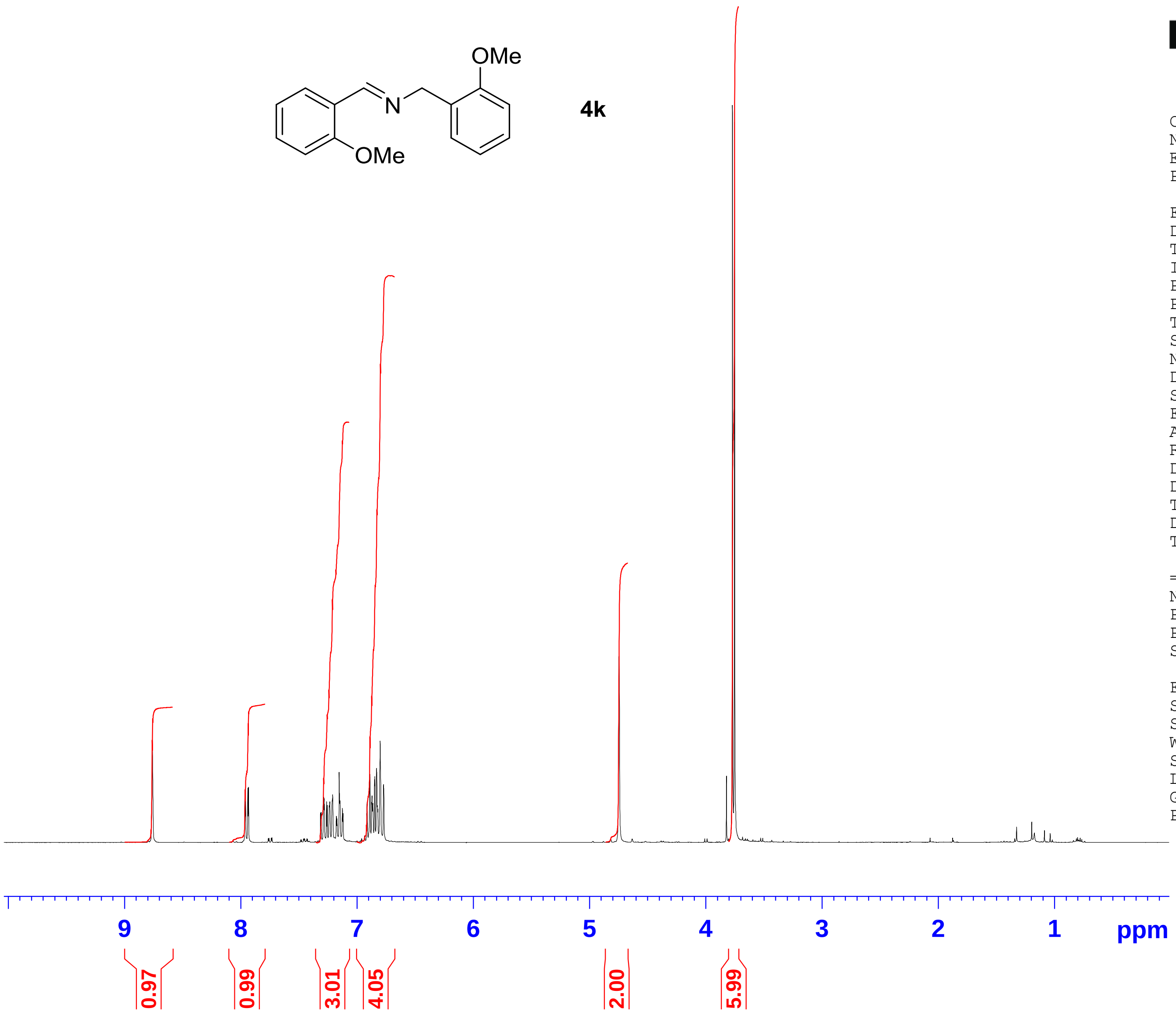


```
Current Data Parameters
NAME       Feb07-2013-md1
EXPNO                  20
PROCNO                  1

F2 - Acquisition Parameters
Date_            20130208
Time                 6.52
INSTRUM             av300
PROBHD     5 mm BBO BB-1H
PULPROG              zg30
TD                  32768
SOLVENT             CDCl3
NS                     32
DS                      0
SWH              6172.839 Hz
FIDRES           0.188380 Hz
AQ              2.6542079 sec
RG                    181
DW                 81.000 usec
DE                   6.00 usec
TE                  673.2 K
D1             1.00000000 sec
TD0                     1

======== CHANNEL f1 ========
NUC1                   1H
P1                   7.10 usec
PL1                 -1.50 dB
SFO1          300.2218540 MHz

F2 - Processing parameters
SI                  32768
SF            300.2200345 MHz
WDW                    EM
SSB        0
LB                   0.30 Hz
GB         0
PC                   1.00
```

MD054

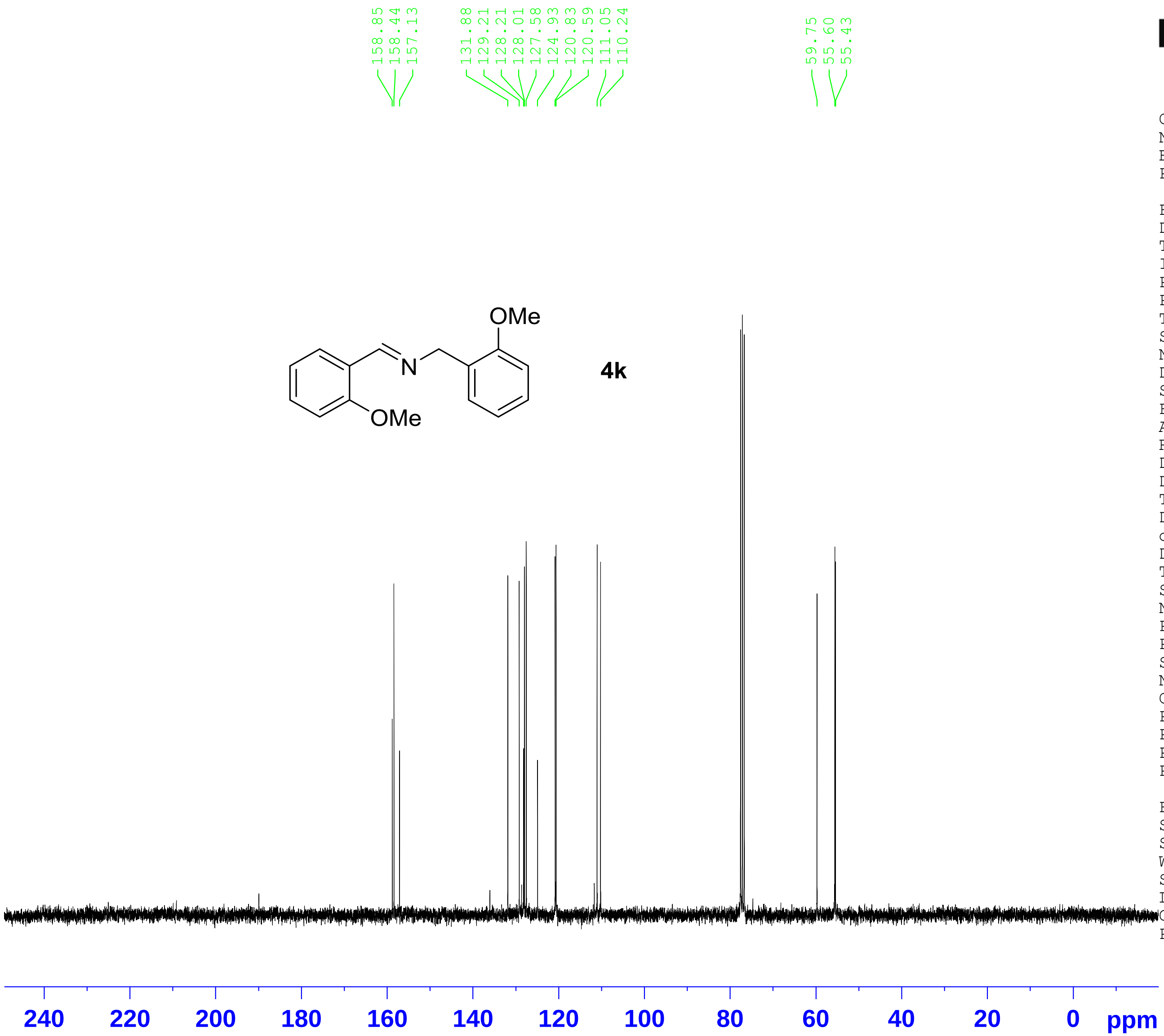

4k

BRUKER

Current Data Parameters
NAME Feb07-2013-md1
EXPNO 21
PROCNO 1

F2 - Acquisition Parameters
Date_ 20130208
Time 7.09
INSTRUM av300
PROBHD 5 mm BBO BB-1H
PULPROG zgpg30
TD 65536
SOLVENT CDCl3
NS 256
DS 2
SWH 20325.203 Hz
FIDRES 0.310138 Hz
AQ 1.6121856 sec
RG 16384
DW 24.600 usec
DE 6.00 usec
TE 673.2 K
D1 2.00000000 sec
d11 0.03000000 sec
DELTA 1.89999998 sec
TD0 1
SFO1 75.4990304 MHz
NUC1 13C
P1 7.50 usec
PLW1 -1.00000000 W
SFO2 300.2212009 MHz
NUC2 1H
CPDPRG[2 waltz16
PCPD2 80.00 usec
PLW2 -1.00000000 W
PLW12 -1.00000000 W
PLW13 -1.00000000 W

F2 - Processing parameters
SI 65536
SF 75.4903741 MHz
WDW EM
SSB 0
LB 1.00 Hz
GB 0
PC 1.40

MD058

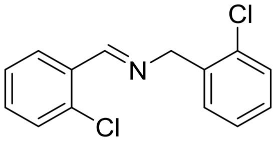

4l

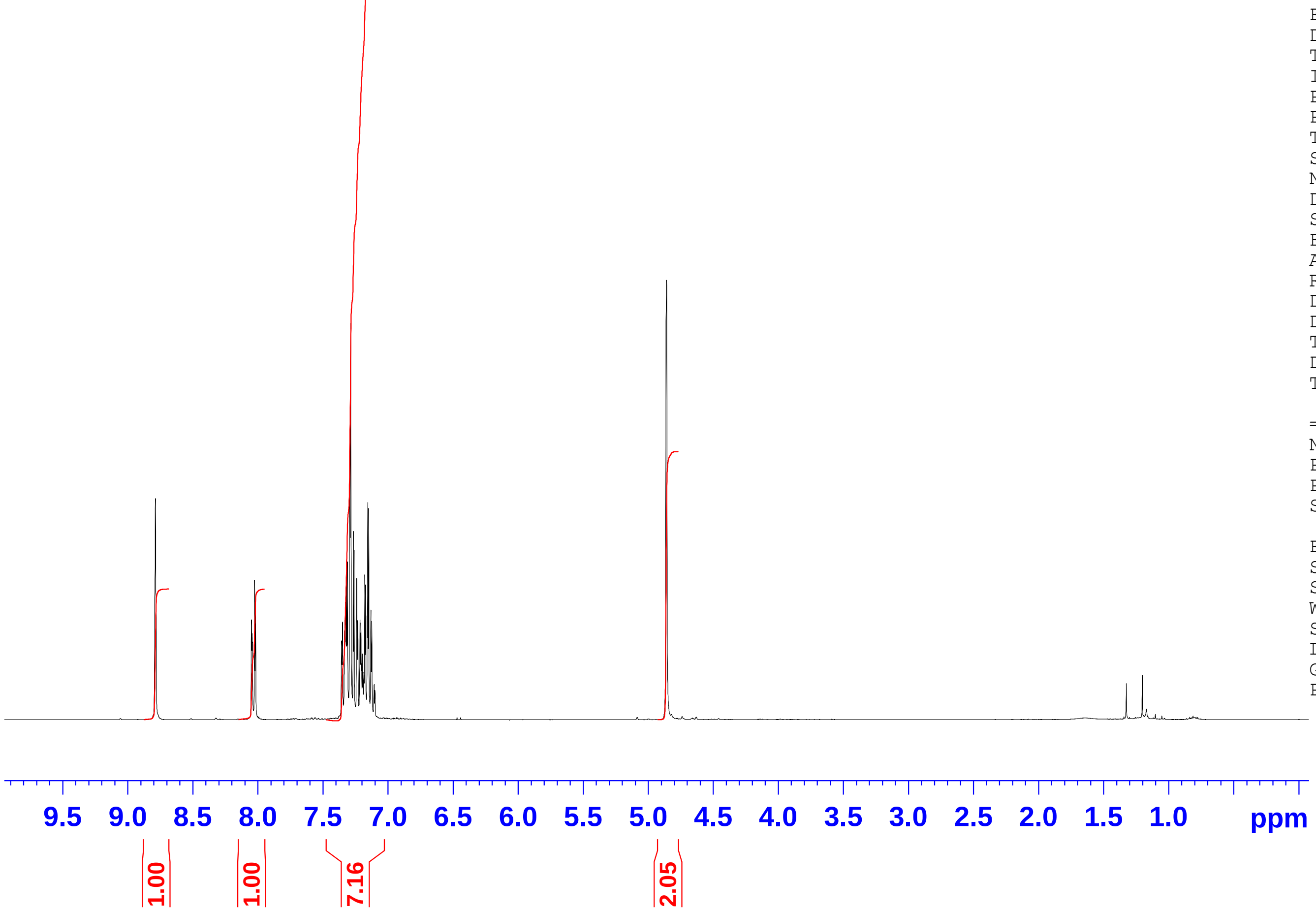

BRUKER

```
Current Data Parameters
NAME        Feb11-2013-md1
EXPNO                   20
PROCNO                   1

F2 - Acquisition Parameters
Date_             20130211
Time                 11.32
INSTRUM              av300
PROBHD     5 mm BBO BB-1H
PULPROG               zg30
TD                   32768
SOLVENT              CDCl3
NS                      32
DS                       0
SWH               6172.839 Hz
FIDRES            0.188380 Hz
AQ               2.6542079 sec
RG                     181
DW                  81.000 usec
DE                    6.00 usec
TE                   673.2 K
D1              1.00000000 sec
TD0                      1

======== CHANNEL f1 ========
NUC1                    1H
P1                    7.10 usec
PL1                  -1.50 dB
SFO1           300.2218540 MHz

F2 - Processing parameters
SI                   32768
SF             300.2200324 MHz
WDW                     EM
SSB        0
LB                    0.30 Hz
GB         0
PC                    1.00
```

MD058

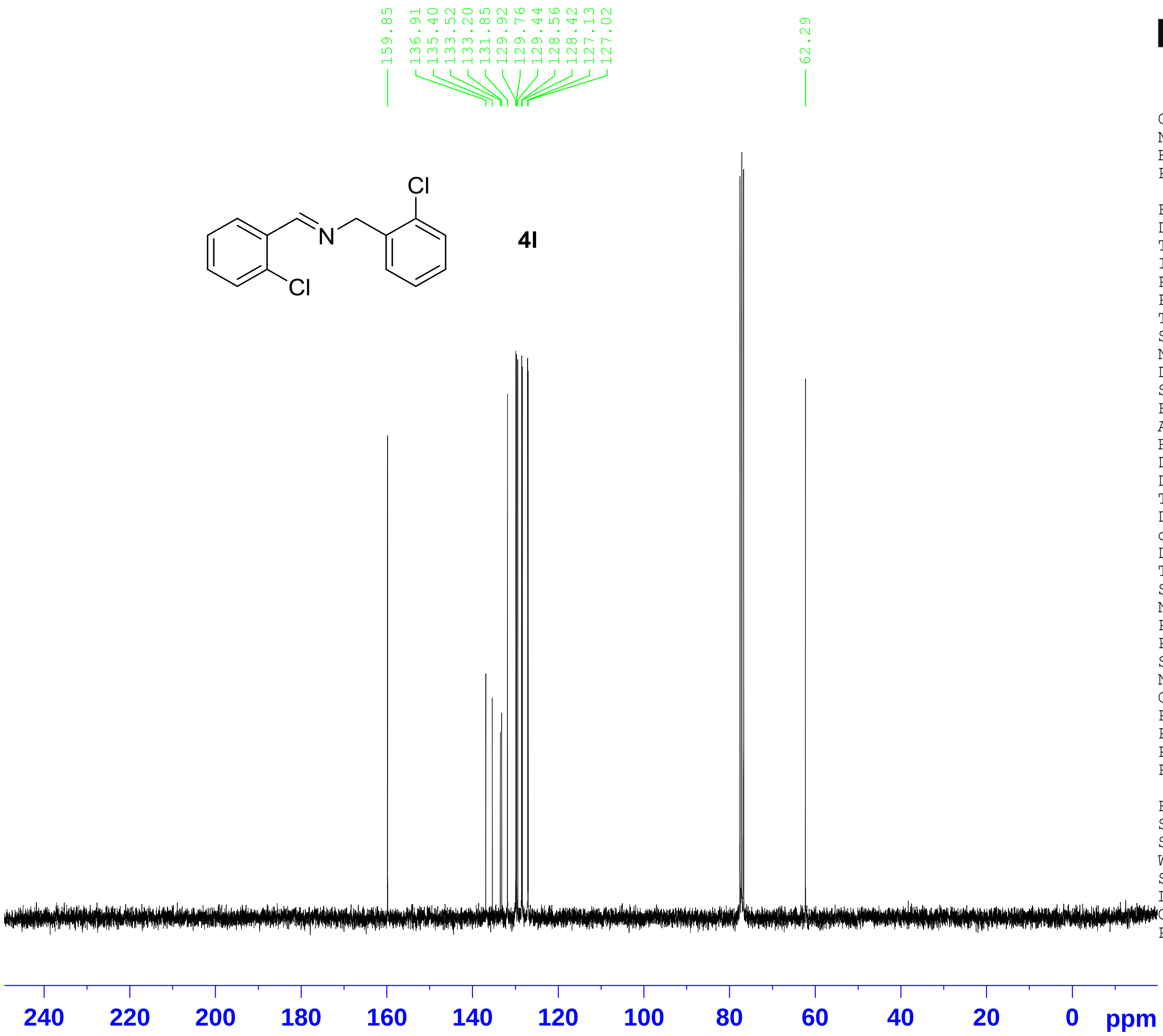

BRUKER

```
Current Data Parameters
NAME      Feb11-2013-md1
EXPNO                 21
PROCNO                 1

F2 - Acquisition Parameters
Date_           20130211
Time               11.49
INSTRUM            av300
PROBHD    5 mm BBO BB-1H
PULPROG          zgpg30
TD                 65536
SOLVENT            CDCl3
NS                   256
DS                     2
SWH            20325.203 Hz
FIDRES          0.310138 Hz
AQ             1.6121856 sec
RG                9195.2
DW                24.600 usec
DE                  6.00 usec
TE                 673.2 K
D1            2.00000000 sec
d11           0.03000000 sec
DELTA         1.89999998 sec
TD0                    1
SFO1          75.4990304 MHz
NUC1                 13C
P1                  7.50 usec
PLW1         -1.00000000 W
SFO2         300.2212009 MHz
NUC2                  1H
CPDPRG[2         waltz16
PCPD2              80.00 usec
PLW2         -1.00000000 W
PLW12        -1.00000000 W
PLW13        -1.00000000 W

F2 - Processing parameters
SI                 65536
SF            75.4903729 MHz
WDW                   EM
SSB       0
LB                  1.00 Hz
GB        0
PC                  1.40
```

MD047

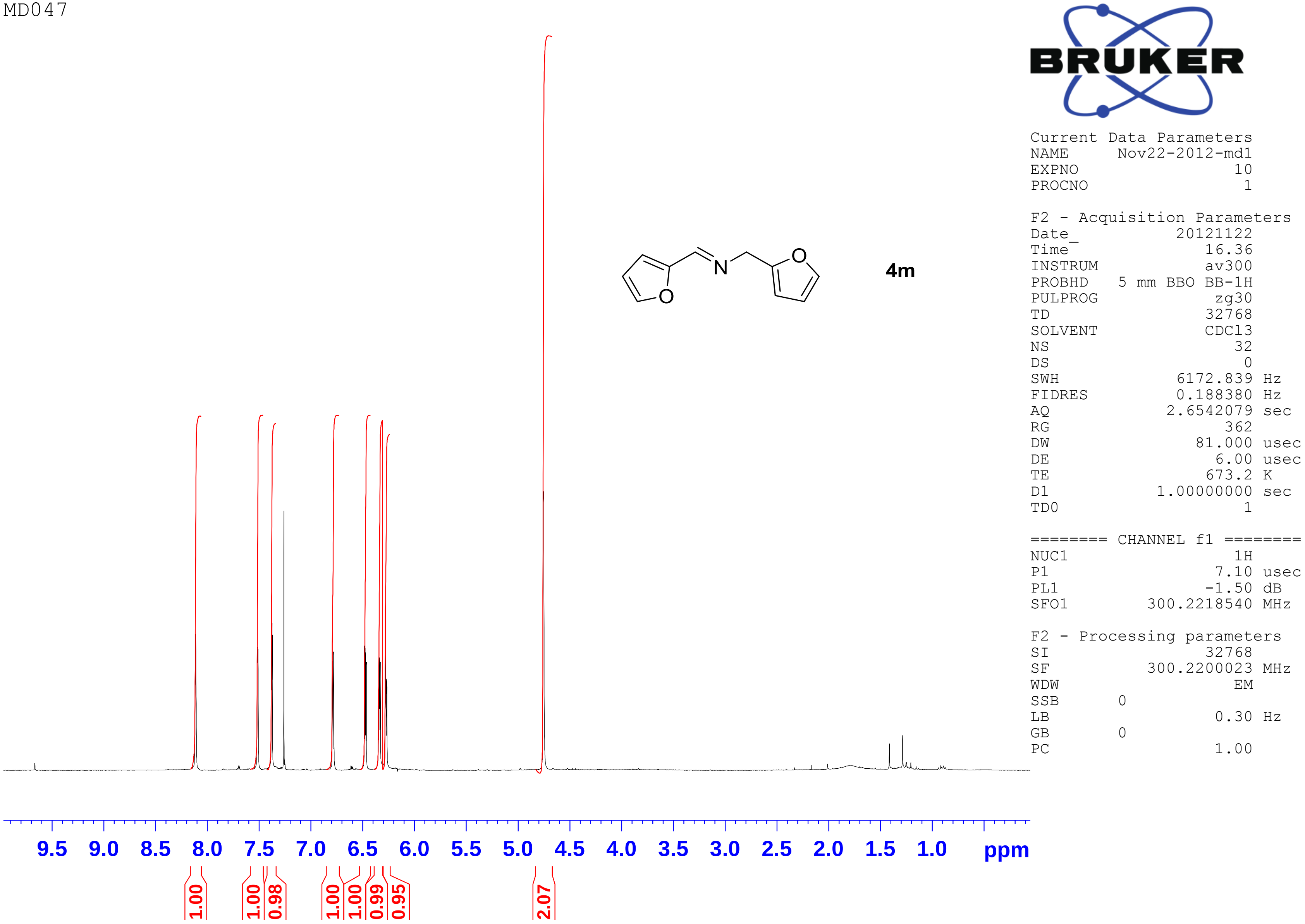

4m
Current Data Parameters
NAME Nov22-2012-md1
EXPNO 10
PROCNO 1
F2 - Acquisition Parameters
Date_ 20121122
Time 16.36
INSTRUM av300
PROBHD 5 mm BBO BB-1H
PULPROG zg30
TD 32768
SOLVENT CDCl3
NS 32
DS 0
SWH 6172.839 Hz
FIDRES 0.188380 Hz
AQ 2.6542079 sec
RG 362
DW 81.000 usec
DE 6.00 usec
TE 673.2 K
D1 1.00000000 sec
TD0 1
======== CHANNEL f1 ========
NUC1 1H
P1 7.10 usec
PL1 -1.50 dB
SFO1 300.2218540 MHz
F2 - Processing parameters
SI 32768
SF 300.2200023 MHz
WDW EM
SSB 0
LB 0.30 Hz
GB 0
PC 1.00
9.5 9.0 8.5 8.0 7.5 7.0 6.5 6.0 5.5 5.0 4.5 4.0 3.5 3.0 2.5 2.0 1.5 1.0 ppm
1.00 1.00 0.98 1.00 1.00 0.99 0.95 2.07

MD047

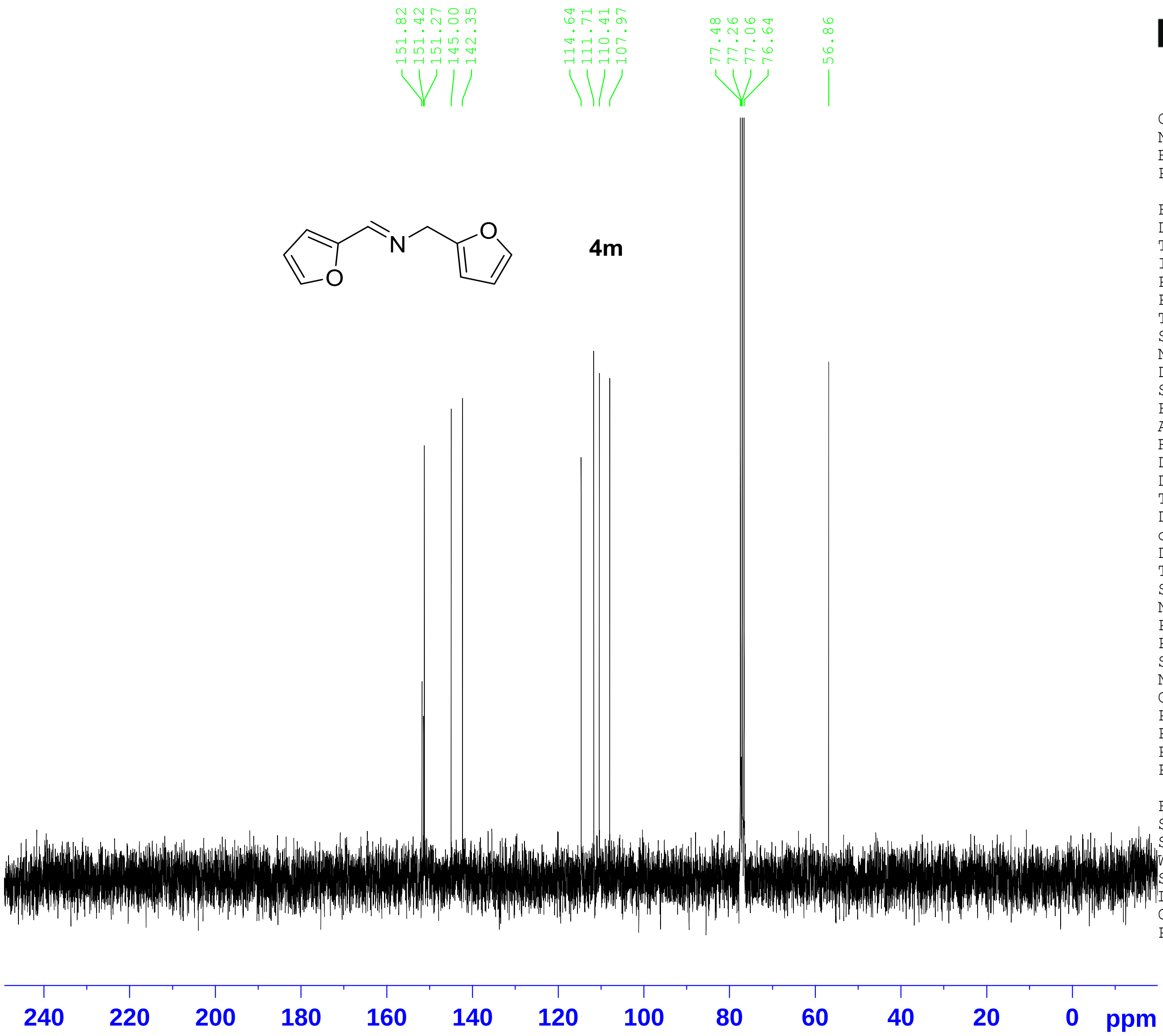

BRUKER

Current Data Parameters
NAME Nov22-2012-md1
EXPNO 11
PROCNO 1

F2 - Acquisition Parameters
Date_ 20121122
Time 16.53
INSTRUM av300
PROBHD 5 mm BBO BB-1H
PULPROG zgpg30
TD 65536
SOLVENT CDCl3
NS 256
DS 2
SWH 20325.203 Hz
FIDRES 0.310138 Hz
AQ 1.6121856 sec
RG 20642.5
DW 24.600 usec
DE 6.00 usec
TE 673.2 K
D1 2.00000000 sec
d11 0.03000000 sec
DELTA 1.89999998 sec
TD0 1
SFO1 75.4990304 MHz
NUC1 13C
P1 7.50 usec
PLW1 -1.00000000 W
SFO2 300.2212009 MHz
NUC2 1H
CPDPRG[2 waltz16
PCPD2 80.00 usec
PLW2 -1.00000000 W
PLW12 -1.00000000 W
PLW13 -1.00000000 W

F2 - Processing parameters
SI 65536
SF 75.4903777 MHz
WDW EM
SSB 0
LB 1.00 Hz
GB 0
PC 1.40

AM642

4n

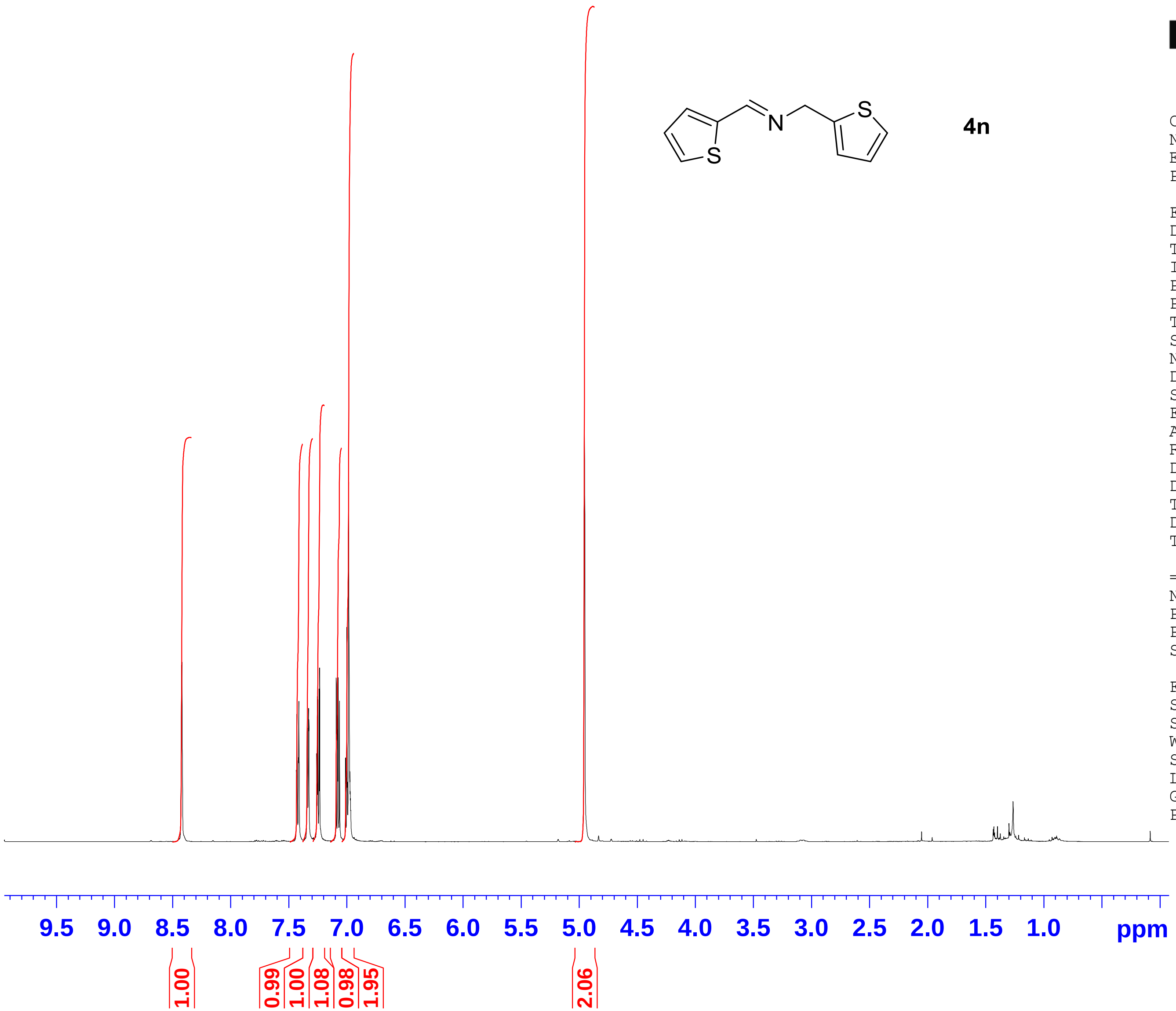


```
Current Data Parameters
NAME              Jan17-2013
EXPNO                     10
PROCNO                     1

F2 - Acquisition Parameters
Date_               20130117
Time                   12.15
INSTRUM                av300
PROBHD     5 mm BBO BB-1H
PULPROG                 zg30
TD                     32768
SOLVENT                CDCl3
NS                        32
DS                         0
SWH                 6172.839 Hz
FIDRES              0.188380 Hz
AQ                 2.6542079 sec
RG                       181
DW                    81.000 usec
DE                      6.00 usec
TE                     292.2 K
D1                1.00000000 sec
TD0                        1

======== CHANNEL f1 ========
NUC1                      1H
P1                      7.10 usec
PL1                    -1.50 dB
SFO1             300.2218540 MHz

F2 - Processing parameters
SI                     32768
SF               300.2200032 MHz
WDW                       EM
SSB       0
LB                      0.30 Hz
GB        0
PC                      1.00
```

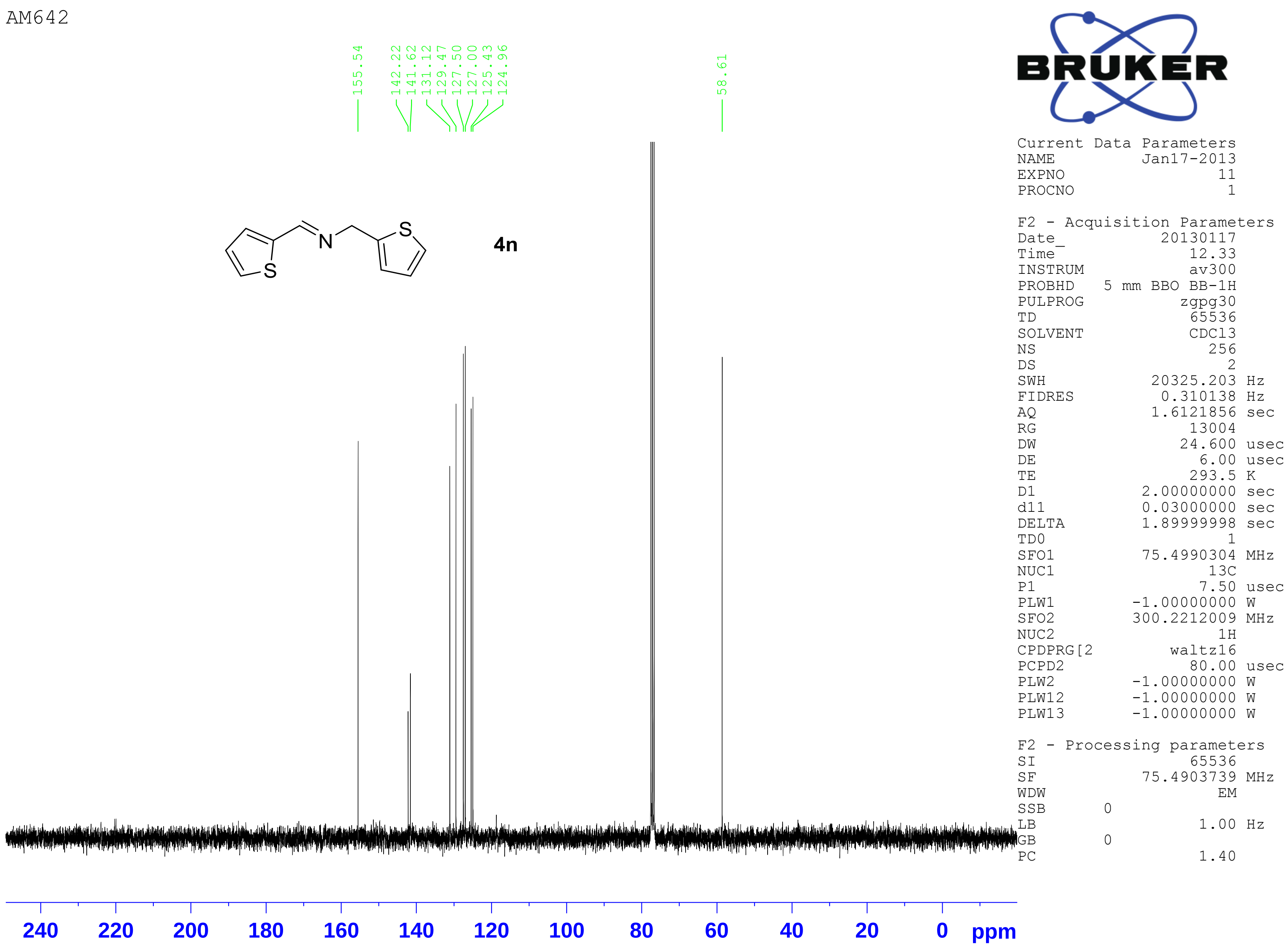
AM642
155.54
142.22
141.62
131.12
129.47
127.50
127.00
125.43
124.96
58.61
S
N
S
4n
BRUKER
Current Data Parameters
NAME Jan17-2013
EXPNO 11
PROCNO 1
F2 - Acquisition Parameters
Date_ 20130117
Time 12.33
INSTRUM av300
PROBHD 5 mm BBO BB-1H
PULPROG zgpg30
TD 65536
SOLVENT CDCl3
NS 256
DS 2
SWH 20325.203 Hz
FIDRES 0.310138 Hz
AQ 1.6121856 sec
RG 13004
DW 24.600 usec
DE 6.00 usec
TE 293.5 K
D1 2.00000000 sec
d11 0.03000000 sec
DELTA 1.89999998 sec
TD0 1
SFO1 75.4990304 MHz
NUC1 13C
P1 7.50 usec
PLW1 -1.00000000 W
SFO2 300.2212009 MHz
NUC2 1H
CPDPRG[2 waltz16
PCPD2 80.00 usec
PLW2 -1.00000000 W
PLW12 -1.00000000 W
PLW13 -1.00000000 W
F2 - Processing parameters
SI 65536
SF 75.4903739 MHz
WDW EM
SSB 0
LB 1.00 Hz
GB 0
PC 1.40
240 220 200 180 160 140 120 100 80 60 40 20 0 ppm

MD042

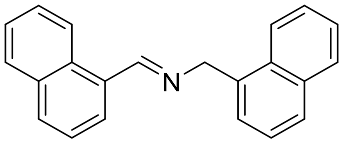

4o

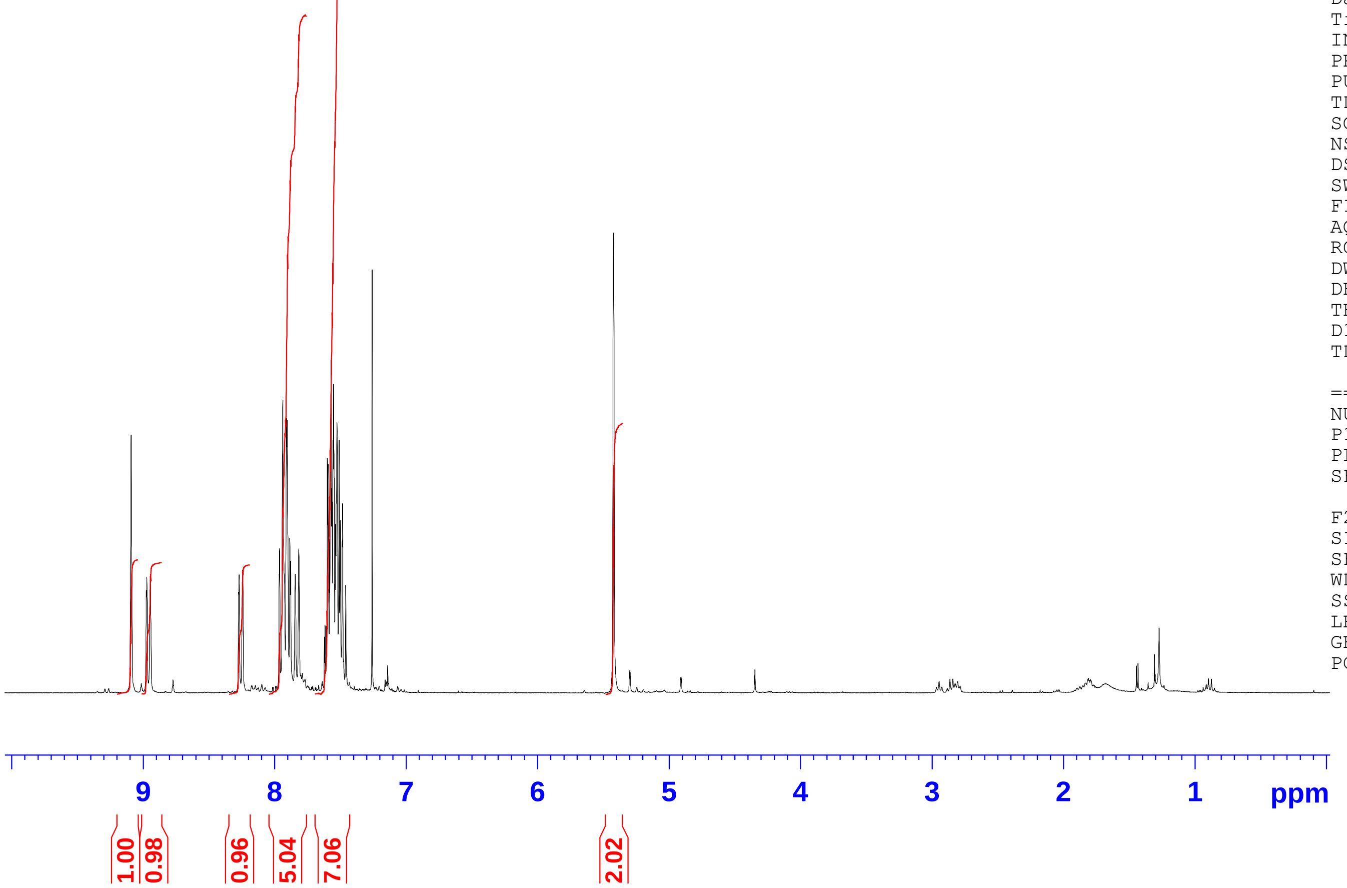

BRUKER

Current Data Parameters
NAME Nov16-2012
EXPNO 20
PROCNO 1

F2 - Acquisition Parameters
Date_ 20121116
Time 23.00
INSTRUM av300
PROBHD 5 mm BBO BB-1H
PULPROG zg30
TD 32768
SOLVENT CDCl3
NS 32
DS 0
SWH 6172.839 Hz
FIDRES 0.188380 Hz
AQ 2.6542079 sec
RG 287.4
DW 81.000 usec
DE 6.00 usec
TE 673.2 K
D1 1.00000000 sec
TD0 1

======== CHANNEL f1 ========
NUC1 1H
P1 7.10 usec
PL1 -1.50 dB
SFO1 300.2218540 MHz

F2 - Processing parameters
SI 32768
SF 300.2200029 MHz
WDW EM
SSB 0
LB 0.30 Hz
GB 0
PC 1.00

MD042

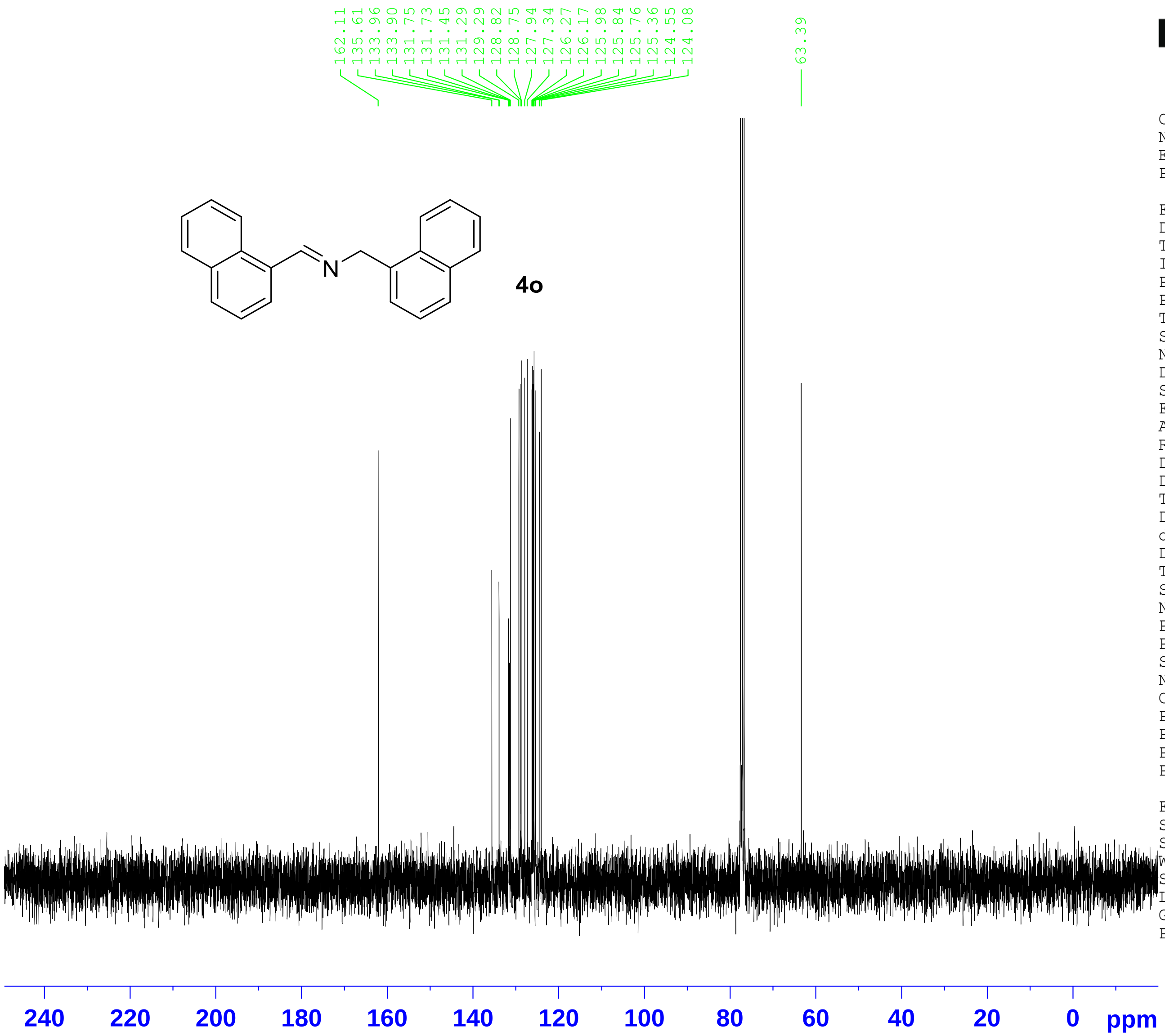

Current Data Parameters

NAME	Nov16-2012
EXPNO	21
PROCNO	1

F2 - Acquisition Parameters

Date_	20121116
Time	23.17
INSTRUM	av300
PROBHD	5 mm BBO BB-1H
PULPROG	zgpg30
TD	65536
SOLVENT	CDCl3
NS	256
DS	2
SWH	20325.203 Hz
FIDRES	0.310138 Hz
AQ	1.6121856 sec
RG	18390.4
DW	24.600 usec
DE	6.00 usec
TE	673.2 K
D1	2.00000000 sec
d11	0.03000000 sec
DELTA	1.89999998 sec
TD0	1
SFO1	75.4990304 MHz
NUC1	13C
P1	7.50 usec
PLW1	-1.00000000 W
SFO2	300.2212009 MHz
NUC2	1H
CPDPRG[2	waltz16
PCPD2	80.00 usec
PLW2	-1.00000000 W
PLW12	-1.00000000 W
PLW13	-1.00000000 W

F2 - Processing parameters

SI	65536
SF	75.4903707 MHz
WDW	EM
SSB	0
LB	1.00 Hz
GB	0
PC	1.40

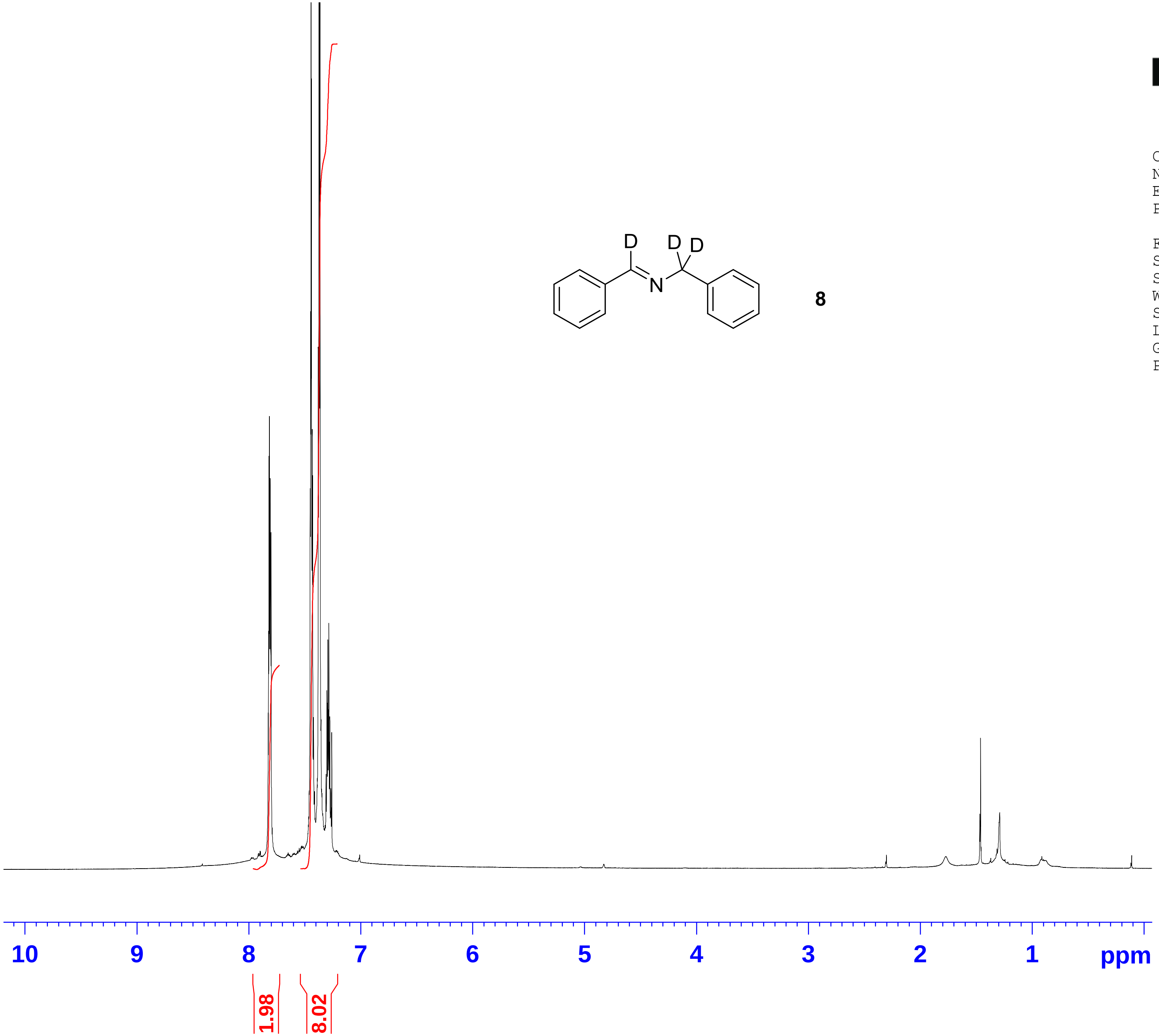


```
Current Data Parameters
NAME       AM1331 PROTON_01.fid
EXPNO                         1
PROCNO                        1

F2 - Processing parameters
SI                        32768
SF              500.0586932 MHz
WDW                          no
SSB        0
LB         0 Hz
GB         0
PC                         1.00
```

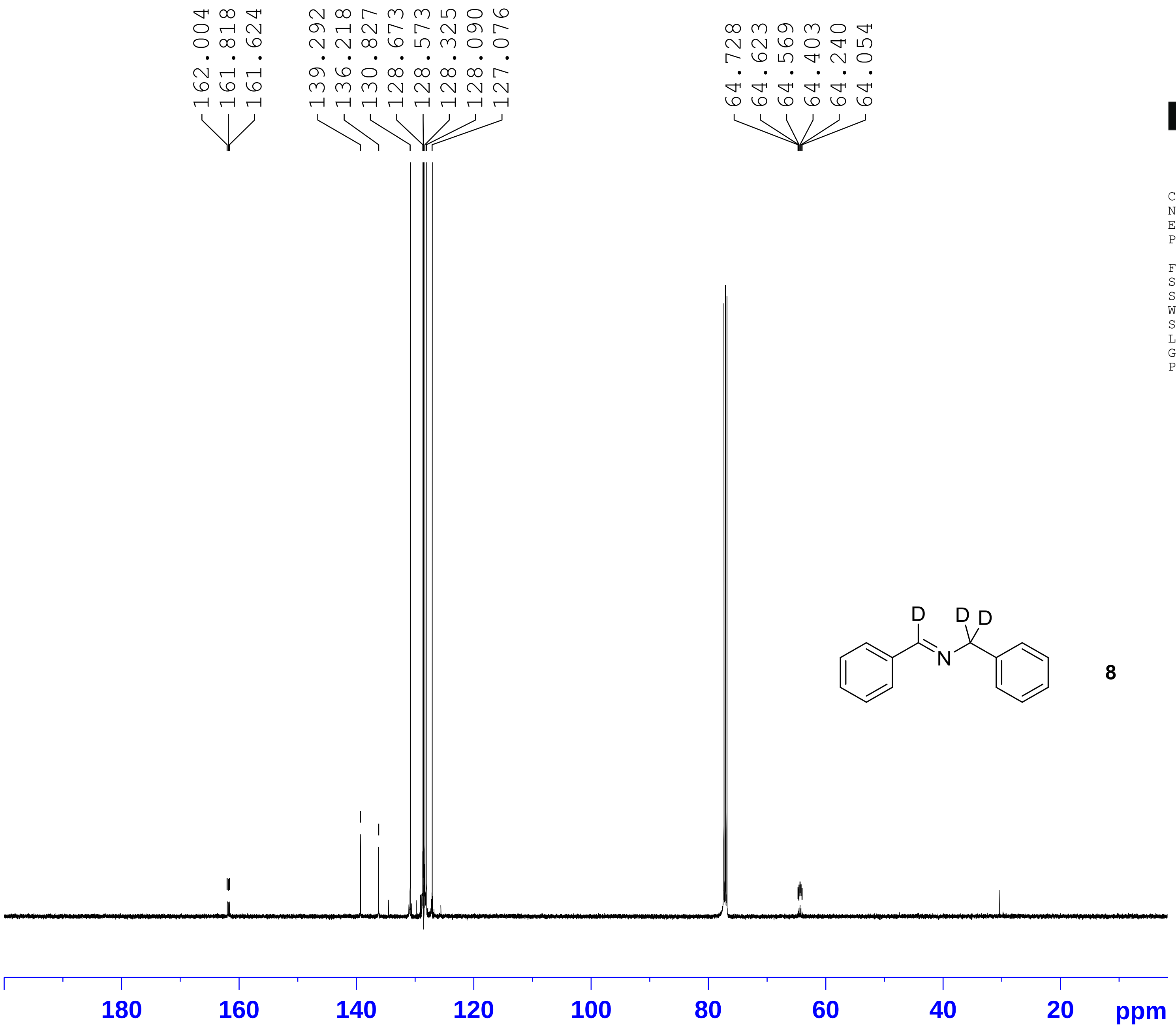

8

```
Current Data Parameters
NAME       AM1331 2c rer CARBON_01.fid
EXPNO                        1
PROCNO                       1

F2 - Processing parameters
SI                       65536
SF              125.7416369 MHz
WDW                         EM
SSB        0
LB                       0.30 Hz
GB         0
PC                       1.00
```

REFERENCES

[1] a) W.-S. Li, N. Zhang, L. M. Sayre, *Tetrahedron* **2001**, *57*, 4507-4522; b) B. J. Marsh, D. R. Carbery, *Tetrahedron Lett.* **2010**, *51*, 2362-2365.
[2] A. E. Wendlandt, S. S. Stahl, *Org. Lett.* **2012**, *14*, 2850-2853.
[3] E. Zhang, H. Tian, S. Xu, X. Yu, Q. Xu, *Org. Lett.* **2013**, *15*, 2704-2707.
[4] M. Largeron, M.-B. Fleury, *Angew. Chem. Int. Ed.* **2012**, *51*, 5409-5412.
[5] X. Lang, H. Ji, C. Chen, W. Ma, J. Zhao, *Angew. Chem. Int. Ed.* **2011**, *50*, 3934-3937.
[6] D. Armesto, S. Esteban, W. M. Horspool, J.-A. F. Martin, P. Martinet-Alcazar, R. Perez-Ossorioa, *Journal of the Chemical Society, Perkin Transactions 1* **1989**, 751-755.
[7] Y. Otsuji, S. Wake, E. Imoto, *Tetrahedron* **1970**, *26*, 4139-4152.
[8] G. Bryon Gill, M. S. H. Idris, *Tetrahedron* **1993**, *49*, 219-234.
[9] A. Carrington, A. D. MacLachlan, *Introduction to Magnetic Resonance*, Chapman and Hall, London, **1979**.
[10] S. A. Dikanov, C. A. R., in *Handbook of Applied Solid State Spectroscopy* (Ed.: V. D. R.), Springer Science + Business Media, LLC, NY, USA, **2006**, pp. 97-149.
[11] P. Höfer, A. Grupp, H. Nebenführ, M. Mehring, *Chem. Phys. Lett.* **1986**, *132*, 279-282.
[12] S. A. Dikanov, Y. D. Tsevtkov, *Electron Spin Echo Envelope Modulation (ESEEM) Spectroscopy*, CRC Press, Boca Raton, **1992**.
[13] S. A. Dikanov, Y. D. Tsvetkov, M. K. Bowman, A. V. Astashkin, *Chem. Phys. Lett.* **1982**, *90*, 149-153.
[14] H. L. Flanagan, D. J. Singel, *The Journal of Chemical Physics* **1987**, *87*, 5606-5616.
[15] V. Blum, R. Gehrke, F. Hanke, P. Havu, V. Havu, X. Ren, K. Reuter, M. Scheffler, *Comput. Phys. Commun.* **2009**, *180*, 2175-2196.
[16] J. P. Perdew, K. Burke, M. Ernzerhof, *Phys. Rev. Lett.* **1996**, *77*, 3865-3868.
[17] C. Adamo, V. Barone, *The Journal of Chemical Physics* **1999**, *110*, 6158-6170.
[18] C. H. Hendon, D. R. Carbery, A. Walsh, *Chem. Sci.* **2014**, *5*, 1390-1395.
[19] N. Mathivanan, L. J. Johnston, D. D. M. Wayner, *The Journal of Physical Chemistry* **1995**, *99*, 8190-8195.
